# Deviations from genetic additivity driven by rare variants at biobank scale

**DOI:** 10.1038/s41467-026-76151-w

**Published:** 2026-08-29

**Authors:** Frederik H. Lassen, Samvida S. Venkatesh, Nikolas A. Baya, Cecilia M. Lindgren, Duncan S. Palmer

**Affiliations:** 1 Centre for Human Genetics, University of Oxford, Oxford, United Kingdom; 2 Big Data Institute, Li Ka Shing Centre for Health Information and Discovery, University of Oxford, Oxford, United Kingdom; 3 Department of Statistics, University of Oxford, Oxford, United Kingdom; 4 Nuffield Department of Population Health, Medical Sciences Division, University of Oxford, Oxford, United Kingdom; 5 Program in Medical and Population Genetics, Broad Institute of MIT and Harvard, Cambridge, MA USA; 6 The Pioneer Centre for SMARTbiomed, Big Data Institute, Li Ka Shing Centre for Health Information and Discovery, University of Oxford, Oxford, UK

**Keywords:** Rare variants, Genome-wide association studies, Statistical methods

## Abstract

Additive genetic models are the default for genome-wide association studies, but deviations from additivity are crucial for understanding disease mechanisms and therapeutic responses. Yet existing methods for testing nonadditivity are computationally infeasible for large-scale analysis or rely on Hardy-Weinberg assumptions, making them unsuitable for rare variants in large biobanks. We use an orthogonal allelic recoding framework that enables scalable testing of nonadditive genetic effects without Hardy-Weinberg assumptions while integrating seamlessly with existing linear mixed models. Analyzing up to 399,943 UK Biobank individuals across 2906 plasma proteins and 55 quantitative traits, we demonstrate that approximately one-third of cognate gene-protein relationships exhibit nonlinear dose responses. We identify recessive effects, including *FUT10* variants associated with reduced lung function, and validate these findings in the All of Us cohort. We show that many complex traits show partial recessivity, reconciling conflicting inheritance patterns in the literature, and that joint two-degree-of-freedom models substantially improve statistical power when the underlying trait is partially recessive. Our framework efficiently partitions additive and nonadditive heritability components at biobank scale, providing a quantitative map of gene dose-response relationships with direct implications for therapeutic target identification and precision medicine.

## Abstract (web summary)

Testing nonadditive genetic effects without Hardy Weinberg assumptions reveals nonlinear dose response relationships and partial recessivity across proteins and complex traits in UK Biobank.

## Introduction

Genome-wide association studies (GWASs) are fundamental to understanding complex traits and diseases. Additive genetic association testing, in which the phenotype of interest is regressed on the number of copies of a biallelic genotype ([0, 1, 2]), is by far the most widely used approach in GWASs. This is because additive models perform remarkably well for common variants, even when alleles at a locus influence the trait in a manner that deviates from additivity^1–4^. This observation, coupled with the increasing scale of modern genetic sequencing datasets, has spurred the development of computationally efficient linear mixed effects model (LMM) algorithms that are primarily designed to test the explanatory power of additive genetic effects to trait variation. As a result, the deviation from additivity at a locus, known as the ‘dominance deviation’ or nonadditive contribution, is rarely tested in large-scale genetic analyses.

Interrogating nonadditive effects is particularly useful for understanding the continuous relationship between phenotypic expression and gene dosage. In classical Mendelian recessive disorders, such as cystic fibrosis, trait manifestation requires pathogenic *CFTR* variants that are either homozygous, where both gene copies harbor the same variant, or compound heterozygous (CH), where both copies harbor different variants, usually at distinct genetic locations within the same gene locus^5,6^. In other conditions, such as familial hypercholesterolemia (FH) caused by *LDLR* mutations, heterozygotes show moderately elevated LDL cholesterol while homozygous carriers present with higher levels, creating an additive relationship between functional allele count and clinical presentation. These examples illustrate different ‘dose-response’ relationships — how the number of mutated gene copies relates to disease severity. Understanding whether these relationships are linear or nonlinear is important because they inform the drug development process; for example, minimal restoration of CFTR function significantly improves cystic fibrosis symptoms^7,8^, while similar minimal restoration of LDLR function may only partially alleviate symptoms in homozygous carriers^9^.

In principle, deviations from additivity at a locus can be tested by performing a likelihood ratio test (LRT) on two nested models: a 2-degree-of-freedom (DF) model (containing, for example, an additive term ([0, 1, 2]) and a second dominance deviation term ([0, 1, 0] or [0, 0, 1]), and a simpler 1-DF model that contains only the additive term. However, it is currently not straightforward to implement the 2-DF model into efficient and scalable mixed-effects models. As a result, biobank-scale investigations of nonadditive effects have resorted to comparing *P*-values or effect sizes between additive ([0, 1, 2]) and recessive ([0, 0, 1]) encodings^10,11^ in 1-DF models. These encodings are correlated, which leads to ‘additive spillover’ and biased effect size estimates. Such bias can cause additive genetic associations to be incorrectly classified as recessive. When LRTs have been performed in the literature, they have typically been restricted to top loci where *P*-values from a recessive model are significant after multiple-testing correction^12^, potentially missing associations with genetic architectures that deviate from a purely recessive model, for example, when the underlying mode of inheritance is partially recessive. This also violates statistical independence between the initial discovery step and subsequent hypothesis testing, constituting a form of data-driven inference that, without proper correction procedures, leads to systematic inflation of Type I error rates.

For common variants (minor allele frequency (MAF) ≥ 0.01), an alternative solution has been proposed, whereby a specific ‘nonadditive’ allelic recoding imposes orthogonality with the additive encoding at a locus. The transformation [0, 1, 2] → [ − *p*/(1 − *p*), 1, − (1 − *p*)/*p*], where *p* represents the MAF, has been widely used to test for nonadditive effects in common variants^3,4,13^. This approach relies on Hardy-Weinberg equilibrium (HWE), making it unsuitable for rare variants, which often violate this assumption due to selection, recent mutation events with insufficient time to reach equilibrium in the population, or because observed genotype counts must be whole numbers, not the fractional counts predicted under HWE^14–16^. This limitation is particularly problematic as rare variants are expected to show the strongest deviations from additivity, partly because deleterious alleles with large effects are maintained at low frequencies by negative selection, and a consequence of rare deleterious variants typically acting in a recessive manner^17–19^. Detecting these effects faces an additional statistical challenge, as the number of rare variant homozygotes scales with the square of MAF, limiting power for detecting nonadditivity. Thus, the variants most likely to show large nonadditive effects are also the most difficult to study.

Here, we address these limitations by considering the orthogonal nonadditive allelic recoding of genotypes, which does not assume HWE, which has been largely overlooked for rare variant analysis in large-scale genetic datasets. Our framework parameterizes the relative genotype (wildtype, monoallelic and biallelic) proportions, instead of the Allele Frequency (AF), allowing for deviation from HWE. Using this encoding, we implement the resultant model in a fast and scalable framework for rare variant nonadditive analysis, which can be used with existing LMM tools, such as SAIGE or REGENIE. We apply the encoding to variant and set-based analysis, providing a means to perform rare-variant dominance burden analysis, i.e., a dominance deviation equivalent of the standard additive burden testing commonly carried out in rare-variant gene-based testing. We perform nonadditive and additive analysis of rare CH and homozygous variation (MAF  < 0.05) at the gene level to investigate both monotonic and non-monotonic dosage effects on 3,000 proteomic measurements and 55 quantitative traits in the UK Biobank (UKBB). We replicate observed nonadditive effects using the All of Us (AoU) cohort^20^. Through this approach, we partition orthogonal genetic effects locus-by-locus, revealing how changes in gene dosage produce both linear and nonlinear responses across molecular and common complex traits.

## Results

### Encoding nonadditivity at the gene level using rare deleterious variants

**Fig. 1:**
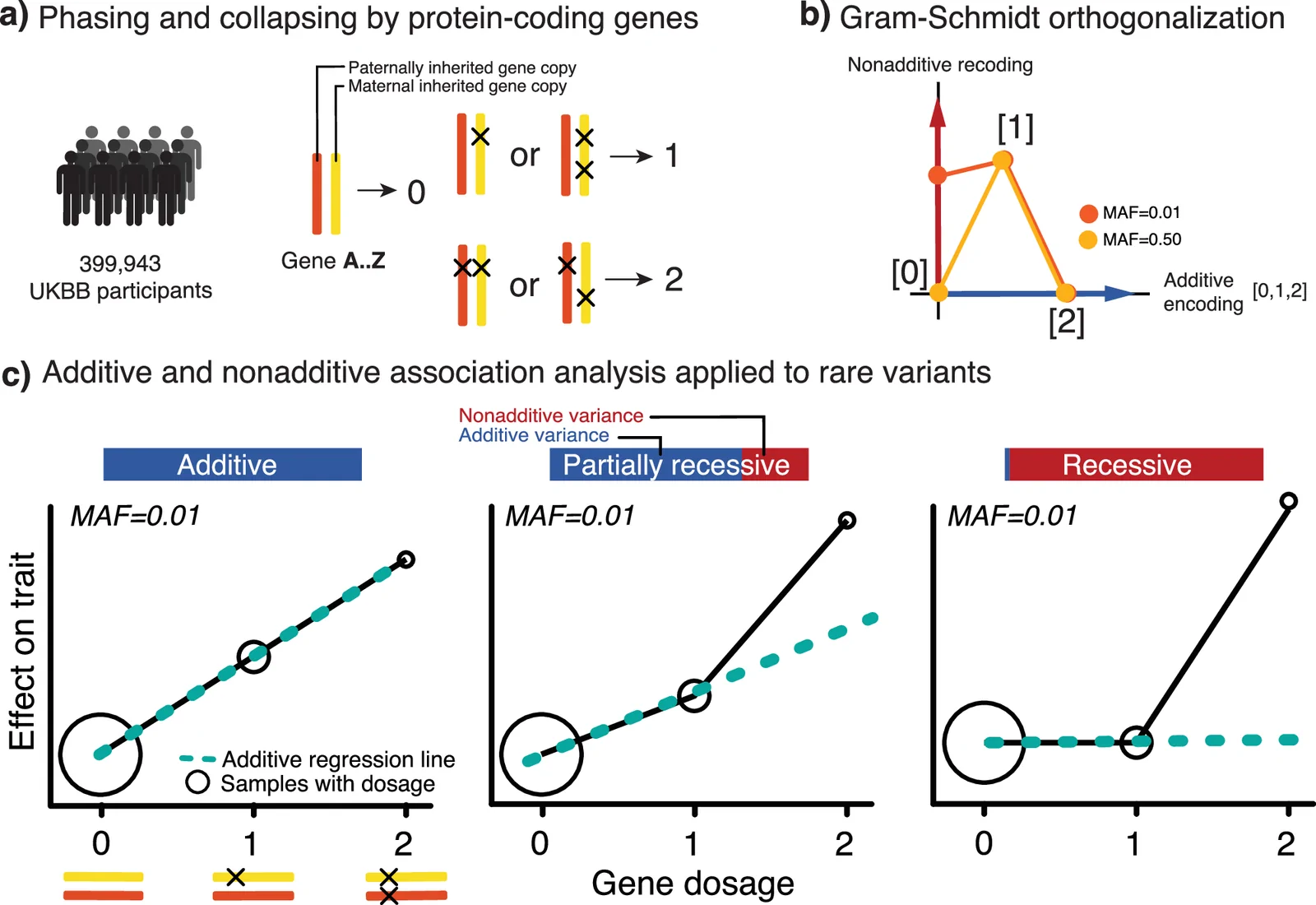
Investigating rare variant nonadditive effects. **a** We phase 399,943 European UK Biobank participants to distinguish between maternal (red) and paternal (yellow) gene copies. For each protein-coding gene, we count copies with deleterious allelic substitutions: 0 for no variants in either copy, 1 when either parental copy contains variant(s), and 2 when both copies contain variant(s). **b** We implement Gram-Schmidt orthogonalization to transform the standard additive encoding ([0,1,2] on the x-axis) into a nonadditive recoding (y-axis). This mathematical transformation creates representations that explicitly separate additive and nonadditive genetic effects. Here, the recoding is shown using MAF = 0.01 and 0.50. **c** The relationship between genotype and phenotype for three genetic architectures (Additive, partially recessive, and Mendelian recessive) at MAF = 0.01. The *x*-axis represents allelic substitutions (0, 1, 2), and the *y*-axis shows the phenotypic effect. White circles indicate genotype frequencies, with size proportional to frequency in the population. The dashed cyan line represents the fit of a linear additive model. The bar above each plot displays variance explained from an additive model (blue), and the residual variance explained is the nonadditive contribution (red). The genetic architectures, shown from left to right, are: (1) Additive, where the effect on the phenotype doubles with each allelic substitution; (2) Partially recessive, with some effect in heterozygotes but larger in homozygotes; and (3) Mendelian recessive, where the effect is only present in the homozygous alternate state. At MAF = 0.01, the additive model fails to capture the Mendelian recessive architecture effectively, with a large majority of variance (~ 98%) explained by the nonadditive model.

Since deviations from additivity can only be detected when biallelic carriers are present, we first expanded the number of identifiable biallelic carriers in UKBB to increase power for detecting such effects (Fig. 1a). We performed extensive quality control (Supplementary Figs. 1–4), and identified 18,992,659 high-quality exome sequencing (ES) variants in 399,943 individuals of European ancestry. Then, we performed statistical phasing using SHAPEIT5^21^ (“Methods”) to incorporate CH variation, which we have previously demonstrated increases power in biallelic association analysis^22^. Briefly, incorporating compound heterozygous variation expands the number of identifiable biallelic genotypes, thereby improving statistical power to detect recessive effects. We confirmed high phasing accuracy across the allele frequency spectrum using 96 trio-families (Supplementary Data 1) and restricted our subsequent analysis to confidently phased variants (PP ≥ 0.9).

We enriched our analysis for rare (MAF  < 0.05) variants with expected large effect sizes by restricting to two categories of predicted deleterious variation: high confidence loss-of-function (LoF) variants, and a combined category including both putative loss of function (pLoF) variants and damaging missense/protein altering variants (putatively deleterious missense, splice site, and low confidence protein truncating variants (PTVs)). Using phase information, we then collapsed variants at the gene level within each category to encode the number of likely inactivated haplotypes, which we refer to as pseudo-variants (Fig. 1a). This allows us to also analyze the effects of CH variation at the gene level, expanding the total number of identifiable biallelic carriers. Our collapsing approach yielded 39,511 homozygous and 8669 CH pLoF genotypes, representing an additional 21% biallelic genotypes compared to homozygous carriers alone (Supplementary Data 2). For the combined category, we identified 132,783 homozygous and 49,570 CH genotypes across 144,636 carriers (Supplementary Data 2). Simulations confirmed high concordance between expected and empirical CH (*R*^2^ = 0.90) and homozygous (*R*^2^ = 0.98) carrier counts (Supplementary Fig. 5).

We then imposed orthogonality between the additive and nonadditive encoding of pseudo-variants using Gram-Schmidt orthogonalization^23^ (Fig. 1b and Supplementary Notes 1–2):

1 $$[0,1,2]\to \frac{1}{\sqrt{har(a+r-{(a-r)}^{2})}}[-ha,2ar,-hr],$$

In this encoding (Equation (1)), *r*, *h*, and *a* represent the proportions of individuals with zero, one, and two haplotypes carrying one or more alternate alleles, respectively. This represents an alternate encoding where nonadditive effects can be tested in parallel with additive effects using LMMs (Fig. 1c). By parameterizing the observed genotype proportions, as opposed to MAF, we avoid the HWE dependency. This, in turn, enables testing of deviations from additivity across the full AF spectrum, including ultra-rare variants.

Using simulations, we demonstrate that this approach is well-calibrated under the null hypothesis with both SAIGE and REGENIE (“Methods”; Supplementary Figs. 6, 7) and reliably detects true signals across diverse genetic architectures (“Methods”; Supplementary Figs. 6, 7). To evaluate robustness to violations of HWE, we compared our method against standard HWE-dependent allelic recoding, using the Wright inbreeding model^24^ to simulate populations with increasing rates of consanguinity. Our approach maintained well-calibrated *P*-values regardless of deviation from HWE (*λ*_GC_ = 0.94 at *F* = 0.35), whereas standard recoding exhibited substantial inflation (*λ*_GC_ = 78.3 at *F* = 0.35; Supplementary Fig. 11).

We considered three allelic encodings for our analyses: the additive ([0, 1, 2]) and recessive ([0, 0, 1]) encodings, both widely used in the literature, and the nonadditive encoding from equation (1). Using SAIGE, we performed gene-level mixed-effects association analysis between pseudo-variants (CHs and homozygotes) and phenotypes of interest. All investigated gene-trait associations remained well-calibrated under the null (Supplementary Figs. 12–15), and we confirmed that our results were reproducible with REGENIE^25^ (Supplementary Fig. 14) and robust to phenotype transformation (Supplementary Fig. 14). For any putatively significant association (additive or nonadditive), we performed exhaustive conditional analysis, conditioning on nearby common variants (within 500 kb and MAF ≥ 0.01) that could potentially explain the signal (Methods; Supplementary Data 3–4).

Here, we analyze nonadditive effects using gene-level “pseudo-variants.” However, because haplotype estimation can be computationally prohibitive for large datasets, we also demonstrate compatibility with single-variant analyses and standard set-based association methods such as SAIGE-GENE+^26^, which do not require phased genotypes (Supplementary Note 3).

### Non-linear gene dosage effects are widespread across the plasma proteome

We first analyzed the 2906 protein abundance measurements across 42,217 Europeans from the plasma proteomic (Olink) panel in UKBB^27^. Protein expression serves as an ideal testing ground to identify nonadditive effects for several reasons: (1) proteins represent the products of gene activity and regulation, allowing us to examine nonadditive effects in a mechanistically interpretable context upstream of complex traits; (2) protein levels are highly heritable and are responsive to genetic variation^28^; and (3) while a previous study has examined rare variant effects on protein levels using separate additive ([0, 1, 2]) and recessive ([0, 0, 1]) encodings^29^, these approaches produce correlated *P*-values and fail to quantify deviations from additivity, a gap our framework directly addresses.

**Fig. 2:**
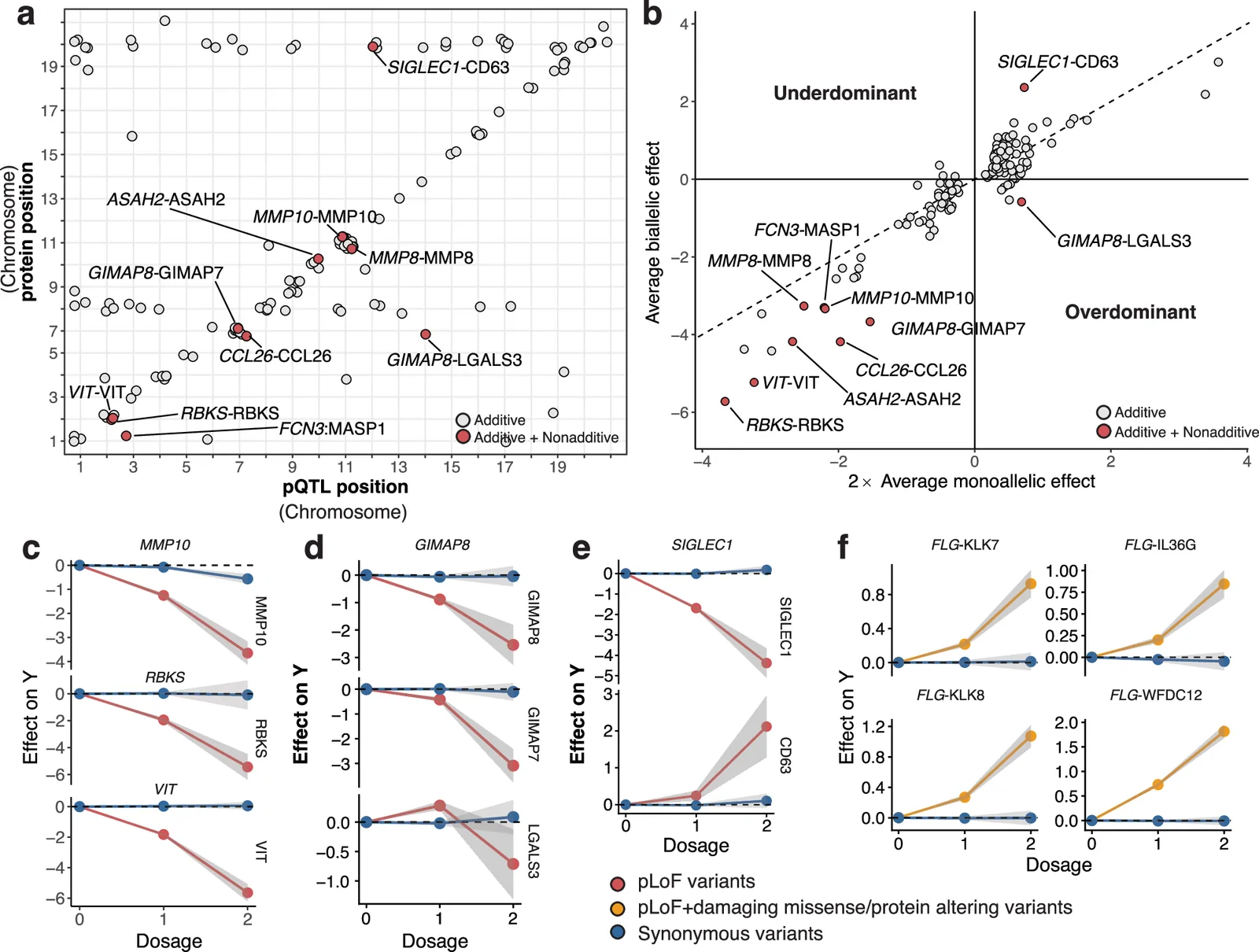
Nonadditive effects and dose-response relationships in proteomic traits. **a** Overview of significant (*P* < 1.17 × 10^−7^) additive protein quantitative trait loci showing additive-only (gray) and additive + nonadditive (red) effects across chromosomes. Significant nonadditive associations (*P* < 0.05/136) are labeled with gene:protein pairs. All *P*-values are two-sided, derived from score tests in a linear mixed model (SAIGE) with Bonferroni correction for multiple comparisons. Exact *P*-values for all labeled associations are provided in Supplementary Data 5 and 6. **b** Comparison of average biallelic effect versus 2 × average monoallelic effect. Significant nonadditive associations (red) appear in overdominant (lower right) or underdominant (upper left) quadrants. **c**–**f** Dose-response curves across genotype dosage (0 = wildtype, 1 = monoallelic, 2 = biallelic) comparing pLoF variants (red lines) with synonymous variants (green lines) for: (**c**) *MMP10*, *RBKS*, and *VIT* effects on their cognate proteins; (d) *GIMAP8* effects on GIMAP8, GIMAP7, and LGALS3; (**e**) *SIGLEC1* effects on SIGLEC1 and CD63; (**f**) *FLG* effects on four selected proteins using pLoF + damaging missense variants (yellow) versus synonymous variants (green). Gray shading represents 95% confidence intervals around the estimated marginal genetic effect (center line), derived from combined additive and nonadditive standard errors assuming orthogonality (“Methods”). All dose-response effects are displayed after correction for the influence of nearby common variants. Source data are provided as a Source Data file.

We performed gene-level mixed-effects association analysis between pseudo-variants (CH and homozygotes) and protein abundance across 146 genes with at least five biallelic pLoF carriers, analyzing a total of 399,442 gene-protein pairs. Gene-protein pairs that exhibit additive effects are enriched for monotonic nonadditive effects, because a trait following a Mendelian mode of inheritance is often still detectable in an additive model at higher MAFs (Supplementary Note 4). For this reason, we opted for a two-stage approach where we first identified 136 gene-protein pairs with significant additive effects (Bonferroni *P* < 1.17 × 10^−7^=$\frac{0.05}{2906\times 146}$), and then tested for nonadditive effects within this subset, finding 10/136 (7.4%) significant gene-protein pairs (Nonadditive *P* < 0.05/136, Fig. 2a, b).

In comparison, a direct search for nonadditivity across all pairs would have identified fewer associations (6 vs 10) due to the more stringent multiple testing penalty. However, the direct search would have captured the likely recessive *APOBEC1*-TMPRSS15 relationship (Nonadditive *P* = 5.53 × 10^−9^), which our two-stage approach missed because the pair lacked a genome-wide significant additive signal (Additive *P* = 0.002).

#### Evidence that one in three cognate gene-protein pairs exhibit nonlinear dose-response effects

Cognate protein levels are generally expected to scale linearly with the incremental acquisition of deleterious LoF variants on each gene copy^30^ (referred to here as ‘gene dosage’). This is because bona-fide LoF variants may trigger Nonsense Mediated Decay (NMD), leading to complete transcript degradation^31^. However, factors such as cis-regulatory feedback mechanisms can create nonlinear dependencies between gene dosage and protein abundance. In our analysis, across 399,442 gene-protein comparisons, we tested 24 cognate pairs with at least five biallelic carriers, of which 21 (87.5%) were significantly (Additive *P* < 1.17 × 10^−7^) associated with a reduction in protein abundance. Furthermore, 6/21 (28.6%) also exhibit significant nonadditive effects (Nonadditive *P* < 0.05/136), such as *MMP10*, *RBKS*, and *VIT* (Fig. 2c). Thus, our results suggest that approximately one in three cognate gene-protein pairs could exhibit nonlinear dose-response relationships, potentially indicative of distinct gene regulation.

Beyond cognate effects, we observed trans-acting nonadditive effects among 4/10 gene-protein pairs, where variants in one gene affected protein levels of unrelated genes. For example, while pLoF variants in *SIGLEC1* were additively associated with a reduction in cognate SIGLEC1 abundance, we also observed a nonadditive trans effect on CD63, which manifested as significantly higher protein abundance in biallelic *SIGLEC1* pLoF carriers compared to monoallelic carriers (Fig. 2e). The GIMAP family provided another example of nonadditive trans-acting effects (Fig. 2d). Here, we observed that *GIMAP8* pLoF variants were additively associated with GIMAP8 protein levels (Additive *P* = 8.34 × 10^−193^, nonadditive *P* = 0.04), and in addition exhibited a nonadditive effect on its paralog GIMAP7 (Additive *P* = 5.41 × 10^−160^, nonadditive *P* = 1.17 × 10^−10^). This suggests a threshold-dependent regulatory relationship between these proteins. GIMAP7 expression appears to be substantially enhanced only when GIMAP8 abundance falls below a critical threshold, as observed in carriers of biallelic LoF variants.

#### Evidence of overdominance in *GIMAP8* pLoF carriers

Further analysis of *GIMAP8* pLoF variants revealed a complex relationship with LGALS3 protein levels, characterized by both significant additive and nonadditive effects (Fig. 2d). We observed a significant additive relationship between *GIMAP8* pLoF variants and increased abundance of LGALS3 (Additive *P* = 1.78 × 10^−35^). Unexpectedly, we also detected significant nonadditive effects acting in the opposite direction (*P* = 2.69 × 10^−5^, *P* < 0.05/136). The combination of these opposing effects manifested as apparent overdominance, with heterozygotes exhibiting elevated LGALS3 levels compared to both wild-type and homozygous individuals. To our knowledge, this represents a rare example of apparent overdominance affecting protein levels in human genetic studies, a phenomenon that has primarily been documented for fitness-related traits such as malaria protection in sickle cell heterozygotes^32^.

Given this finding, we performed extensive validation. First, we tested whether the signal could be explained by *GIMAP8* pLoF variants being in linkage disequilibrium (LD) with nearby common variants modulating gene expression. Conditional analysis on nearby variants (500 kb upstream/downstream, MAF  > 0.01) did not alter the significant additive signal (conditional additive *P* = 2.64 × 10^−12^; Supplementary Data 3), suggesting the effect was driven by the pLoF variants themselves rather than linked regulatory variants. Second, we found no significant effects in synonymous variant carriers (Additive and nonadditive *P* > 0.05), supporting a functional role for protein-altering variants. Third, an independent study^29^ replicated the additive component of this signal, confirming that *GIMAP8* variants are associated with increased LGALS3 abundance. While we observed high global concordance with these results (*R* > 0.99; Supplementary Fig. 16), their reliance on a strictly linear model failed to capture the significant nonadditive deviation. Although replication of the nonadditive components in independent cohorts is essential to confirm this finding, this observation demonstrates how our orthogonal framework can uncover complex genetic architectures that deviate from simple dose-response relationships and would be missed by conventional additive or recessive models.

#### Differential dose-response effects across the *FLG* trans-regulatory network

While pLoF variants lead to transcript degradation via NMD, missense and splicing variants can alter protein levels through multiple mechanisms, including altered mRNA processing^33^, altered translation efficiency^34^, and post-translational modifications^35^, potentially revealing regulatory feedback loops. To investigate this, we included damaging missense/protein altering variants in addition to pLoF variants, thus expanding the number of testable genes from 146 to 588 with at least five biallelic carriers (Supplementary Data 6). To justify their inclusion, we confirmed through comparative analysis that this variant class showed greater protein abundance reductions than synonymous carriers in additive association tests (Supplementary Data 7 and Supplementary Fig. 17).

Across 588 genes, we identified 417 significant gene-protein associations (Bonferroni $P < 2.92\times 1{0}^{-8}=\frac{0.05}{588\times 2 906}$) under the additive model, of which 23 exhibited significant nonadditive effects (*P* < 0.05/417 = 1.20 × 10^−4^). While all 10 originally identified pLoF associations remained significant when using the expanded annotation mask, we uncovered 13 additional nonadditive relationships driven by two genes: *KLK14* (1 association) and *FLG* (12 associations). The latter encodes filaggrin, a key skin barrier protein, and provided a notable example of pleiotropic effects (Fig. 2f and Supplementary Fig. 18). While we could not measure FLG abundance itself, variants in this gene were additively associated with 41 proteins (*P* < 2.92 × 10^−8^), with 12 showing significant nonadditive effects (*P* < 0.05/417). The presence of both additive and nonadditive effects suggests that some protein networks appear to respond proportionally to *FLG* dosage, while others demonstrate ’switch-like’ behavior, increasing disproportionately when *FLG* levels fall below critical thresholds (Supplementary Fig. 18).

### Nonadditive effects contribute to complex trait variation

**Fig. 3:**
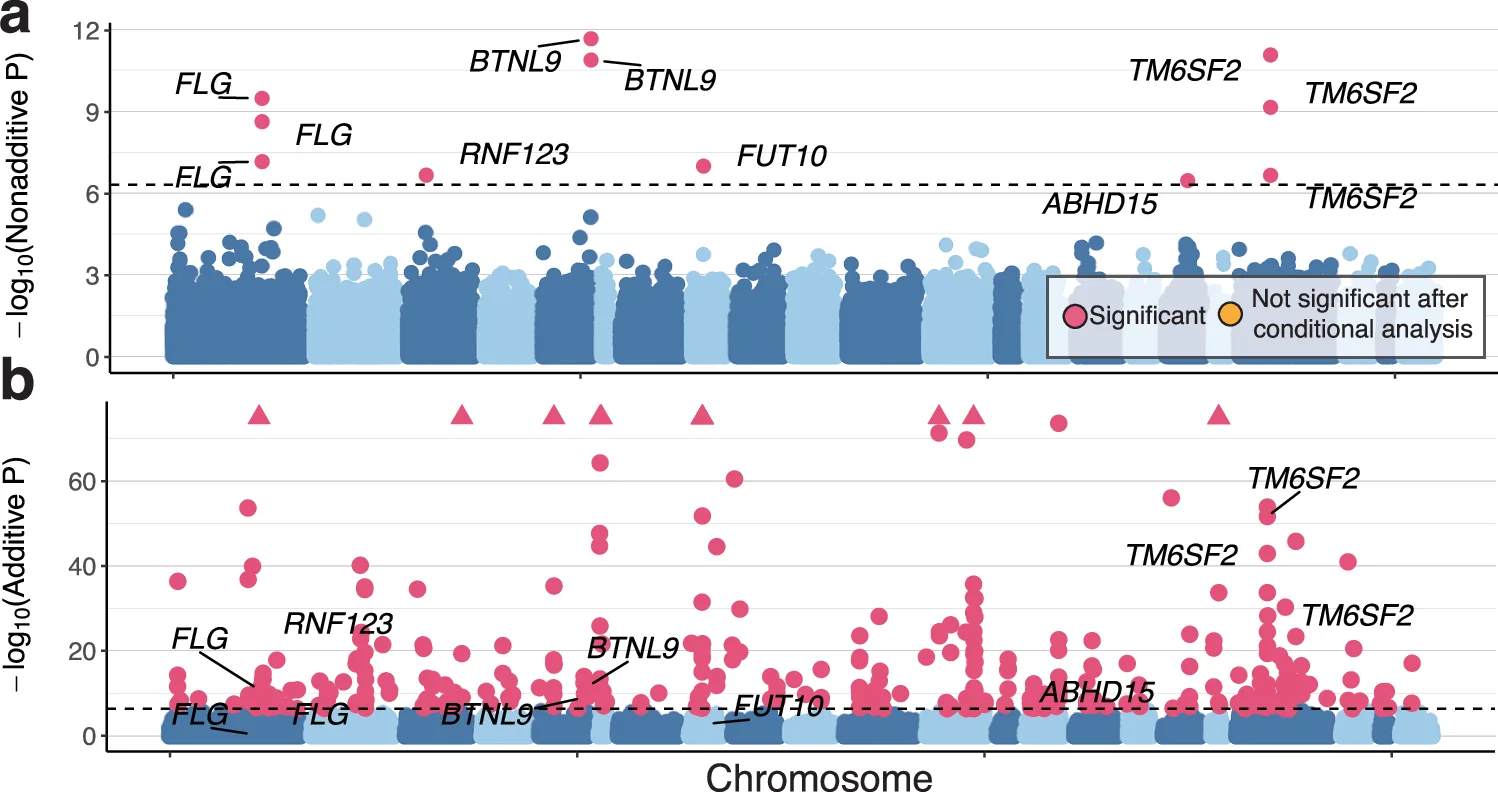
Manhattan plots comparing additive against nonadditive genetic effects across medically relevant traits. We performed genome-wide association analyses using different genotype encodings: (**a**) our nonadditive allelic versus (**b**) the standard additive encoding [0, 1, 2]. Each point represents a gene-trait association, with $-{\log }_{10}$(P) plotted against chromosomal position. Alternating light-blue and dark-blue colors distinguish adjacent chromosomes. All gene-traits significant in the nonadditive model have been labeled. The dashed horizontal lines represent Bonferroni-corrected significance thresholds (Nonadditive *P* < 4.65 × 10^−7^). All *P*-values are two-sided, derived from score tests in a linear mixed model (SAIGE). To determine if these signals were driven by nearby common variants, we performed conditional analyses on all significant associations. Orange labels indicate gene-trait associations that lost significance in the nonadditive model after conditioning on nearby common variants. Triangles indicate that the *P*-value has been truncated for visualization purposes (*P* < 10^−75^). Source data are provided as a Source Data file.

While the proteomic analysis provided mechanistic insights into gene regulation, the relationship between genetic variation and disease often involves physiological processes that extend beyond protein abundance. Therefore, we conducted an exhaustive search for nonadditive effects across 395,517 individuals of European ancestry, analyzing 55 heritable quantitative traits (measuring body dimensions, blood pressure, pulmonary function, bone density, blood cell parameters, and serum biomarkers; Supplementary Data 8). For traits with significant nonadditive effects, we sought to replicate the associations using homozygous genotypes from AoU (Supplementary Data 9). Unlike the proteomic analysis, we searched for nonadditive effects directly, regardless of whether additive effects were present, as purely recessive effects driven by ultra-rare variation may lack detectable additive signals (Fig. 1c). We prioritized 1952 genes with at least five biallelic pLoF and/or damaging missense/protein altering carriers, applying a conservative Bonferroni correction (Nonadditive $P < 4.65\times 1{0}^{-7}=\frac{0.05}{1952\times 55}$), and identified 11 significant nonadditive gene-trait associations across 6 genes and 10 traits (Supplementary Data 10 and Fig. 3a).

#### Rare recessive variants in *FUT10* are associated with reduced FEV1 and COPD risk

Our analysis identified *FUT10* as a gene exhibiting recessive effects on respiratory function. Multiple fucosyltransferases are known to influence airway obstruction, with *FUT2* shown to promote epithelial fucosylation and exacerbate airway inflammation^36,37^. Common variants in *FUT10* have been previously implicated in interstitial lung disease^38^, suggesting this gene plays a broader role in pulmonary function. We found that rare variants in this gene exhibit a recessive mode of inheritance with respect to forced expiratory volume in the first second (FEV1). Biallelic carriers exhibited significantly reduced FEV1 (Recessive *P* = 1.08 × 10^−8^, nonadditive *P* = 8.19 × 10^−8^), whereas heterozygous carriers showed a substantially attenuated effect that did not reach genome-wide significance (Additive *P* = 0.004). This recessive effect appears specific to functionally deleterious variants, as synonymous *FUT10* variant carriers showed no association (Additive *P* = 0.48, nonadditive *P* = 0.61). Furthermore, no common variants within 500 kb showed significant association with FEV1 (minimum *P* = 1.31 × 10^−3^), suggesting the signal is not driven by LD with nearby common regulatory variation.

Reduced FEV1 is a diagnostic criterion for chronic obstructive pulmonary disease (COPD), indicating airflow limitation characteristic of obstructive pulmonary disease. We therefore examined whether *FUT10* variants might contribute to clinical disease. Biallelic *FUT10* variant carriers had a nominally increased rate of COPD diagnosis in both UKBB (SAIGE recessive *P* = 0.017; additive *P* = 0.86) and AoU (SAIGE recessive *P* = 0.017; additive *P* = 0.75). We were unable to directly replicate the primary outcome (FEV1) in AoU, due to a paucity of FEV1 measurements (*n* = 513). Collectively, our results provide evidence that *FUT10* is a recessive genetic risk factor for obstructive pulmonary disease.

#### Additive and nonadditive analysis reconciles modes of inheritance

Among the significant nonadditive gene-trait associations, we observed multiple relationships with lipid traits. For example, variants in *BTNL9* were associated with increased Apolipoprotein A (Additive *P* = 2.57 × 10^−12^, nonadditive *P* = 1.99 × 10^−12^) and decreased high-density lipoprotein (HDL) cholesterol (Additive *P* = 6.37 × 10^−10^, nonadditive *P* = 1.20 × 10^−11^), a lipid profile linked to adverse cardiovascular outcomes^39,40^. We successfully replicated the HDL cholesterol finding in the AoU Biobank (Additive *P* = 1.23 × 10^−7^, nonadditive *P* = 3.17 × 10^−5^), though Apolipoprotein A measurements were insufficient in this validation cohort (n=2,035). Interestingly, conditioning on nearby common intronic variants (rs138692142 for Apolipoprotein A; rs147577717 for HDL cholesterol; MAF  > 0.01, within 500 kb) abrogated the additive signals entirely for Apolipoprotein A and HDL cholesterol (*P* = 0.10 and *P* = 0.26, respectively) and partially attenuated the nonadditive signals (*P* = 5.68 × 10^−3^ and *P* = 1.06 × 10^−5^). These intronic variants were not included in our rare variant burden (which was restricted to pLoF/missense variants with MAF  < 0.05); rather, we conditioned on them to assess whether our rare variant signals were independent of nearby common variation. The attenuation of the additive signals but persistence of the nonadditive signals after conditioning suggests that the rare variant effects are partially tagged by common variants under an additive model, but the nonadditive component, driven by homozygous rare variant carriers, remains detectable.

Collectively, these observations suggest a predominantly Mendelian recessive mode of inheritance for deleterious variants in *BTNL9* and HDL cholesterol, which would likely have been missed without joint conditional and nonadditive analysis. In fact, these findings extend previous work identifying a common stop-gain variant (rs200884524, MAF = 0.23) in *BTNL9* associated with atherogenic lipid profiles in Samoans^41^. While this study detected the association using an additive model, our analysis suggests this was likely due to the higher AF of this variant in the Samoan population, providing sufficient homozygous individuals to detect effects that are primarily manifested in homozygous carriers, effectively masking the underlying recessive architecture.

We also identified nonadditive pleiotropic effects for *TM6SF2* across multiple lipid traits: Apolipoprotein B (Additive *P* = 1.9 × 10^−34^, nonadditive *P* = 8.01 × 10^−12^), low-density lipoprotein (LDL) cholesterol (Additive *P* = 1.14 × 10^−43^, nonadditive *P* = 6.75 × 10^−10^), and total cholesterol (Additive *P* = 2.21 × 10^−52^, nonadditive *P* = 2.12 × 10^−7^). We observed no significant association among synonymous variants in this gene (all additive and nonadditive *P* > 0.05, Supplementary Data 10), supporting a functional effect of the nonsynonymous variants. In the AoU Biobank, we observed only the additive component for total cholesterol (Additive *P* = 1.15 × 10^−12^; nonadditive *P* = 0.76), likely due to insufficient biallelic carriers (*N* < 20). This discrepancy mirrors broader inconsistency in the literature, where *TM6SF2* associations with lipid traits have been reported to follow both additive^42^ and recessive^11^ modes of inheritance.

Our framework reconciles these conflicting reports. Conditional analysis attenuated the additive signal for Apolipoprotein B (*P* = 1.12 × 10^−4^), LDL cholesterol (*P* = 4.75 × 10^−6^), and to a lesser extent, total cholesterol (*P* = 2.52 × 10^−8^), while the nonadditive signals remained significant. The persistence of significant effects in both the additive and nonadditive components suggests a partially recessive mode of inheritance for *TM6SF2* and lipid traits. While both strictly additive ([0, 1, 2]) and recessive ([0, 0, 1]) models may detect these associations depending on cohort composition and statistical power, our framework partitions the additive and nonadditive genetic contributions, revealing that the impact of *TM6SF2* variation on lipid metabolism is more complex than previously appreciated. This partial recessivity likely varies in detectability across populations depending on the frequency of homozygous carriers, explaining why previous studies (and our replication cohort) have identified this gene through either additive or recessive models.

#### Characterization of *FLG* respiratory effects reveals recessive threshold mechanisms

**Fig. 4:**
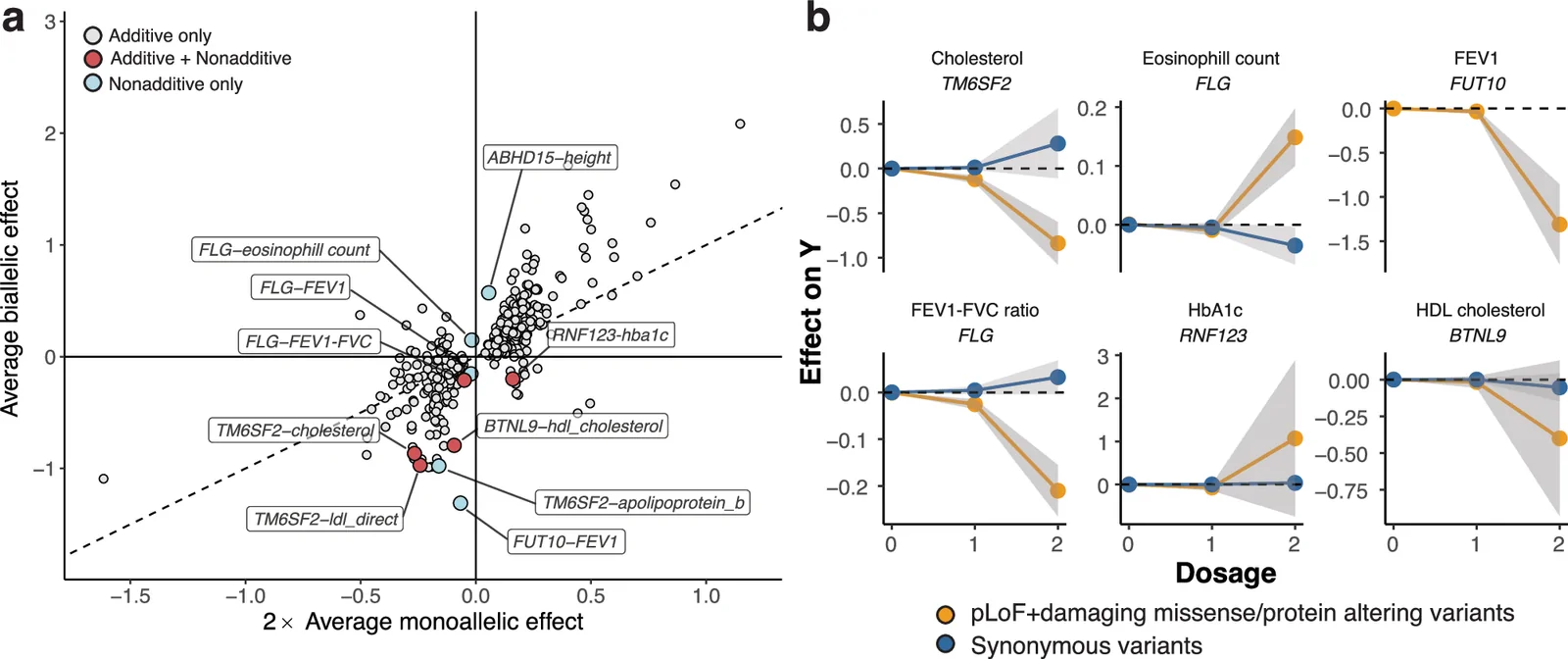
Genetic architecture of common complex traits. **a** Comparison of average biallelic effect versus 2 × average monoallelic effect for significant gene-trait associations. Points along the diagonal dashed line indicate additive relationships. Associations are colored by their genetic architecture: additive only (gray), nonadditive only (blue), or both additive and nonadditive effects (red). The gene-trait pairs discussed in the main text are labeled (Supplementary Data 10). **b** Dose-response relationships across genotype dosage (0 = wildtype, 1 = monoallelic, 2 = biallelic) for six significant associations after conditioning on nearby common variants. Each panel compares functional variants (pLoF + damaging missense/protein-altering, yellow) with synonymous control variants (green). Note that some gene-trait associations are highly attenuated upon conditioning on nearby common variants, such as *RNF123*-HbA1c. Gray shading represents 95% confidence intervals around the estimated marginal genetic effect (center line), derived from combined additive and nonadditive standard errors assuming orthogonality (“Methods”). Note: FEV1-*FUT10* synonymous dose-response effects are not shown due to the lack of biallelic synonymous carriers (*n *= 1). Source data are provided as a Source Data file.

While previous studies have established associations between *FLG* mutations and asthma risk^22^, the precise inheritance patterns for quantitative respiratory measures and underlying inflammatory mechanisms have remained unclear. Our orthogonal framework enables partitioning of additive versus nonadditive contributions to respiratory phenotypes. Building on known *FLG*-asthma associations, we demonstrate that biallelic *FLG* variants exhibit strictly recessive, ‘switch-like’ effects across quantitative respiratory measures: biallelic carriers exhibited significant reductions in FEV1 (Nonadditive *P* = 2.25 × 10^−9^) and FEV1-Forced Vital Capacity (FVC) ratio (Nonadditive *P* = 6.60 × 10^−8^), while heterozygous carriers maintained lung function statistically indistinguishable from wildtype individuals (Fig. 4b).

Most notably, we identified an inflammatory signature in biallelic carriers: marked elevation in eosinophil counts (Nonadditive *P* = 3.14 × 10^−10^) absent in heterozygous carriers. We were unable to replicate the association with eosinophils in AoU (Nonadditive *P* = 0.18), likely due to limited biallelic carriers in AoU. While UKBB included 476 homozygotes and 1129 CH carriers, AoU contained only 66 homozygous carriers and lacked phased data to identify CH genotypes (Supplementary Data 9; see Supplementary Note 3). This selective eosinophilic response suggests that complete loss of FLG function triggers distinct inflammatory cascades with a clear functional threshold, mechanisms that remain dormant with single allele loss. Collectively, these findings suggest that biallelic *FLG* carriers experience a subtype of asthma characterized by both airflow limitation and eosinophilic inflammation, while heterozygous and wildtype individuals remain unaffected.

#### Standard recessive models fail to accurately characterize modes of inheritance

Current methods for identifying recessive effects typically compare results from separate additive and recessive models^10,11^. We found that these approaches have critical limitations in both detecting and classifying true recessive effects. In our investigation, analyzing additive ([0, 1, 2]) or recessive ([0, 0, 1]) models independently would have led to 373 and 22 significant gene-traits, respectively (Fig. 3b and Supplementary Fig. 19). However, of the 22 gene-traits discovered in the recessive model, 8 (36%) exhibited no significant nonadditive contribution using our encoding (Nonadditive *P* > 0.05), indicating these are likely false-positive recessive associations. Some have proposed a head-to-head comparison of additive and recessive *P*-values or effect sizes^10,11^, assuming that more significant recessive *P*-values indicate genuine recessive gene-trait relationships. However, when we performed such a comparison, we identified only 7 putatively recessive gene-traits (Supplementary Data 10). In summary, using conventional testing, we would either have missed 4 of 11 (36%) associations with a significant nonadditive contribution or incorrectly considered 8 of 22 (36%) associations as recessive.

### Joint modeling improves power for partially recessive traits

**Fig. 5:**
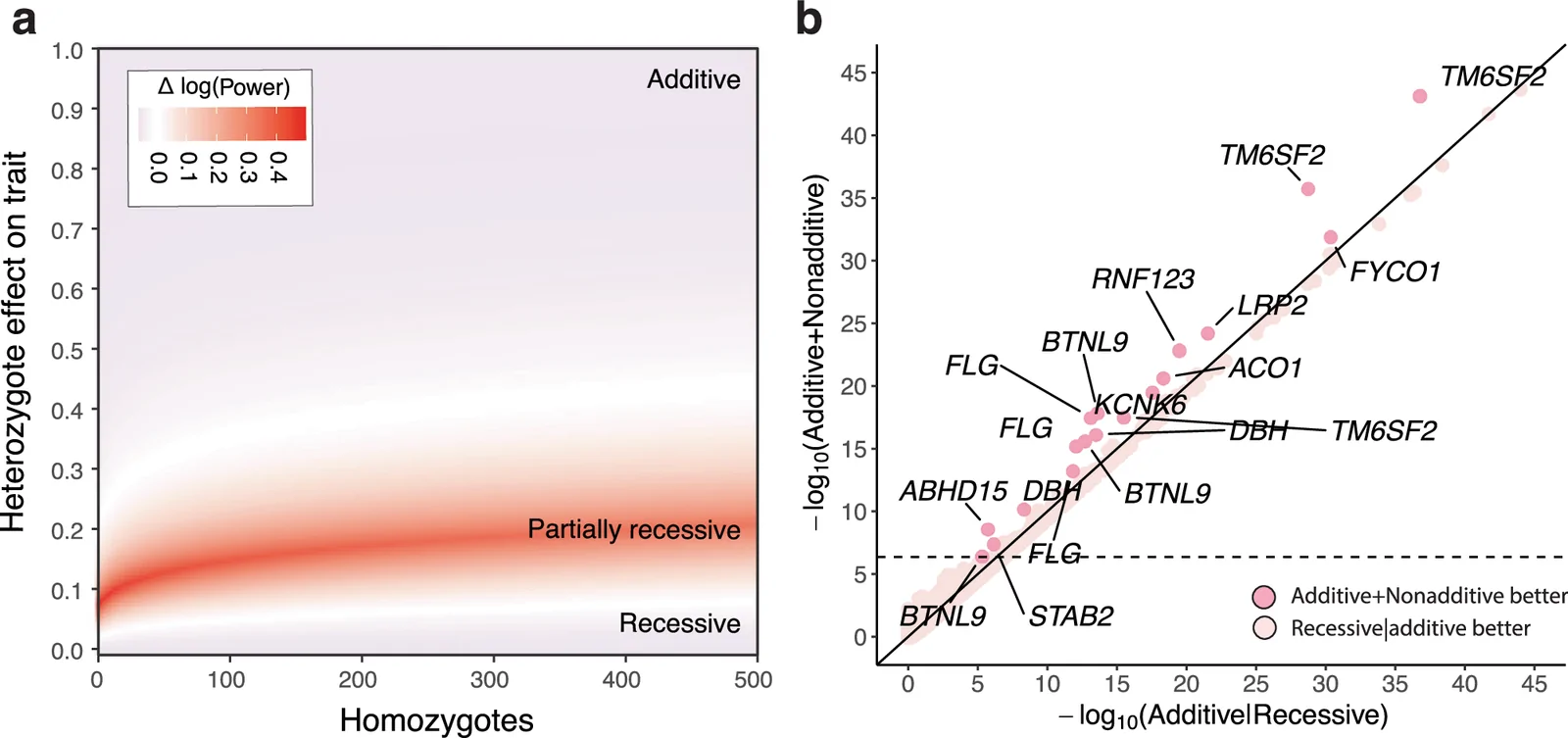
Partially recessive gene-traits have improved power for discovery. **a** Heatmap showing the relative power of a 2-DF joint model compared to taking the minimum *P*-value from separate additive and recessive models. The x-axis represents the number of homozygous carriers, while the y-axis represents the relative effect in heterozygotes (0 = no effect [strictly recessive], 1 = full effect [strictly additive]). Color intensity indicates the log-difference in power, with red regions showing where the 2-DF model substantially outperforms alternative approaches. Power was calculated using an analytical framework (“Methods”) and does not represent empirical test results. The 2-DF model demonstrates its greatest advantage in the partially recessive region, where heterozygotes have some intermediate effect on the trait. **b** Empirical comparison of statistical significance between the joint 2-DF model (y-axis) and the Cauchy-combined *P*-values from separate additive and recessive tests (x-axis) across gene-trait associations. Each point represents a gene-trait pair, with points above the diagonal line indicating greater significance in the joint model. Gene-trait associations with at least 10-fold greater significance in the joint model are labeled. The dashed line indicates the genome-wide significance threshold (*P* < 4.45 × 10^−7^). All *P*-values are two-sided, derived from score tests in a linear mixed model (SAIGE) with Bonferroni correction for multiple comparisons. Exact *P*-values for all significant gene-trait associations, including joint 2-DF and Cauchy-combined *P*-values, are provided in Supplementary Data 11. Source data are provided as a Source Data file.

We hypothesized that a model incorporating both additive and nonadditive components simultaneously could improve overall power for association testing. Testing both components in a 2-DF framework incurs a statistical penalty relative to a 1-DF model due to estimating an additional parameter. However, whether capturing both effects could outweigh this penalty remained unclear and under which modes of inheritance this would occur. To investigate this, we compared the power of a 2-DF model against standard additive and recessive models across varying genetic architectures. Using an analytical framework (Methods), we found that 2-DF models are better powered for specific combinations of heterozygote and homozygous carrier effects (Fig. 5a). Specifically, we observed that the advantage of a 2-DF model is greatest when there is some effect in heterozygotes, but disproportionately larger effects in homozygotes, i.e., when the mode of inheritance is partially recessive. In other words, the 2-DF model becomes suboptimal as inheritance patterns approach either purely recessive or additive.

Given this insight, we empirically tested whether the 2-DF model enhanced statistical power compared to explicitly recessive or additive models in a subset of 339,040 unrelated individuals in UKBB. To implement this approach, we leveraged the orthogonality between our encodings, fitting separate fixed-effects linear models for the additive and nonadditive components, and then combining their *χ*^2^ test statistics to obtain a joint 2-DF test of significance (“Methods”). We confirmed calibration by analyzing 50 randomly simulated null traits, finding no systematic bias (median *λ*_GC_ = 1.009). We then analyzed the same 55 quantitative traits across 2039 genes with at least 5 biallelic carriers, testing a total of 10,996 gene-trait combinations. For each gene-trait pair, we compared the statistical significance achieved using the 2-DF model against a Cauchy-combined (CCT) *P*-value that integrated evidence from both single-component models ([0, 1, 2] and [0, 0, 1]) while maintaining well-calibrated *P*-values^43,44^ (“Methods”).

Across the 55 quantitative traits, we identified 265 significant ($P < \frac{0.05}{2039\times 55}=4.45\times 1{0}^{-7}$) gene-trait associations using our joint model, compared to 277 using the CCT approach (Fig. 5b). Among the 265 gene-traits identified, 121 (45.6%) were more strongly associated in the joint model. Of these, 44/121 (36.4%) exhibited nominally significant nonadditive and additive effects (both additive and nonadditive *P* < 0.05), suggesting that the joint model achieves higher power by modeling both contributions simultaneously. Moreover, using the joint 2-DF model, we identified five gene-trait associations that would otherwise have been missed at genome-wide significance using the CCT model. These included *ABHD15* with sitting height (joint *P* = 2.42 × 10^−9^ versus Cauchy *P* = 1.85 × 10^−6^), *MLXIPL* with alanine aminotransferase, and *STAB2* with IGF1 (joint *P* = 3.74 × 10^−8^ versus Cauchy *P* = 7.03 × 10^−7^). Among the 61 gene-traits, we also found examples of our 2-DF model achieving substantially higher significance levels. For example, the *TM6SF2*-LDL cholesterol association, which we previously established was likely under a partially recessive mode of inheritance, was approximately seven orders of magnitude more significant under the 2-DF framework (joint *P* = 7.37 × 10^−44^ versus CCT *P* = 1.76 × 10^−37^). These examples demonstrate the enhanced discovery potential of the joint modeling approach for traits with a partially recessive mode of inheritance.

## Discussion

We implemented an allelic recoding scheme that captures the nonadditive component of heritability, enabling efficient analysis of rare variant nonadditive effects in quantitative traits. Unlike previous methods that depend on HWE assumptions^3,4,13^, this approach is specifically designed for rare variants where HWE is frequently violated due to selection pressure, recent mutations, or constraints from integer genotype counts^14^. This recoding integrates seamlessly with existing LMM tools such as SAIGE and REGENIE, enabling analyses at a biobank scale. By applying our approach to proteomic measurements and complex traits in the UKBB, we successfully partitioned genetic effects into their additive and nonadditive components and identified nonadditive gene-trait associations. We demonstrate that relying on separate additive ([0, 1, 2]) and recessive ([0, 0, 1]) models fails to reliably distinguish the true mode of inheritance, highlighting the importance of explicitly testing the nonadditive contribution. Finally, we show that these nonadditive effects can be effectively investigated using a simple 2-DF model that combines additive and nonadditive components, improving power for discovering gene-trait associations with partially recessive inheritance.

Broadly, our results suggest that many gene dose-response relationships are more complex than previously appreciated. Our findings align with established protein dosage sensitivity models, where certain cellular pathways exhibit buffering mechanisms to maintain homeostasis^45–47^. In our proteomic analysis, approximately one-third of testable cognate gene-protein pairs exhibited nonlinear dose responses. Although based on a limited number of testable pairs, this suggests at least two distinct regulatory paradigms governing dose-response relationships. Linear relationships, characterized by proportional decreases in protein abundance with each LoF allele, could reflect efficient NMD that clears mutant transcripts from both alleles equally. In contrast, nonlinear patterns may reflect compensatory regulatory networks, including feedback inhibition release, activation of alternative biosynthetic pathways, or cis-regulatory feedback mechanisms that only engage when protein levels fall below critical functional thresholds.

Beyond mechanistic insights, we demonstrate that dose-response relationships at individual genes can be characterized using large-scale genetic datasets within LMM frameworks. This has direct implications for drug development: understanding whether a gene exhibits linear or threshold-dependent effects on disease phenotypes informs how much target modulation is required for therapeutic benefit. For instance, targets with recessive architectures may require only partial functional restoration, whereas those with additive effects may necessitate proportional correction. These insights are critical for optimizing dosing strategies and defining therapeutic windows.

The true prevalence of biological nonadditivity (e.g., recessive inheritance) is likely higher than our observed rate. For rare variants with recessive effects, the phenotypic signal is confined to rare homozygotes, and the additive model captures almost none of the genetic variance because the abundant heterozygotes are phenotypically neutral. Paradoxically, while such variants contribute an almost entirely nonadditive signal as a proportion of genetic variance, the absolute variance they generate is vanishingly small (proportional to *p*^2^), rendering them undetectable in exome-wide scans unless effect sizes are exceptionally large^2^. Our estimate, therefore, captures only cases where allele frequencies or effect sizes are sufficient to produce a statistically detectable deviation from additivity. Conversely, as biobanks continue to grow, they will increasingly detect statistically significant yet minor departures from linearity that may lack biological relevance, a challenge necessitating careful effect size thresholds alongside significance testing.

Our study has important limitations. First, we aggregated variants across genes using phased genotypes to perform a gene-based test of nonadditive effects. While this increases our power to detect nonadditive gene-trait associations, it may influence the inferred dose-response relationship, particularly when multiple variants with different effects segregate within the same gene. Second, while the approach outlined here effectively partitions genetic contributions in continuous traits, extending it to binary traits presents additional challenges. Because logistic regression introduces non-linearity between genotypes, phenotypes and covariates, it is difficult to define a general allelic transformation that preserves independence between the additive and nonadditive components when testing binary traits. Moreover, standard logistic regression can result in inflated type 1 errors when unbalanced case-control traits are tested. Addressing these challenges will require developing unified frameworks for testing nonadditive effects in binary traits.

In conclusion, our framework addresses a critical gap in the analysis of large-scale genetic datasets by enabling efficient testing of nonadditive effects. Through application to proteomic and complex trait data, with replication in an independent cohort, we have demonstrated that rare variant nonadditive genetic effects are detectable and contribute meaningfully to disease risk and biological processes. By developing methods to systematically identify and quantify these effects, we provide a more complete picture of genetic architecture across molecular and physiological traits.

## Methods

Further information on research design is available in the Nature Portfolio Reporting Summary linked to this article.

### Ethics

This study complies with all relevant ethical regulations. The UK Biobank has approval from the North West Multi-center Research Ethics Committee (MREC) as a Research Tissue Bank (approval 11/NW/0382). All UK Biobank participants provided written informed consent. This research was conducted under UK Biobank Application Number 11867. The All of Us Research Program is approved by the All of Us Institutional Review Board. All of Us participants provided informed consent. The study was conducted in accordance with the criteria set by the Declaration of Helsinki.

### QC of UK Biobank 450k exome release

#### Summary of exome sequencing quality control

For the 450 k UKBB ES data, we performed a series of initial hard filters on the variant level, on the sample level, and on the genotype level. We confirmed genetic sex with self-reported sex, and restricted our analysis to individuals that are confidently classified into broad ancestry groups (European (EUR), East Asian (EAS), South Asian (SAS) and African (AFR)). Following this, we applied a second round of final hard filtering based on the resultant samples and variants. We used Hail 0.2^48^ and PLINK 1.9^49^ coupled with bash, C, Python, and R scripts, to carry out all quality control (QC).

#### Initial hard genotype-level filters for exome sequencing

Multi-allelic variants were split into biallelic variants using split_multiallelic with hail, and insertions and deletions (indel) alignments were adjusted to be left-aligned^50^. The following criteria were used to designate genotype calls as missing:

1. Genotype quality (GQ) ≤ 20
2. Total Sequencing Depth (DP) ≤ 10
3. Phred-scaled likelihood (PL) of the genotypes  < 20
4. For heterozygous calls:
  - (AD_ref_ + AD_alt_)/DP < 0.8
  - (AD_alt_/DP) < 0.2
5. Homozygous indel calls: (AD_alt_/DP) < 0.8

where AD_ref_ and AD_alt_ refer to the allele depth supporting the reference and the alternate allele, respectively.

#### Initial variant level filters

After genotype-level filtering, we performed an initial variant-level filter based on the following criteria. Specifically, we discarded any variants that were:

1. not in the official exome capture target regions (See UKBB field 3803), padded with 50bp on either side.
2. located within a low complexity region (LCR)^51^.
3. invariant, i.e., minor allele count (MAC) equal to 0 after genotype quality control.

A total of 20,737,653 variants were left after the initial genotype-level filter and variant-level filter.

#### Imputing sex

To validate participant sex and compute principal components (PCs), we extracted high-quality common variants (MAF  > 0.01, call rate  > 0.98) and performed LD pruning to obtain pseudo-independent single-nucleotide polymorphisms (SNPs) using --indep 50 5 2 in PLINK 1.9. Discrepancies between reported and genotypic sex may indicate sample swaps. We calculated the *F*-statistic for each sample using non-pseudo autosomal regions on chromosome X and excluded samples where reported sex conflicted with genetic data (Supplementary Fig. 1). Specifically, we removed samples meeting any of these criteria:

- Missing sex information in phenotype files
- *F*-statistic  > 0.6 with female sex in phenotype file
- *F*-statistic  < 0.6 with male sex in phenotype file
- *F*-statistic  > 0.6 with  < 100 Y chromosome calls

#### Defining ancestry in samples

We focused our analysis on a set of samples with a genetically well-mixed population to ensure sufficient case-control for a wide range of traits. This was accomplished through a series of principal component analysis (PCA) steps. Initially, we conducted PCA on the 1000 Genomes (1KGP) cohort, excluding related individuals, using LD-pruned autosomal variants. Subsequently, we projected the UKBB while accounting for shrinkage bias. To assign ancestry, we employed a random forest (RF) classifier trained on 1KGP super-population labels. This classifier was used to predict the super-population of each UKBB sample (Supplementary Fig. 2). We defined a European subset as samples with a classifier-assigned probability exceeding 0.99 of being European. The RF classifiers were implemented using the randomForest (4.6) R library.

#### Final hard filters

We removed samples with disagreement between reported and imputed sex, removed samples with excess ultra-rare variants (URVs). We then applied a final set of variant filters, and removed variants that do not satisfy any of the criteria, and the result is illustrated in Supplementary Fig. 3:

- Non-invariant site
- Call rate ≥ 0.97
- HWE *P *≥ 10^−10^

Then we apply a second collection of sample-level filters. As an initial pass to remove low-quality and contaminated samples, we filtered out samples with a call rate  < 0.95, mean DP  < 19.5 or mean GQ  < 47.8. Using hail, we then run the *sample_qc* function to obtain and filter by four metrics. This is used to remove sample outliers based on the ratio of (1) transitions to transversions, (2) heterozygous to homozygous variants, (3) insertions to deletions, and (4) number of singletons (Supplementary Fig. 4). Samples are removed if they are more than four standard deviations away from the mean stratified by ancestry.

### Additional QC of genotyping array data

Both variants and samples from the bulk genotyping array underwent quality control by the UKBB team^52^. Variants were then lifted over from the GRCh37 to the GRCh38 genome assembly using liftovervcf (version 0.0.1), a component of the SHAPEIT5 toolkit^21^, along with the corresponding human genome reference. As an additional quality control step, we compared allele counts between sites in the genotyping array that overlapped with gnomAD (v4.0.1) Whole Genome Sequencing (WGS) data^53^. For this comparison, we first restricted the analysis to individuals of predicted European ancestry and recalculated allele frequencies and counts using PLINK v1.9. Variants showing significant discrepancies in AF between the genotyping array and gnomAD were removed (two-sided Fisher’s exact test, *P* < 1 × 10^−50^). This *P*-value threshold was selected after a comparison of multiple cutoffs and their impact on the resulting concordance between gnomAD and genotyping array AF. This final filtering step resulted in the removal of 4958 out of 684,941 variants (0.7%) where there was overlap between gnomAD and genotyping array data.

### Phasing

We integrated genotyping array data (UK BiLEVE and UKBB Axiom arrays) with exome sequencing data (IDT xGen Exome Research Panel v1.0) after quality control using Hail^48^ and BCFtools^54^ (1.12). For overlapping variants, we prioritized ES data. We excluded genotyping array variants with  >5% missingness after GRCh38 liftover using Hail^48^. We removed trio parents to avoid biasing phasing quality estimates. The phasing procedure employed a two-step SHAPEIT5^21^ (5.0.0) approach: first establishing a common variant scaffold (MAF  > 0.1%) using SHAPEIT5_PHASE_COMMON, then phasing rare variants against this scaffold with SHAPEIT5_PHASE_RARE. For computational efficiency, we processed overlapping 100,000-variant chunks with ≥ 50,000 variant overlap, trimmed 22,500 variants from each chunk boundary, and ligated the results using bcftools^54^ (1.12), retaining 5000 overlapping variants between contiguous chunks. The final dataset was restricted to samples and variants in the analysis-ready non-Finnish European (NFE) subset.

### Trio-switch error rates

We evaluated phasing accuracy by comparing statistically phased genotypes with those determined by Mendelian inheritance in 96 trios. Switch errors were identified by simultaneously traversing the statistically phased and parent-offspring transmitted haplotypes to detect phase inconsistencies between adjacent heterozygous variants. This method considered only sites where one parent was heterozygous and the other homozygous, excluding de novo variants and Mendelian inconsistencies. To assess site-specific switch errors, we modified bcftools^54^ (1.12) to output errors by genomic position, enabling analysis across variant categories (genotyping array vs. ES) and MAF bins. We evaluated error rates at different phasing confidence thresholds by filtering the VCF using Hail^48^ before recalculating switch errors. Binomial 95% confidence intervals (CIs) for switch error rates (SERs) were calculated using the Hmisc R-package^55^ (4.7).

### Variant annotation

We annotated variants using VEP v105^56^. We employed a series of tools to predict deleteriousness in silico. To discriminate between the predicted functional impact of LoF variants, we used LOFTEE v1.04^53^, which annotates PTV variants as either high confidence (HC) or low confidence (LC) LoF based on their likelihood to escape NMD or be annotation artifacts. For putatively damaging missense or protein-altering variants, we used pre-computed annotations from dbNSFP v4.3^57^, containing variant predictions from CADD v1.6^58^ and REVEL^59^. As indels were not annotated in this version of dbNSFP, we manually re-ran CADD v1.6 to annotate indels in our data. Splicing variants were annotated using SpliceAI v1.3^60^. We split all variants by transcript using bcftools +split-vep^54^ and restricted to protein-coding transcripts defined by Matched Annotation from the NCBI and EMBL-EBI (MANE) Select^61^. If no MANE Select transcript was available for the gene, we restricted to the canonical transcript as defined by GENCODE V39^62^. Ordered by predicted impact on the cognate protein, we defined the following annotations based on the hierarchy. Any given variant received one single annotation of the following:

1. **pLoF**: HC variants included stop-gained, essential splice, and frameshift variants that have been filtered by LOFTEE.
2. **Damaging missense or protein-altering variants**: Any variant that is not categorized in (1) (HC pLoF) with at least one of the following are true:
  The variant is an in-frame indel, stop-loss, start loss or missense variant with a REVEL-Score ≥ 0.773 or CADD-Phred score ≥ 28.1 ^63^.
  The SNP has a SpliceAI Δ score ≥ 0.5 for the donor loss, donor gain, acceptor loss or acceptor gain category.
  The variant has been designated as an LC PTV variant by LOFTEE.
3. **Other missense**: defined as variants that are either in-frame indel, stop-loss, start loss or missense variants that have not been categorized in (2) (damaging missense and/or protein altering).
4. **Synonymous** variants are defined as any synonymous variant with SpliceAI Δ score  < 0.5.
5. **Non-coding** variants are defined as downstream and upstream gene variants in neither of the above categories.

Finally, we removed annotations for all variants with gnomAD^53^ Max Broad ancestry AF  > 0.05. We selected this 5% threshold to maximize the number of identifiable biallelic carriers for nonadditive power, avoiding the sample size constraints of stricter cutoffs (e.g., MAF  < 0.01).

### Comparison of expected versus empirically observed CH and homozygous variation

We conducted simulations to validate the observed counts of biallelic carriers against theoretical expectations. We focused on 1174 genes containing at least one homozygous or CH carrier in our dataset. For each simulation, we generated genotypes for 176,935 unrelated individuals by sampling from a Bernoulli distribution, where the probability of success for each variant corresponded to its observed MAF. This procedure assumed variants were independent and in HWE. We performed 10 independent simulation runs and calculated the average number of expected homozygous and CH carriers per gene. By comparing these expected counts with our empirically observed values, we assessed the concordance between theoretical predictions and actual data. This comparison served as a quality control metric for our biallelic carrier identification procedure, confirming that our phasing and variant collapsing methods produced results consistent with statistical expectations.

### Encoding nonadditivity at a locus

#### Nonadditive transformation assuming Hardy Weinberg Equilibrium

We first considered a model incorporating additive and nonadditive (dominance deviation) effects, as described by Palmer et al. and elsewhere^2,4,13,64,65^:

2 $$y={X}^{A}{\beta }^{A}+{X}^{D}{\beta }^{D}+\varepsilon$$

where *y* is the standardized phenotype vector (*n* × 1) for *n* individuals, *X*^*A*^ and *X*^*D*^ are standardized additive and nonadditive genotype encoding matrices (*n* × *m*) for *m* loci, with corresponding effect size vectors *β*^*A*^ and *β*^*D*^ (*m* × 1), respectively. The noise component *ε* (*n* × 1) has mean zero and variance $1-{h}_{A}^{2}-{h}_{D}^{2}$, where ${h}_{A}^{2}$ and ${h}_{D}^{2}$ are the additive and dominance heritabilities. Assuming HWE, the nonadditive encoding maps genotypes [0, 1, 2] → [ − *p*/(1 − *p*), 1, − (1 − *p*)/*p*], where *p* is the minor allele frequency^2,4,13,64,65^. The nonadditive encoding is constructed such that it is uncorrelated with the corresponding additive encoding for that variant, which can be verified by testing *X*^*A*^ ⋅ *X*^*D*^ = 0 (up to floating point precision). To test additive and nonadditive associations at a single locus *j*, we regress *y* on the column vectors ${X}_{j}^{A}$ and ${X}_{j}^{D}$ (*n* × 1 each), interpreting the resulting scalar coefficients as estimates of ${\beta }_{j}^{A}$ and ${\beta }_{j}^{D}$.

#### Nonadditive transformation without assuming HWE

To extend the approach beyond common variants^4^, we consider a general case without assuming HWE. We parameterize the locus using genotype proportions:

3 $$\begin{array}{r}\left[\begin{array}{c}r\\ h\\ a\end{array}\right],\ \,{{\mbox{such \,that}}}\,r+h+a=1.\end{array}$$

Here, *r*, *h*, and *a* represent the proportions of homozygous reference, heterozygous, and homozygous alternate genotypes, respectively. At the gene level, these are equivalent to the proportions of wildtype, monoallelic, and biallelic carriers. We partition the genetic contributions into intercept, additive, and nonadditive components by defining the following vectors:

4 $$\begin{array}{r}{{X}^{0}}^{{\prime} }=\left[\begin{array}{c}1\\ 1\\ 1\end{array}\right],\ {{X}^{A}}^{{\prime} }=\left[\begin{array}{c}0\\ 1\\ 2\end{array}\right],\ {{X}^{D}}^{{\prime} }=\left[\begin{array}{c}0\\ 1\\ 0\end{array}\right],\end{array}$$

where ${X}^{{A}^{{\prime} }}$ reflects the number of alternate alleles, capturing additive effects, and ${X}^{{D}^{{\prime} }}$ captures effects deviating from additivity. We define the inner product according to the general genotype proportions as:

5 $$\begin{array}{r}\langle X,Y\rangle=r{X}_{0}{Y}_{0}+h{X}_{1}{Y}_{1}+a{X}_{2}{Y}_{2}.\end{array}$$

We then apply the Gram-Schmidt process to orthogonalize these vectors relative to the defined genotype proportions, enabling the partitioning of genetic effects:

6 $$\begin{array}{r}{X}^{0}=\frac{1}{\left\Vert {{X}^{0}}^{{\prime} }\right\Vert }\left[\begin{array}{c}1\\ 1\\ 1\end{array}\right]=\left[\begin{array}{c}1\\ 1\\ 1\end{array}\right],\end{array}$$

7 $$\begin{array}{rc}{X}^{A}=\frac{{{X}^{A}}^{{\prime} }-\langle {{X}^{A}}^{{\prime} },{X}^{0}\rangle {X}^{0}}{\left\Vert {{X}^{A}}^{{\prime} }-\langle {{X}^{A}}^{{\prime} },{X}^{0}\rangle {X}^{0}\right\Vert }&=\frac{1}{\sqrt{a+r-{(a-r)}^{2}}}\left[\begin{array}{c}-(h+2a)\\ 1-(h+2a)\\ 2-(h+2a)\end{array}\right]\\ \end{array}$$

8 $$\begin{array}{rcl}{X}^{D}&=\frac{{{X}^{D}}^{{\prime} }-\langle {{X}^{D}}^{{\prime} },{X}^{0}\rangle {X}^{0}-\langle {{X}^{D}}^{{\prime} },{X}^{A}\rangle {X}^{A}}{\left\Vert {{X}^{D}}^{{\prime} }-\langle {{X}^{D}}^{{\prime} },{X}^{0}\rangle {X}^{0}-\langle {{X}^{D}}^{{\prime} },{X}^{A}\rangle {X}^{A}\right\Vert }&=\frac{1}{\sqrt{har(a+r-{(a-r)}^{2})}}\left[\begin{array}{c}-ha\\ 2ar\\ -hr.\end{array}\right]\end{array}$$

A full derivation can be found in Supplementary Note 1. By substituting *p*^2^ for *r*, *q*^2^ for *a*, and 2*pq* for *h*, we can recover the HWE encoding (Supplementary Note 2), and we confirm that the two components are independent through **X**^*A*^ ⋅ **X**^*D*^ = 0, up to floating point precision.

#### Applying nonadditive transformation and scaling

The nonadditive encoding of genotypes proposed in equation (8) could produce values outside the conventional [0, 2] range, posing challenges for standard genotype representation tools, such as VCF. To normalize the nonadditive genotype encoding matrix ${X}^{D}\in {{\mathbb{R}}}^{n\times m}$ (*n* samples, *m* loci), we apply a linear transformation to map values onto [0, 2] while preserving orthogonality to the additive encoding. For each column *j* of *X*^*D*^, let

9 $${s}_{j}:={{\min }_{i}}{X}_{ij}^{D}-\epsilon,\quad {b}_{j}:={{\max }_{i}}{X}_{ij}^{D}+\epsilon,$$

for small epsilon (0 < *ϵ* ≪ 1) where *ϵ* > 0. We define the transformation:

10 $$f(x)=2\cdot \frac{x-{s}_{j}}{{b}_{j}-{s}_{j}}$$

Applied element-wise to the matrix *X*^*D*^:

11 $${{X}^{D}}_{i,j}^{{\prime\prime} }=f({X}^{D}ij)=2\cdot \frac{{X}_{ij}^{D}-{s}_{j}}{{b}_{j}-{s}_{j}}.$$

This transformation maps the minimum value to 0 + *ϵ*, the maximum to 2 − *ϵ*, and linearly interpolates others, preserving relative relationships while standardizing the range for each separate locus. Given that ${X}_{j}^{D}\cdot {X}_{j}^{A}=0$ and our transformation is linear, ${{X}^{D}}_{j}^{{\prime\prime} }\cdot {X}_{j}^{A}=0$ holds for each locus *j* without need for explicit verification.

### Determining dose-response relationships

For quantitative traits, we estimate the dose-response relationship at locus *j* by fitting two separate models: an additive model using ${X}_{j}^{A}$ (Equation (7)) and a nonadditive model using ${X}_{j}^{D}$ (Equation (8)). These models yield marginal estimates ${\hat{\beta }}_{j}^{A}$ and ${\hat{\beta }}_{j}^{D}$ for the additive and nonadditive effects, respectively. The marginal estimated genetic effect ${\hat{g}}_{j}$ associated with variation at locus *j*, which combines the additive and nonadditive contributions, is:

12 $${\hat{g}}_{j}={\hat{\beta }}_{j}^{A}{X}_{j}^{A}+{\hat{\beta }}_{j}^{D}{X}_{j}^{D}$$

The variance of this estimate can be calculated by combining the variances of the additive and nonadditive components:

13 $$\,{{\mbox{Var}}}\,\left({\hat{g}}_{j}\right)=\,{{\mbox{Var}}}\,\left({\hat{\beta }}_{j}^{A}{X}_{j}^{A}\right)+\,{{\mbox{Var}}}\,\left({\hat{\beta }}_{j}^{D}{X}_{j}^{D}\right)+2\,{{\mbox{Cov}}}\,\left({\hat{\beta }}_{j}^{A}{X}_{j}^{A},{\hat{\beta }}_{j}^{D}{X}_{j}^{D}\right)$$

For quantitative traits, $\,{{\mbox{Cov}}}\,\left({\hat{\beta }}_{j}^{A}{X}_{j}^{A},{\hat{\beta }}_{j}^{D}{X}_{j}^{D}\right)=0$ due to the orthogonality of the two components (${X}_{j}^{A}\cdot {X}_{j}^{D}=0$) and the assumption that effect size is independent of genetics, *X*. This orthogonality simplifies the variance calculation to:

14 $$\,{{\mbox{Var}}}\,\left({\hat{g}}_{j}\right)=\,{{\mbox{Var}}}\,\left({\hat{\beta }}_{j}^{A}{X}_{j}^{A}\right)+\,{{\mbox{Var}}}\,\left({\hat{\beta }}_{j}^{D}{X}_{j}^{D}\right)$$

To visualize these relationships, we directly implemented equation (12) for each locus with *d* ∈ {0, 1, 2} representing wildtype, monoallelic, and biallelic carriers. For each dosage level, we evaluated ${\hat{g}}_{j}$ by substituting the appropriate genotype values into ${X}_{j}^{A}$ and ${X}_{j}^{D}$. The standard errors were calculated following equation (14), yielding ${{\mbox{SE}}}_{{\mbox{full}},d}=\sqrt{{(d\times {{\mbox{SE}}}_{{\hat{\beta }}_{j}^{A}})}^{2}+{({X}_{j,d}^{D}\times {{\mbox{SE}}}_{{\hat{\beta }}_{j}^{D}})}^{2}}$. The combined standard errors were used to construct the 95% confidence intervals shown as gray shading in the dose-response visualizations.

### Phenotype curation

#### Medically relevant traits

We considered 55 medically-relevant quantitative traits from UKBB from our discovery analysis outlined in Supplementary Data 8 that are used to assess a broad range of pathologies. These traits included 4 Body Measurements (3 anthropometric traits such as BMI, waist-to-hip ratio, and sitting height, along with 1 Bone Mineral Density trait), cardiovascular indicators (diastolic and systolic blood pressure measurements associated with hypertension and cardiovascular disease risk), Spirometry (3 traits including FEV1 and FVC that evaluate pulmonary function and may indicate respiratory disorders such as COPD and asthma), serum biomarkers (20 biomarkers including liver enzymes, kidney function markers, and inflammatory indicators that reflect organ system health) and lipids (7 measurements including LDL, HDL, and triglycerides that assess cardiovascular and metabolic disease risk), and comprehensive blood assay measurements (19 hematological parameters including complete blood count components that can indicate various hematologic disorders, nutritional deficiencies, and inflammatory conditions). We performed basic quality control on serum biomarker traits by masking extreme outliers (> 1000 times the interquartile range) to minimize the impact of measurement errors or rare metabolic disorders on population-level associations. We applied inverse-normal transformation to all traits prior to association analysis.

### Proteomic measurements

The Olink Explore 3072 Proximity Extension Assay (PEA) platform quantifies proteins by using paired antibodies with complementary oligonucleotides that, when bound to a target protein, hybridize to create a unique barcode that undergoes Deoxyribonucleic Acid (DNA) amplification and Next Generation Sequencing (NGS) quantification^66^. Non-fasting blood samples were collected from all UKBB participants at recruitment. The Olink PEA was performed on 54,291 participants, of which we analyzed the subset of 46,595 randomly selected samples representative of the general population^27^. Proteins were measured across eight panels, which includes groups of antibodies relevant for the study of different traits (cardiometabolic, cardiometabolic II, inflammation, inflammation II, neurology, neurology II, oncology, and oncology II). The UKBB Olink dataset underwent assessment and quality control by the UK Biobank Pharma Proteomics Project (UKB-PPP) consortium team, including outlier removal, likely sample swap identification, removal of data with QC or assay warnings, and normalization. Following the manufacturer’s recommendations, we performed additional quality control: we substituted protein values below the Limit Of Detection (LOD) with $\frac{\,{\mbox{LOD}}\,}{\sqrt{2}}$ and standardized assay protein expression values to have mean zero and variance one. We applied inverse-normal transformation to all traits prior to association analysis.

### Single variant and pseudo-variant analysis

We performed both variant-level and gene-level analyses to capture the full spectrum of genetic effects: For variant-level analysis, we estimate for each locus *j*: *r*_*j*_, *h*_*j*_, and *a*_*j*_ as the proportions of homozygous reference, heterozygous, and homozygous alternate genotypes, respectively. In the gene-level analysis, we use phased genotypes and collapse multiple variants by their respective haplotypes. For each gene *g*, we set *r*_*g*_, *h*_*g*_, and *a*_*g*_ as the proportions of individuals with zero, one, and two haplotypes carrying one or more alternate alleles, respectively. For both analyses, we apply the nonadditive transformation, followed by linear re-scaling as per equation (10) to restrict ‘dosages’ to the interval [0, 2]. We modeled three encodings using SAIGE^67,68^: standard additive [0, 1, 2], recessive [0, 0, 1], and nonadditive (i.e., the dominance deviation) [ − *ha*, 2*ar*, − *hr*] (after transformation and re-scaling). To preserve floating point accuracy, the nonadditive dosages are encoded in variant call format (VCF) or plink files (.bed, .bim, and .fam).

Single-variant and pseudo-variant (gene-level) association analysis were performed using SAIGE, adjusting for the covariates sex, age, age × sex, age^2^, age^2^ × sex, PC1 − PC10. We discard variants or pseudo-variants with p.value.NA  < 0.05 AND Is.SPA = FALSE, as these loci have not converged Saddle Point Approximation (SPA) in the model. For quantitative traits, we applied a deterministic rank-based Inverse Normal Transformation (INT) based on equation (15), before analyzing them with SAIGE:

15 $${Y}_{i}^{int}={\Phi }^{-1}\left(\frac{{r}_{i}-c}{N-2c+1}\right),$$

*r*_*i*_ denotes the standard rank of the *i*th observation within the set of *N* data points, *c* is a constant offset equal to 0.5, and *Φ*^−1^(⋅) signifies the inverse function of the standard normal distribution’s cumulative distribution function (CDF), which follows *N*(0, 1), and finally ${Y}_{i}^{int}$ is the transformed phenotype value for the *i*th data point.

#### Set-based analysis of nonadditive effects

In addition to our haplotype-based pseudo-variant approach, we implemented a variant-level set-based framework for nonadditive analysis without requiring phased data. We applied our orthogonal nonadditive encoding (*X*^*D*^) within SAIGE-GENE+^26^, enabling nonadditive burden testing across sets of variants. For gene *g* containing variants *j* ∈ *S*_*g*_, the nonadditive set-based model is defined by:

16 $${y}_{i} \sim {\sum}_{j\in {S}_{g}}{w}_{j}{X}_{i,j}^{{D}^{{\prime\prime} }}$$

Where *y*_*i*_ represents the phenotype for individual *i*, ${X}_{i,j}^{{D}^{{\prime\prime} }}$ is the transformed nonadditive encoding for individual *i* at variant *j*, and *w*_*j*_ is a variant-specific weight. This framework enables various aggregation tests, including burden, SKAT^69^, and SKAT-O^70^. We implemented a beta distribution weighting scheme to prioritize rare variants, which we inputted to SAIGE-GENE + using the –weights.beta argument.

17 $${w}_{j}=\,{\mbox{Beta}}\,({{{{\rm{MAF}}}}}_{j},1,25)$$

This approach assigns higher weights to rare variants, which typically exhibit larger effect sizes^71^. We excluded ultra-rare variants (MAC < 10) from set-based analyses to ensure model stability, as SAIGE internally collapses such variants into a single unit, which is currently incompatible with our nonadditive encoding. This set-based implementation provides a complementary approach to the pseudo-variant method, enabling nonadditive testing in datasets where phased genotypes are unavailable.

#### Common variant additive and nonadditive conditional analysis

To determine whether gene-based signals were driven by nearby common variants, we performed both additive and nonadditive conditional analyses. For each gene that reached exome-wide significance in our primary analysis, we identified potential confounding common variants (MAF > 0.01) within a 1 mega base pairs (Mb) window (± 500 kb upstream and downstream) surrounding the gene. We employed this conservative threshold for conditioning (in contrast to the MAF < 0.05 threshold used for rare variant aggregation) to ensure that aggregated gene-based signals were not falsely driven by LD with intermediate frequency variants (1–5%) that would be missed if we only controlled for common variants (> 5%). For this purpose, we used the UKBB imputed genotypes, specifically excluding variants that overlapped with the exome capture regions (See UKBB Resource 3803). The conditional analysis followed a stepwise approach using SAIGE^67^ with identical covariates to our primary analysis. First, we identified the most significantly associated common variant in the region. Next, we conditioned on this variant and identified the next most significant variant. We continued this iterative process until no additional variants showed conditional significance (*P* < 5 × 10^−6^), allowing up to 25 independent associations per region to capture complex linkage patterns. These independently associated common variants were then encoded as dosages and incorporated alongside our gene-based pseudo-variants in a VCF file. This encoding allowed us to re-test each original pseudo-variant while properly accounting for these nearby common markers. By comparing the original and conditional results, we could distinguish between true gene-level effects and those potentially driven by LD with nearby common variants. We applied this conditional approach to both the additive allelic encoding and the nonadditive encoding derived from our Gram-Schmidt orthogonalization.

### Calculating statistical power advantage in partially recessive models

To compare the statistical power of additive, recessive, and joint 2-DF genetic models, we conducted analytical power calculations across varying parameters. For a sample size *n*, MAF *p*, total locus-specific heritability *h*^2^, and heterozygote effect *θ* (representing the degree of biological dominance, where *θ* = 1 indicates complete additivity and *θ* = 0 indicates complete recessivity), we calculated the expected statistical power for each model. The power of a single-term model, either additive ([0, 1, 2]) or recessive ([0, 0, 1]), was determined using the non-centrality parameter $\,{\mbox{ncp}}={(\beta /{{\mbox{SE}}}_{\beta })}^{2}$, where *β* is the relevant effect size coefficient and ${{\mbox{SE}}}_{\beta }=\sqrt{(1-{h}^{2})/n}$. For the joint 2-DF model, which includes both additive and nonadditive components, the total non-centrality parameter was calculated as the sum of individual NCPs (ncp_total_ = ncp_*a*_ + ncp_*d*_). Power was then calculated from the non-central *F*-distribution using an LRT-based framework, with degrees of freedom corresponding to each model (DF = 1 for single-term models, DF = 2 for the joint model).

Given a genetic architecture defined by *h*^2^ and *θ*, we derived the effect sizes *β*_*a*_ (additive component) and *β*_*d*_ (nonadditive component) by solving a system of linear equations that maps genotypes [0, 1, 2] to phenotype values [0, *θ*, 2], while constraining the combined variance explained to equal *h*^2^. Specifically, we constructed a matrix *A* with rows corresponding to genotypes and columns to intercept, additive, and nonadditive components, and solved *Ax* = *b* where *b* = [0, *θ*, 2]^*T*^. The resulting coefficients were then scaled to achieve the desired heritability. We evaluated power across a grid of MAF values (corresponding to homozygote counts from 5 to 500 in a cohort of 400,000 individuals, roughly corresponding to our UKBB counts) and heterozygote effects (*θ* from 0 to 1), using a significance threshold of *α* = 2.5 × 10^−7^. This framework enabled us to identify specific combinations of allele frequency and genetic architecture where the joint 2-DF model provides substantial power advantages over standard additive or recessive tests.

### Cauchy combination of *P*-values from additive and recessive models

To benchmark our 2-DF joint model against standard approaches, we implemented a CCT test^44^ to integrate evidence from separate additive and recessive models. For each gene-trait association, we combined the *P*-values from the additive ([0, 1, 2]) and recessive ([0, 0, 1]) encodings as follows:

18 $${T}_{{{{\rm{CCT}}}}}={\sum}_{i\in \{A,R\}}{\omega }_{i}\tan \left(\left(0.5-{P}_{i}\right)\pi \right),$$

where *P*_*A*_ and *P*_*R*_ represent the *P*-values from additive and recessive tests, respectively, with equal weights *ω*_*A*_ = *ω*_*R*_ = 0.5. The transformation $\tan \left((0.5-{P}_{i})\pi \right)$ follows the standard Cauchy distribution when *P*_*i*_ is uniformly distributed under the null hypothesis. The combined *P*-value was calculated as:

19 $${P}_{{{{\rm{CCT}}}}}={F}_{C}({T}_{{{{\rm{CCT}}}}})$$

Where *F*_*C*_(⋅) is the cumulative distribution function of the standard Cauchy distribution.

### Calculating joint 2-DF associations for quantitative traits

We implemented a joint 2-DF test to simultaneously evaluate additive and nonadditive genetic effects in 339,040 unrelated UKBB individuals. This approach exploits the mathematical orthogonality between our additive (*X*^*A*^) and nonadditive (*X*^*D*^) encodings, allowing direct combination of their test statistics. For each gene-trait pair, the separate *P*-values from additive (*P*_*A*_) and nonadditive (*P*_*D*_) models were converted to *χ*^2^-square statistics through the inverse *χ*^2^ transformation: ${\chi }_{A}^{2}={F}_{{\chi }_{1}^{2}}^{-1}(1-{P}_{A})$ and ${\chi }_{D}^{2}={F}_{{\chi }_{1}^{2}}^{-1}(1-{P}_{D})$. The joint test statistic was calculated as ${\chi }_{\,{\mbox{joint}}\,}^{2}={\chi }_{A}^{2}+{\chi }_{D}^{2}$, which follows a chi-square distribution with 2 degrees of freedom under the null hypothesis due to the orthogonality constraint. Joint significance was assessed as ${P}_{{{{\rm{joint}}}}}=1-{F}_{{\chi }_{2}^{2}}({\chi }_{{\mbox{joint}}\,}^{2})$.

### Simulation

#### Null simulations to assess type I error calibration

To evaluate type I error control of our nonadditive encoding framework, we simulated null phenotypes using real genotypes across all autosomes. Phenotypes **y** were simulated following the normal distribution with mean zero and covariance structure specified by the sparse genetic relatedness matrix (GRM) derived from SAIGE step 1, using sparseMVN v0.2.2^72^. We let ${{{\bf{g}}}} \sim {{{\mathcal{N}}}}({{{\bf{0}}}},{{{\bf{K}}}})$ where **K** is the GRM, and simulated:

20 $${{{\bf{y}}}}=\sqrt{{h}^{2}}{{{\bf{g}}}}+\sqrt{1-{h}^{2}}{{{\boldsymbol{\epsilon }}}},\quad {{{\boldsymbol{\epsilon }}}} \sim {{{\mathcal{N}}}}({{{\bf{0}}}},{{{\bf{I}}}})$$

where *h*^2^ represents heritability captured by common variants in the GRM. These phenotypes contain no genetic signal from the rare bi-allelic variants tested. We then varied heritability *h*^2^ ∈ {0, 0.01, 0.05, 0.2, 0.5}, simulating 4 replicates for each *h*^2^ across three different cohorts and relatedness structures (European genetic ancestry (*N *= 395,517), African genetic ancestry (*N *= 6597) and South Asian genetic ancestry (*N *= 6369), using common genotypes for each genetic ancestry in the UK Biobank data to define the GRM. This yielded 60 quantitative null traits. Each null phenotype was tested using gene-level association analysis with the nonadditive encoding (**X**^*D*^) via (i) SAIGE and REGENIE treating each gene as a pseudo-variant, and (ii) SAIGE-GENE+ burden testing. We evaluated type I error rates at thresholds through assessment of Quantile-Quantile (QQ)-plots and resulting genomic inflation factors (*λ*_GC_) stratified by AF.

### Simulation of quantitative phenotypes under diverse genetic architectures

To assess statistical power, we simulated quantitative phenotypes under a range of genetic architectures with fixed total heritability, and tested our ability to detect association signals using additive and nonadditive encodings. Architectures included additive [0, 1, 2], strictly recessive [0, 0, 2], overdominant [0, 2, 0], and partially recessive [0, *α*, 2] with varying heterozygote effects (*α* ∈ {0.05, 0.1, 0.2, 0.3}).

For each causal variant *j* with observed genotype proportions (*r*_*j*_, *h*_*j*_, *a*_*j*_) for wildtype, heterozygous, and homozygous alternate carriers, we specified a target genetic architecture ${{{{\bf{g}}}}}_{j}={[{g}_{0},{g}_{1},{g}_{2}]}^{\top }$ defining the expected phenotypic deviation for each genotype class. We decomposed this architecture into orthogonal standardized additive (${{{{\bf{X}}}}}_{j}^{A}$) and nonadditive (${{{{\bf{X}}}}}_{j}^{D}$) components (Equations (7) and (8)), solving for effect sizes ${\beta }_{j}^{A}$ and ${\beta }_{j}^{D}$ such that:

21 $${{{{\bf{g}}}}}_{j}={\mu }_{j}+{\beta }_{j}^{A}{{{{\bf{X}}}}}_{j}^{A}+{\beta }_{j}^{D}{{{{\bf{X}}}}}_{j}^{D}$$

where *μ*_*j*_ is the population mean for variant *j*. Because ${{{{\bf{X}}}}}_{j}^{A}$ and ${{{{\bf{X}}}}}_{j}^{D}$ are orthogonal with unit variance by construction, the variance contributions are ${h}_{A,j}^{2}={({\beta }_{j}^{A})}^{2}$ and ${h}_{D,j}^{2}={({\beta }_{j}^{D})}^{2}$. We then rescaled both effect sizes uniformly to achieve a target heritability ${h}_{j,\,{\mbox{target}}\,}^{2}$ while preserving the architecture-specific ratio of additive to nonadditive variance.

Phenotypes for *n* individuals with *M* causal variants were simulated as:

22 $${y}_{i}=\mu+{\sum }_{j=1}^{M}\left({\beta }_{j}^{A}{X}_{ij}^{A}+{\beta }_{j}^{D}{X}_{ij}^{D}\right)+\sqrt{1-{h}_{\,{\mbox{total}}\,}^{2}}\cdot {\epsilon }_{i},\quad {\epsilon }_{i} \sim {{{\mathcal{N}}}}(0,1)$$

where ${h}_{\,{\mbox{total}}\,}^{2}=\mathop{\sum }_{j=1}^{M}({h}_{A,j}^{2}+{h}_{D,j}^{2})$. For polygenic (*M* > 1) simulations, each variant contributed equally: ${h}_{j,\,{\mbox{target}}}^{2}={h}_{{\mbox{total}}\,}^{2}/M$.

We simulated one replicate for each combination of heritability ${h}_{\,{\mbox{total}}\,}^{2}\in \{0.0005,0.001,0.002,0.003,0.004,0.005,0.0075,0.01,0.015,0.02\}$ and genetic architecture, using *M* = 100 causal variants and *n* = 395,495 individuals of European ancestry. Causal variants were randomly selected from genes with at least five homozygous alternate carriers. Each phenotype was tested using SAIGE with additive ([0, 1, 2]), recessive ([0, 0, 1]), and nonadditive (Equation (8)) encodings. Power was calculated as the proportion of causal variants achieving significance at a given threshold (*α* ∈ {0.05, 0.01, 0.001}); Type I error was assessed using non-causal variants tested alongside the causal set.

#### Simulation of HWE divergence on orthogonal encodings

To evaluate the robustness of our general orthogonal encoding compared to the standard HWE-based encoding under deviations from HWE, we performed simulations across varying levels of inbreeding coefficients (*F*). We simulated genotypes for *N* = 10, 000 individuals and *M* = 200 independent variants for *F* ∈ {0, 0.05, 0.10, . . . , 0.35}. For each variant, the minor allele frequency *p* was sampled uniformly from ${{{\mathcal{U}}}}(0.2,0.5)$. Genotypes (*g* ∈ {0, 1, 2}) were generated using Wright’s equilibrium probabilities^24^ to incorporate the effect of inbreeding:

23 $$P(g=0)={(1-p)}^{2}+p(1-p)F$$

24 P(g=1)=2p(1−p)(1−F)

25 $$P(g=2)={p}^{2}+p(1-p)F$$

Quantitative phenotypes were simulated under a strictly additive model, representing the null hypothesis for nonadditive effects (i.e., no true deviation from additivity). We performed association testing using univariate linear regression for both the standard HWE-based nonadditive encoding (as defined in Supplementary Note 2) and the general orthogonal encoding (Equation (8)) calculated on the empirical genotype counts. We assessed the concordance between methods by calculating the Pearson correlation coefficient between the $-{\log }_{10}(P)$ derived from both encodings at each level of inbreeding.

### QC of All Of Us whole genome sequencing data

#### Summary of All Of Us whole genome sequencing quality control

For the AoU WGS (v8) data, we performed a series of filters at the sample, variant, and genotype levels using Hail 0.2^48^. We restricted our analysis to samples with well-defined ancestry predictions and excluded flagged samples according to AoU quality metrics. We excluded flagged samples identified in the AoU QC process and restricted our analysis to samples with at least one ancestry probability ≥ 0.75. For ancestry-specific analyses, we filtered to samples with the highest probability for the relevant ancestry group (EUR, AFR, American/Admixed (AMR), EAS, or SAS).

#### Variant and genotype-level filters

We applied the following filters:

1. Initial variant filters:
  - Call rate  > 0.99
  - No quality flags in the variant filter field
2. Genotype-level filters:
  - GQ  > 25
  - Filter status (FT) of “PASS" or missing
3. Final variant filters after recalculation of statistics:
  - Non-invariant sites (at least one alternate allele present)
  - Post-filtering call rate  > 0.99
  - HWE *P* > 1 × 10^−20^

For each ancestry group, we exported rare variants (MAF < 0.05) and excluded any variants in gnomAD with a frequency higher than (MAF > 0.05) across any population. We annotated variants using the same framework as described for UKBB. Based on the annotation, we amalgamated homozygous variants across genes and performed gene-based testing of biallelic carriers using SAIGE.

### Statistical analysis and visualization

Unless otherwise indicated, analyses were performed in R (4.1.1) and Python (3.6.13) and plotted using the R package ggplot2 (3.4.0).

### Reporting summary

Further information on research design is available in the Nature Portfolio Reporting Summary linked to this article.

## Supplementary information

Supplementary Information

Description of Additional Supplementary Files

Supplementary Data 1

Supplementary Data 2

Supplementary Data 3

Supplementary Data 4

Supplementary Data 5

Supplementary Data 6

Supplementary Data 7

Supplementary Data 8

Supplementary Data 9

Supplementary Data 10

Supplementary Data 11

Reporting Summary

Transparent Peer Review file

## Source data

Source data

## Data Availability

Additive and nonadditive summary statistics have been deposited at Zenodo^73^ (https://doi.org/10.5281/zenodo.18944247). UK Biobank individual-level genotype and phenotype data are available through a managed access process (https://www.ukbiobank.ac.uk/enable-your-research/apply-for-access; Application Number 11867). All of Us individual-level data are available through the All of Us Researcher Workbench (https://www.researchallofus.org) under controlled tier access. Source data are provided in this paper.

## Code availability

We provide an online browser that allows users to explore the relationship between additive and nonadditive variance across different genetic architectures here: https://frhl.github.io/sim_variance_explained/. The C++ software to perform the nonadditive allelic recoding is available on GitHub: https://github.com/frhl/call_chets. All original code used for this manuscript can be found on GitHub (https://github.com/frhl/nonadditivity) and has been archived on Zenodo^74^. All custom code developed for this study is publicly available without restriction under the MIT License.
