# Supplementary material for: Deviations from genetic additivity driven by rare variants at biobank scale
Source: Nat Commun. 2026 Aug 29;17:10302. doi: 10.1038/s41467-026-76151-w (PMC13620152; doi:10.1038/s41467-026-76151-w)
Supplement: Supplementary file 1 [file 41467_2026_76151_MOESM14_ESM.pdf]

nature portfolio

Corresponding author(s): Duncan S. Palmer

Last updated by author(s): April 13, 2026

Reporting Summary

Nature Portfolio wishes to improve the reproducibility of the work that we publish. This form provides structure for consistency and transparency in reporting. For further information on Nature Portfolio policies, see our Editorial Policies and the Editorial Policy Checklist.

Statistics

For all statistical analyses, confirm that the following items are present in the figure legend, table legend, main text, or Methods section.

n/a	Confirmed	
☐	☒	The exact sample size (*n*) for each experimental group/condition, given as a discrete number and unit of measurement
☐	☒	A statement on whether measurements were taken from distinct samples or whether the same sample was measured repeatedly
☐	☒	The statistical test(s) used AND whether they are one- or two-sided<br>*Only common tests should be described solely by name; describe more complex techniques in the Methods section.*
☐	☒	A description of all covariates tested
☐	☒	A description of any assumptions or corrections, such as tests of normality and adjustment for multiple comparisons
☐	☒	A full description of the statistical parameters including central tendency (e.g. means) or other basic estimates (e.g. regression coefficient) AND variation (e.g. standard deviation) or associated estimates of uncertainty (e.g. confidence intervals)
☐	☒	For null hypothesis testing, the test statistic (e.g. *F*, *t*, *r*) with confidence intervals, effect sizes, degrees of freedom and *P* value noted<br>*Give P values as exact values whenever suitable.*
☐	☒	For Bayesian analysis, information on the choice of priors and Markov chain Monte Carlo settings
☐	☒	For hierarchical and complex designs, identification of the appropriate level for tests and full reporting of outcomes
☐	☒	Estimates of effect sizes (e.g. Cohen's *d*, Pearson's *r*), indicating how they were calculated

Our web collection on statistics for biologists contains articles on many of the points above.

Software and code

Policy information about availability of computer code

Data collection	N/A
Data analysis	Analyses were primarily conducted in R (v4.1.1) and Python (v3.6.13). Phasing was performed using SHAPEIT5 (v5.0.0). Genetic association studies were performed using SAIGE and REGENIE. Data processing and quality control utilized Hail (v0.2), PLINK (v1.9), and BCFtools (v1.12). Custom C++ software for nonadditive allelic recoding is available on GitHub (https://github.com/frhl/call_chets), and all original analysis code is available at https://github.com/frhl/nonadditivity. Code has been archived on Zenodo (DOI: 10.5281/zenodo.19457056).

For manuscripts utilizing custom algorithms or software that are central to the research but not yet described in published literature, software must be made available to editors and reviewers. We strongly encourage code deposition in a community repository (e.g. GitHub). See the Nature Portfolio guidelines for submitting code & software for further information.

Data

Policy information about availability of data

All manuscripts must include a data availability statement. This statement should provide the following information, where applicable:

- Accession codes, unique identifiers, or web links for publicly available datasets
- A description of any restrictions on data availability
- For clinical datasets or third party data, please ensure that the statement adheres to our policy

The discovery study is based on UK Biobank data (including Olink proteomic data), which is available to approved researchers under Application Number 11867.

Replication analyses were conducted using data from the All of Us Research Program, which is available to registered researchers via the Researcher Workbench (https://www.researchallofus.org). Additive and nonadditive GWAS summary statistics generated in this study have been deposited at Zenodo and will be made publicly available upon publication. Summary statistics are deposited at Zenodo (DOI:10.5281/zenodo.18944247).

Research involving human participants, their data, or biological material

Policy information about studies with human participants or human data. See also policy information about sex, gender (identity/presentation), and sexual orientation and race, ethnicity and racism.

Reporting on sex and gender	We use the term "sex" throughout the paper to refer to biological sex. Sex was determined genetically based on the F-statistic calculated from non-pseudoautosomal regions of the X chromosome. We excluded individuals where genetically imputed sex did not match self-reported sex to identify sample swaps.
Reporting on race, ethnicity, or other socially relevant groupings	Participants were grouped based on genetic ancestry. For the discovery analysis (UK Biobank), we utilized a Random Forest classifier trained on 1000 Genomes Project super-population labels using genetic principal components. We restricted the primary analysis to individuals identified as having European genetic ancestry (probability > 0.99) to minimize confounding due to population stratification. For replication in the All of Us Research Program, we similarly utilized genetically determined ancestry predictions (probability > 0.75). In all association models, we further controlled for fine-scale population structure by including the first 10 genomic principal components (PC1–PC10) as covariates.
Population characteristics	The discovery study is based on the UK Biobank cohort, described in detail by Bycroft et al. (2018). The main analyses were performed in 399,943 individuals of European ancestry, defined as samples with a random-forest classifier-assigned probability >0.99 of being European based on principal components. We analyzed 55 quantitative traits and 2,906 plasma proteins. Replication analyses were performed in the All of Us (AoU) Research Program cohort, utilizing homozygous carriers of European and Admixed-American genetic ancestry.
Recruitment	The discovery study is based on the UK Biobank cohort, which recruited ~500,000 participants aged 40–69 years from across the United Kingdom between 2006 and 2010 (Bycroft et al. 2018). We acknowledge the "healthy volunteer" selection bias inherent to this cohort, as participants are known to be healthier and live in less socio-economically deprived areas than the general UK population (Fry et al. 2017). Replication analyses were performed in the All of Us Research Program, which recruits participants from across the United States to reflect the diversity of the US population.
Ethics oversight	This research was conducted using the UK Biobank Resource under Application Number 11867. UK Biobank has approval from the North West Multi-centre Research Ethics Committee (MREC) as a Research Tissue Bank (RTB). The All of Us Research Program is overseen by the All of Us Institutional Review Board (IRB). We utilized data available to registered researchers via the Researcher Workbench.

Note that full information on the approval of the study protocol must also be provided in the manuscript.

Field-specific reporting

Please select the one below that is the best fit for your research. If you are not sure, read the appropriate sections before making your selection.

☒ Life sciences ☐ Behavioural & social sciences ☐ Ecological, evolutionary & environmental sciences

For a reference copy of the document with all sections, see nature.com/documents/nr-reporting-summary-flat.pdf

Life sciences study design

All studies must disclose on these points even when the disclosure is negative.

Sample size	No statistical methods were used to predetermine sample size. The study utilized the maximum available sample size from the UK Biobank (N=399,943 European ancestry individuals) to maximize statistical power for detecting rare variant associations.
Data exclusions	We excluded samples based on standard quality control criteria established prior to analysis: (1) disagreement between self-reported and genetically imputed sex; (2) excessive heterozygosity or missingness; (3) relatedness (keeping only unrelated individuals, for a single seperate analysis); and (4) individuals not confidently classified as European ancestry (probability > 0.99) to prevent confounding by population stratification. For the proteomic analysis, we used the subset of 46,595 samples passing UKB-PPP quality control.
Replication	We sought to replicate significant nonadditive gene-trait associations in the All of Us Research Program (AoU). We successfully replicated associations for BTNL9 (HDL cholesterol), FUT10 (COPD), and TM6SF2 (Lipids). Some associations (e.g., FLG and eosinophil counts) could not be replicated in AoU due to the lower sample size and lack of sufficient biallelic carriers in that cohort compared to UK Biobank, and thus ORs could not be obtained.Details of all replication are provided in the manuscript.
Randomization	N/A. This was an observational study based on existing biobank data; participants were not randomized to experimental groups.
Blinding	N/A. Blinding was not applicable as this was a computational analysis of pre-existing data where genotypes and phenotypes were determined prior to the study.

Reporting for specific materials, systems and methods

We require information from authors about some types of materials, experimental systems and methods used in many studies. Here, indicate whether each material, system or method listed is relevant to your study. If you are not sure if a list item applies to your research, read the appropriate section before selecting a response.

Materials & experimental systems

n/a	Involved in the study
☒	☐ Antibodies
☒	☐ Eukaryotic cell lines
☒	☐ Palaeontology and archaeology
☒	☐ Animals and other organisms
☒	☐ Clinical data
☒	☐ Dual use research of concern
☒	☐ Plants

Methods

n/a	Involved in the study
☒	☐ ChIP-seq
☒	☐ Flow cytometry
☒	☐ MRI-based neuroimaging

Plants

Seed stocks	N/A
Novel plant genotypes	N/A
Authentication	N/A
