# Supplementary material for: Deviations from genetic additivity driven by rare variants at biobank scale
Source: Nat Commun. 2026 Aug 29;17:10302. doi: 10.1038/s41467-026-76151-w (PMC13620152; doi:10.1038/s41467-026-76151-w)
Supplement: Supplementary file 2 [file 41467_2026_76151_MOESM15_ESM.pdf]

nature portfolio

Peer Review File

Deviations from genetic additivity driven by rare variants at biobank scale

Corresponding Author: Dr Frederik Lassen

This file contains all reviewer reports in order by version, followed by all author rebuttals in order by version.

Version 0:

Reviewer comments:

Reviewer #1

(Remarks to the Author)
In this work, Lassen et al. proposes a new framework to identify non-additive effects for rare variants. They propose a new framework based on phased data (to model compound heterozygotes) and recoding of genotypes (to use fast existing association tools). They apply this framework to UK Biobank (UKBB) protein level data and 55 quantitative traits, allowing to identify 10 gene:protein pairs and 11 gene:traits with non-additive effects, respectively. Some of these findings were replicated using the All of Us cohort.

Overall, this paper addresses a fundamental and under-explored question in genetics. The paper well written and the analyses of the UK Biobank data are extremely rigorous. I have some concerns regarding the interpretation of results and the comparison with other approaches.

MAJOR

- Some of the paper's conclusion are overstated. For example, claiming that non-additive effects are "prevalent and play a central role in disease and biological processes" (line 475) seems to be an exaggeration; while their existence is supported, the evidence presented doesn't seem to justify the claim of widespread prevalence.

- The new framework is not rigorously compared to alternative approaches. For instance, the authors claim that some findings from recessive models are false positives (lines 367-369), but this statement is not supported by simulations or analysis. A small simulation subsection at the beginning of the Results section would strengthen the paper by demonstrating the framework's benefits and showing when it can detect non-additive effects missed by the additive and recessive approaches. Similarly, are there any scenarios where additive tests can detect non-additive effects missed by the non-additive approach (due to power)? In addition, I would appreciate a comparison with results from ref. 25.

- Similarly, the recessive approach identified 22 gene-traits pairs, including 5 suggested as false positive. The number of remaining pairs (17) is higher than what has been observed by the proposed framework (11). How should we interpret these results?

- It's unclear why the search for non-additive effects was applied to complex traits but not to proteomic data. If no direct effects were observed in the proteomic analysis, that's an important result that should be reported. The authors should clarify this decision and potentially update the legend of Figure 2 to reflect this finding.

MINOR

- The example on lines 300-311 is confusing. The authors should clarify if the nearby intronic variant is part of the recessive combination and if adding it to the burden score would affect the additive and non-additive signals differently. Providing the rsID for the variant would also be helpful.

- Page 16: "with points along the diagonal line indicating additive relationships" and "Relationships that are additive follow x = y" are redundant.

(Remarks on code availability)

Reviewer #2

(Remarks to the Author)
The manuscript by F. Lassen et al. presents a new model for testing non-additive effects of rare variants in quantitative traits. The study is well-designed, and the manuscript is clearly written. I do, however, have several questions and comments that I believe would strengthen the work:

1. The manuscript is primarily focused on gene-based analyses. However, the framework could, in principle, be applied to variant-level analyses as well (L91). I understand that such analyses would likely be underpowered, but it would be useful to include some discussion of this possibility.

2. The authors report that 10 out of 136 additive genes (7.4%) show evidence of non-additive effects. In a similar study of the human metabolome focused on common variation, 2 out of ~20 genes (~10%) exhibited non-additive effects (PMID: PMC4512538). It would be valuable if the authors could provide some speculation on how widespread non-additive inheritance might be, both among rare and common variants associated with quantitative traits.

3. Related to the above point: since the non-additive transformation framework should in principle be applicable to common variants as well, could the authors comment on whether their approach can be extended to common variant analyses?

4. It would be helpful if the discussion included some practical recommendations for applying the framework to large-scale data—for example, suggested thresholds for MAF and p-values. Moreover, as the authors note, phasing is unlikely to be feasible in very large biobank-scale datasets. Realistically, the framework will often be applied to unphased data. Could the authors comment on the expected loss of power and accuracy in this case?

(Remarks on code availability)
I haven't thoroughly checked the code.

1. https://github.com/frhl/nonadditivity/tree/main/scripts – this looks fine to me.
2. https://github.com/frhl/call_checks – more practical examples of how to run it on unphased data are needed.
3. https://frhl.github.io/sim_variance_explained/ — looks fine to me.

Reviewer #3

(Remarks to the Author)
Summary:

The manuscript “Deviations from genetic additivity driven by rare variants at biobank scale” presents an innovative non-additive encoding that is by construction statistically orthogonal to the standard allele count encoding and avoids assumptions of Hardy-Weinberg equilibrium (HWE). Based on this framework, the authors report significant trait associations in large-scale human genetic data from the UK Biobank with validation experiments done with individuals from the All of Us (AoU) project. The manuscript is clearly written, and the motivation for the new encoding is compelling. Overall, we feel like, with a few additional analyses, this work will make a valuable contribution to the field. Below, we outline several suggestions that we think could further strengthen the work.

Major Comments:

Null simulations that demonstrate the calibration of the method: With this being a methods style paper, perhaps the most important piece that is missing from the current draft is an in-depth simulation study which demonstrates both the behavior of the proposed framework under the null hypothesis and its power under the alternative. These aspects are key since they will give readers an idea of when to expect the method to produce false positives and under what conditions the method is best suited to be applied. To the author’s credit, the current version of the manuscript states that their new encoding is well calibrated based on simulations (lines 396-398); however, unless we are mistaken, we could not find supporting figures or tables anywhere. On line 145, the authors point to Figures S6 and S7 as evidence, but these QQ-plots were generated from real data. Since the true genetic architecture of real traits is unknown, the authors should demonstrate calibration taking real genotypes, simulating null phenotypes (i.e., traits with only additive contributions at realistic heritability), and then applying their non-additive encoding. It would be helpful to do this at (i) different sample sizes, (ii) trait heritability levels, and (iii) types of population structure so that readers get a true sense of the method’s ability to control for type I error rates. For example, they could track type I error rates at different thresholds 0.05, 0.01, and 0.001. Lastly, it would be good to know that calibration is maintained whether their coding is used in an LMM like SAIGE or in a set-based analysis like SAIGE-GENE.

Simulations that demonstrate the power of the method: Similar to our previous point, the authors should also conduct a simulation study that demonstrates when one should expect their proposed encoding to have enough power to detect real non-additive signals. Here, we could imagine the authors again simulating synthetic traits from real genotypes and estimating their ability to identify true positives while varying factors like (i) different sample sizes, (ii) trait heritability levels,

(iii) types of population structure, (iv) generative assumptions (e.g., is the underlying trait recessive), and (v) proportion of phenotypic variation that is generated by additive effects versus non-additive effects. As pointed out in our last comment, it would be great to know the power of the proposed approach in both LMMs and set-based tests.

Other simulation scenarios to consider: While not completely necessary, other simulations that could be nice to explore involve investigating the statistical behavior of this encoding when a portion of causal variants with additive and/or non-additive effects are unobserved. Another interesting setting would be investigating the behavior of the encoding when additive and non-additive effects in the generative model are heavily correlated. Would either of these mess with the power, false discovery rate, and calibration of the approach?

Sensitivity of encoding to choice of modeling architectures: In both the Introduction (lines 88-90) and Discussion (lines 425-427), the authors make a claim that their Gram-Schmidt orthogonalization-derived recoding integrates seamlessly with existing LMM tools such as SAIGE, REGENIE, and FastGWA-GLMM. However, they only demonstrate this in SAIGE. This raises an interesting question: are these results model dependent? Since the authors mention these approaches by name, they could show that their simulated and real data results hold with at least one of these other methods.

Partitioning of additive and non-additive genetic effects: In different areas throughout the manuscript (e.g., lines 19-22, 97-99, 329-333), the authors claim that this newly proposed encoding enables the accurate additive and nonadditive heritability components at biobank scale. We might have missed this, but I don’t think there are any results that back up this claim. In addition to checking the model calibration and power, does this newly proposed encoding of non-additive effects lead to more accurate variance component estimates? This can be checked in simulations and it would be nice if the authors could report these estimates in their real data analyses.

Sensitivity to the scaling done to the phenotype: We found the choice to apply an inverse-normal transformation to all traits to be an important one. What was the reason for this choice? How does this transformation affect the findings presented in the paper? It may also be informative to include results based on normalized (but not rank-transformed) traits for comparison.

Minor Comments:

It would be helpful to include a clear description of how population structure was accounted for in all these analyses. For example, was this handled directly by SAIGE, by fitting the null model with a particular encoding, or by including some number of PCs? Making this explicit will help readers and reviewers evaluate the robustness of the results.

The conclusion that one in three cognate gene-protein pairs exhibit nonlinear dose-response effects is very interesting. However, since this is based on only 24 data points, we would encourage the authors to provide an estimate of statistical uncertainty to help readers gauge confidence in this result. In connection with our earlier comments, this makes providing simulation studies even more important. It would be great to understand at what sample size one would expect to see power.

The threshold for distinguishing common vs. rare variants is not used consistently (sometimes MAF ≥ 0.01, sometimes ≥ 0.05). Standardizing this throughout will improve clarity.

Figure S6: The QQ-plot axis labels and captions seem to contain contradictions. Aligning these descriptions will avoid confusion.

(Remarks on code availability)
It appears that the code appears in different places. The C++ software to perform the non-additive recoding is available here: https://github.com/frhl/call_chets. And then the code used in the manuscript can be found on here https://github.com/frhl/nonadditivity.

It would appear that all the scripts necessary are provided in both of these resources; however, we would encourage the authors to create more detailed READMEs in both repos to better facilitate the implementation of their approach and the reproducibility of their work.

Reviewer #4

(Remarks to the Author)
I co-reviewed this manuscript with one of the reviewers who provided the listed reports. This is part of the Nature Communications initiative to facilitate training in peer review and to provide appropriate recognition for Early Career Researchers who co-review manuscripts.

(Remarks on code availability)

Version 1:

Reviewer comments:

Reviewer #1

(Remarks to the Author)
The authors have addressed all of my comments and the updated manuscript is clear and reads well. In particular, they have made a substantial effort to add a new simulation section. I think that the paper is ready for publication.

(Remarks on code availability)

Reviewer #2

(Remarks to the Author)
All my comments were addressed. I don't have any more comments.

(Remarks on code availability)
Thank you for updating the practical examples and code.

Reviewer #3

(Remarks to the Author)
The authors have satisfied all our comments and concerns. We appreciate how thorough they were in their responses and the time put into this revision.

(Remarks on code availability)
We still think that the repo to reproduce the study (https://github.com/frhl/nonadditivity) could use a more detailed README to better orient readers on what scripts can be used to reproduce which results from the paper. Still, the authors have done significant work to address our comments from the last round.

Open Access This Peer Review File is licensed under a Creative Commons Attribution 4.0 International License, which permits use, sharing, adaptation, distribution and reproduction in any medium or format, as long as you give appropriate credit to the original author(s) and the source, provide a link to the Creative Commons license, and indicate if changes were made.
In cases where reviewers are anonymous, credit should be given to 'Anonymous Referee' and the source.
The images or other third party material in this Peer Review File are included in the article's Creative Commons license, unless indicated otherwise in a credit line to the material. If material is not included in the article's Creative Commons license and your intended use is not permitted by statutory regulation or exceeds the permitted use, you will need to obtain permission directly from the copyright holder.
To view a copy of this license, visit https://creativecommons.org/licenses/by/4.0/

(NCOMMS-25-51202-T)

Deviations from genetic additivity driven by rare variants at biobank scale

Revision

Please see below for our detailed responses to the reviewer's comments. The reviewers' original comments are in black and our replies are in blue. Major changes that we have made in the manuscript, including additional analysis and improvements, are in **bold blue text**.

Reviewer #1 (Remarks to the Author)

In this work, Lassen et al. proposes a new framework to identify effects for rare variants. They propose a new framework based on phased data (to model compound heterozygotes) and recoding of genotypes (to use fast existing association tools). They apply this framework to UK Biobank (UKBB) protein level data and 55 quantitative traits, allowing to identify 10 gene:protein pairs and 11 gene:traits with effects, respectively. Some of these findings were replicated using the All of Us cohort.

Overall, this paper addresses a fundamental and under-explored question in genetics. The paper is well written and the analyses of the UK Biobank data are extremely rigorous. I have some concerns regarding the interpretation of results and the comparison with other approaches.

MAJOR

Some of the paper's conclusions are overstated. For example, claiming that effects are "prevalent and play a central role in disease and biological processes" (line 475) seems to be an exaggeration; while their existence is supported, the evidence presented doesn't seem to justify the claim of widespread prevalence.

We have toned down the language. This portion of the main text now reads (Lines 476–479):

"Through application to proteomic and complex trait data, with replication in an independent cohort, we have demonstrated that rare variant nonadditive genetic effects are detectable and contribute meaningfully to disease risk and biological processes."

We thank the reviewer for the comment and for the opportunity to improve upon the precision of our manuscript.

The new framework is not rigorously compared to alternative approaches. For instance, the authors claim that some findings from recessive models are false positives (lines 367-369), but this statement is not supported by simulations or analysis. A small simulation subsection at the beginning of the Results section would strengthen the paper by demonstrating the framework's benefits and showing when it can detect effects missed by the additive and recessive approaches. Similarly, are there any scenarios where additive tests can detect effects missed by the approach (due to power)? In addition, I would appreciate a comparison with results from ref. 25.

Clarification on Model Inference

We thank the reviewer for the comment. For lines 367–369, we would like to clarify what we are doing. Traditionally, when attempting to identify the genetic architecture at a gene or locus, "additive" models ([0, 1, 2] encoding) and "recessive" models ([0, 1, 2] → [0, 0, 2] recoding) are used, and their P-values or effect sizes are compared in order to make statements about the underlying mode of inheritance (for example, see Heyne et al., 2024).

In our analysis, we point out that this framework often falls short. This is because of the correlation between the additive and recessive encoding, which increases as a function of

MAF. Hence, a variant that is more common, with a partially recessive genetic architecture, is more likely to have $P_{add} < P_{rec}$, and thus be misclassified as additive in spite of the underlying architecture exhibiting partial recessivity.

Simulation studies

We agree with the reviewer that simulation studies would improve the quality of the manuscript. To rigorously validate our framework, we ran a suite of simulations using genetic data taken from the UK Biobank:

- **Null Calibration** (detailed in Methods, Lines 950-965): We simulated random phenotypes across a range of heritabilities to demonstrate that our tests control the Type I error (Supplementary Figure S6). We confirmed this holds true across different software implementations, observing high concordance between SAIGE and REGENIE ($r > 0.90$; Supplementary Figure S7), and is robust to phenotype transformations (Supplementary Figure S11).
- **Power and Architecture Detection** (detailed in Methods, Lines 966-990): We simulated traits under additive ([0, 1, 2]), recessive ([0, 0, 2]), and partially recessive ([0, a, 2] where $a < 1$) architectures.
  - **Additive (Negative Control):** Our method correctly yields no signal (flat uniform P-values), confirming successful orthogonalization (Supplementary Figure S8-S9).
  - **Recessive (Positive Control):** Our method maximizes power compared to the standard additive test (Supplementary Figure S8-S9).
  - **Partially Recessive:** We demonstrate our ability to disentangle effects, identifying both a significant additive component and a significant nonadditive deviation, whereas standard models would force a binary choice (Supplementary Figure S8 and S10).
- **Robustness to HWE Violations** (detailed in Methods, Lines 991-1000): We simulated increasing levels of inbreeding (F) to compare our method against the standard HWE-dependent encoding used in prior studies (e.g., Palmer et al., 2023). As shown in Supplementary Figure S14, standard encodings exhibit a large degree of inflation as HWE is violated, whereas our orthogonal encoding maintains calibration.

We thank the reviewer for the opportunity to improve our paper by suggesting these simulation studies.

Comparison with Dhindsa et al., 2023.

We agree that comparing our results with Dhindsa et al. (2023) would provide valuable context. To facilitate this, we contacted the corresponding author, who provided us with summary statistics from their variant-level exome-wide Olink association analysis using recessive ([0, 0, 1]) and additive ([0, 1, 2]) encodings of genotypes against the trait.

We compared our pLoF results (Supplementary Table 6) with their corresponding protein-truncating variants for gene-traits where we observe a nominally significant nonadditive effect (nonadditive $P < 0.05$). We observed very high concordance between the ORs of both the additive (Pearson's $r = 0.995$) and recessive ($r = 0.996$) models between the two works,

suggesting that our results are reproducible. However, unlike Dhindsa et al., our framework allows more nuanced insights into the underlying dose-response relationship between incremental allelic substitution and protein abundance, which we outline in a few examples below.

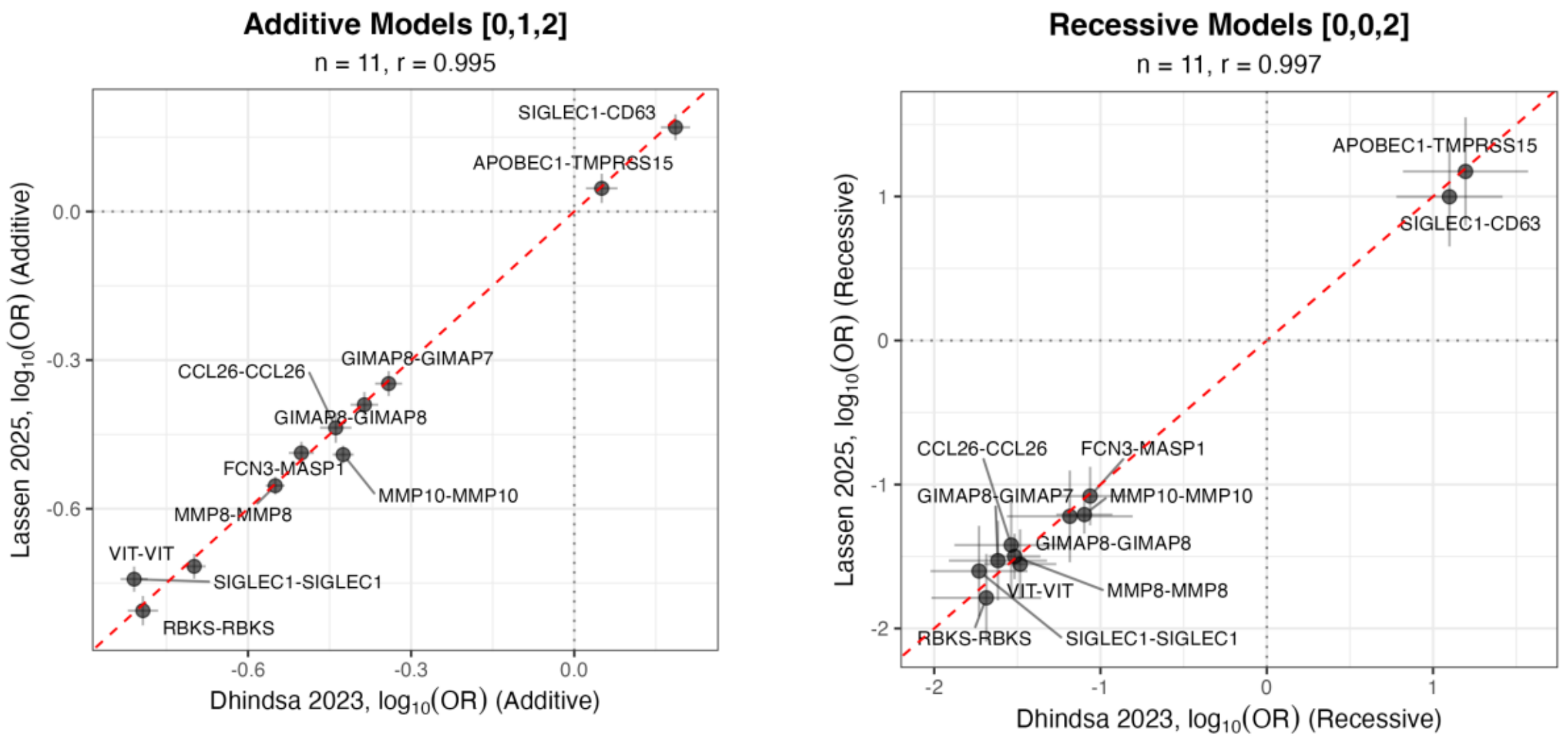

For example, they identify a significant additive relationship between incremental allelic substitution of *GIMAP8* and expression of LGALS3 (additive $P = 2.12 \times 10^{-31}$), and report a non-significant recessive relationship (recessive $P > 0.01$). We observe a similar relationship in our data (additive $P = 1.77 \times 10^{-35}$, recessive $P = 0.04$). However, we observe a nonadditive $P = 2.69 \times 10^{-5}$, suggesting that there is significant deviation from additivity, which we show in Figure 2D.

The problem that arises had we followed the Dhindsa et al. framework is that we might believe that there is a positive relationship between *GIMAP8* and LGALS3, when in reality this is only true at loss of a single allele. In the case of complete, biallelic loss, the expression is significantly reduced compared to wildtypes. Such insights can have profound implications for drug development. **We have added the above figure to the final manuscript to show (Figure S15)** that we have high concordance with the work that we are comparing against, while demonstrating the value of the nonadditive encoding.

Similarly, the recessive approach identified 22 gene-traits pairs, including 5 suggested as false positive. The number of remaining pairs (17) is higher than what has been observed by the proposed framework (11). How should we interpret these results?

We thank the reviewer for their comment and agree that this should be clarified. The value of our framework comes from inferring the underlying genetic architecture, and specifically discerning additive from recessive genetic architectures. Although the 22 gene-traits were discovered by fitting a recessive ([0, 0, 1]) model; our framework shows that there is insufficient evidence in the data to show that their effects deviate from additivity.

Of the 22 gene-traits, we then continue to compare the P-values between recessive and additive models, which has previously been proposed as a method to decide which traits are truly recessive (Heyne et al., 2022, *Nature*). Under this comparison, we observe that 7 gene-traits fit this criteria ($P_{rec} < P_{add}$) and would be reported as recessive. This method has high specificity but can miss partially recessive gene-trait associations. When we run our method, we find 12 gene-trait associations that are either recessive or partially recessive.

The remaining 17 exhibit some nominal nonadditive contribution ($P < 0.05$). However, after correcting for multiple testing, only 12 remained significant (nonadditive $P < 10^{-7}$).

It's unclear why the search for effects was applied to complex traits but not to proteomic data. If no direct effects were observed in the proteomic analysis, that's an important result that should be reported. The authors should clarify this decision and potentially update the legend of Figure 2 to reflect this finding.

We agree that this should be clarified. We wanted to showcase two different ways of searching for nonadditive effects that were not data-driven inferences that violate independence assumptions. In the proteomic data, we first fit an additive model, restrict to significant gene-traits, and then within these traits, we search for nonadditive effects. This has two consequences: (1) we reduce the multiple testing burden (which we were concerned about given the number of tests) in a manner that does not violate statistical independence (as the effects we search for are uncorrelated with the additive effect by construction), and (2) we are able to prioritize gene-traits that have at least some additive component, with a prior that the collection of genetic architectures that have some additive component that we would be powered to detect are more biologically plausible.

Had we directly fit a nonadditive model, we would have discovered 6 significant gene-traits (nonadditive $P < 1.17 \times 10^{-7}$), one of which is likely completely recessive (APOBEC1-TMPRSS15; additive $P = 0.002$, nonadditive $P = 5.53 \times 10^{-9}$, recessive $P = 9.71 \times 10^{-10}$). For the medically relevant traits, we chose to instead fit a nonadditive model directly first, in a hypothesis-free manner. Either choice is statistically justified, but depends on the question that is of interest.

We have clarified this in Lines 163-174, explaining our two-stage approach and noting that a direct search would have identified 6 associations including the likely recessive *APOBEC1*-TMPRSS15 relationship.

MINOR

The example on lines 300-311 is confusing. The authors should clarify if the nearby intronic variant is part of the recessive combination and if adding it to the burden score would affect the additive and signals differently. Providing the rsID for the variant would also be helpful.

We thank the reviewer for identifying this confusing section. **We have revised lines** 300-311 (now lines 296-300 in the revised manuscript) **to clarify that the nearby intronic variant (rs138692142) is not part of our rare variant burden**, but rather a common variant we conditioned on to assess independence from nearby common genetic variation. **We have also added the rsID and clarified how the additive and nonadditive signals are affected differently by this conditioning**.

Page 16: “with points along the diagonal line indicating additive relationships” and “Relationships that are additive follow x = y” are redundant.

We thank the reviewer for the comment. We agree that this was redundant and **we have removed the duplicate text from the revised manuscript.**

Reviewer #2 (Remarks to the Author)

The manuscript by F. Lassen et al. presents a new model for testing effects of rare variants in quantitative traits. The study is well-designed, and the manuscript is clearly written. I do, however, have several questions and comments that I believe would strengthen the work:

1. The manuscript is primarily focused on gene-based analyses. However, the framework could, in principle, be applied to variant-level analyses as well (L91). I understand that such analyses would likely be underpowered, but it would be useful to include some discussion of this possibility.

We agree with the reviewer that our framework is fully applicable to variant-level analyses, similar to what has previously been done by Palmer et al., 2023 for common variants. We have expanded both the manuscript and the software package to explicitly support this. We have added a standalone function, recode.cpp, to our codebase (available at https://github.com/frhl/call_chets and Docker Hub), along with an examples/variants directory containing documentation and toy examples to guide users in testing for nonadditive effects at the single-variant level.

To demonstrate this functionality, we performed a targeted variant-level scan across the chromosomes harboring the 10 significant gene-trait pairs identified in our primary analysis (Figure R2C1A). After applying strict Bonferroni correction for 14,475 tests ($P < 3.45 \times 10^{-6}$), we identified 8 significant variant-trait associations. These hits directly correspond to the driver variants of our gene-level signals, including specific variants in *RBKS* (chr2:27858572:C:G), *VIT* (chr2:36773813:C:G), and *CCL26* (chr7:75771945:C:T).

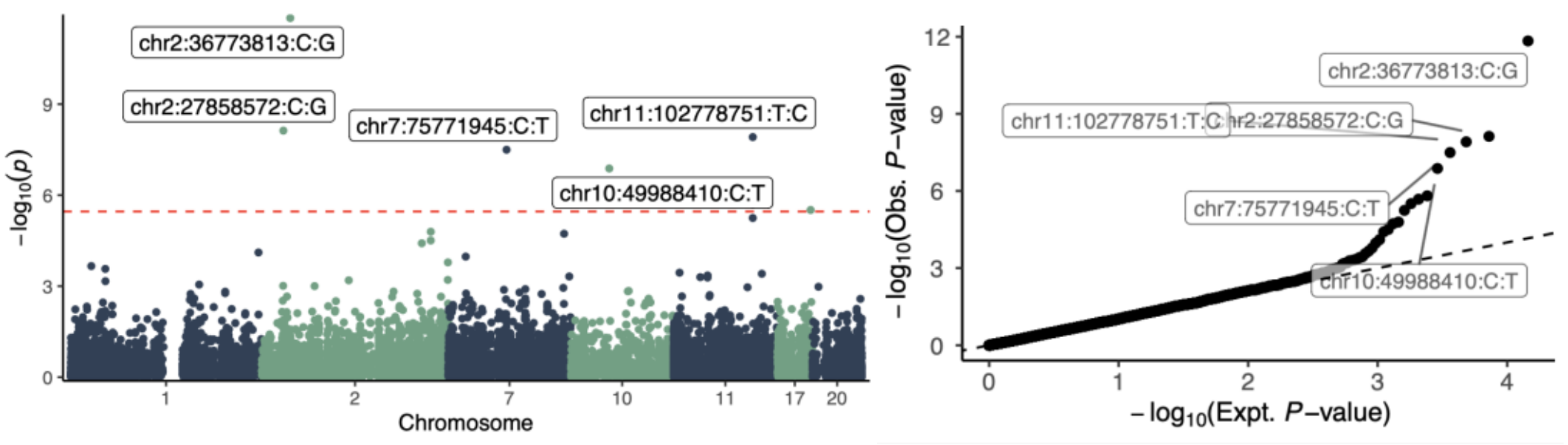

Figure R2C1A: Variant-level association scan for selected chromosomes. (Left) Manhattan plot displaying results for variants on chromosomes containing the 10 significant gene-trait pairs from the primary analysis. The red dashed line indicates the Bonferroni-corrected significance threshold ($P < 3.45 \times 10^{-6}$; correcting for 14,475 tests). Significant hits, such as those in *RBKS*, *VIT*, and *CCL26*, align with the gene-level findings. (Right) QQ plot of the nonadditive association statistics, demonstrating well-calibrated P-values with a clear deviation from the null distribution only in the tail.

The accompanying Manhattan and QQ plots (Figure R2C1A) confirm that the test statistics are well-calibrated, showing no inflation under the null but a clear deviation for likely genuine associations. Thus, while we maintain that gene-level collapsing is necessary to maximize power for rare variants generally, this validation confirms that our orthogonal encoding is robust and effective for variant-level discovery when allele frequencies or effect sizes are sufficient.

We have added these points to the manuscript: **"(..), we also demonstrate compatibility with single-variant analyses and standard set-based association methods such as in the software SAIGE-GENE+, which do not require phased genotypes"** (Lines 147-150). We have also updated the github repository and **added worked examples on how to perform set-based analysis.** We thank the reviewer for the comment and for the opportunity to showcase how the framework can be applied to a scenario that is often more practical.

2. The authors report that 10 out of 136 additive genes (7.4%) show evidence of effects. In a similar study of the human metabolome focused on common variation, 2 out of ~20 genes (~10%) exhibited effects (PMID: PMC4512538). It would be valuable if the authors could provide some speculation on how widespread inheritance might be, both among rare and common variants associated with quantitative traits.

We thank the reviewer for providing this comparison. It is encouraging that our observed rate of detectable nonadditivity (7.4%) aligns with previous estimates from the human metabolome (~10%).

We speculate that the true prevalence of biological nonadditivity (e.g., recessive inheritance) is likely higher than these figures suggest, particularly for rare variants. However, detection is limited by the rapid decay of dominance variance for a fixed effect size as the minor allele frequency decreases, as described by Zhu et al. (2015).

In the case of rare variation with a completely recessive mode of inheritance for a paritcular trait: because the phenotypic effect is restricted to rare homozygotes, the additive model (anchored by the abundant, non-effect heterozygotes) explains almost none of the variance. While this means the relative genetic signal is almost entirely nonadditive, the absolute phenotypic variance generated is vanishingly small (proportional to p^2).

Thus, although rare recessive variant effects comprise a largely nonadditive contribution, they typically do not generate enough absolute variance to be detected by exome-wide scans. Our estimate of 7.4% therefore likely represents a lower bound, capturing only the subset of genes where allele frequencies are sufficiently high (or effect sizes sufficiently large) to generate a statistically detectable deviation from additivity.

Finally, nonadditive inheritance is likely widespread, and at extremely large sample sizes, we are likely to detect statistically significant yet minor departures from linearity across the genome. However, such minor deviations are less likely to be biologically meaningful, requiring us to distinguish between statistical significance and biological relevance. **We have expanded the Discussion (lines 452-463) to explicitly cover these points:**

1. Why our 7.4% estimate is likely a lower bound due to the rapid decay of dominance variance at low MAF.
2. The future challenge of distinguishing statistically significant minor deviations (as biobanks grow) from biologically relevant nonadditivity.

3. Related to the above point: since the transformation framework should in principle be applicable to common variants as well, could the authors comment on whether their approach can be extended to common variant analyses?

We thank the reviewer for this important question. Yes, our framework is directly applicable to common variants. As we demonstrate in Supplementary Note 3, when genotype proportions follow HWE, our general transformation (Equation 21-23) mathematically reduces to the established HWE-dependent encoding [-p/(1-p), 1, -(1-p)/p] that has been successfully used in prior common variant studies (Zhu et al. 2015, Hivert et al. 2021, Palmer et al. 2023).

However, for common variants, our approach offers minimal practical advantage over existing methods. Common variants typically satisfy HWE assumptions in genetic datasets that have passed stringent quality control, as standard QC procedures typically exclude common variants deviating significantly from HWE ($P < 10^{-6}$ to 10^{-10}; Bycroft et al. 2018, Karczewski et al. 2020, etc). As a result, the HWE-based encoding remains appropriate and computationally efficient for the common variant regime.

As an example, we ran a simulation framework for common variants that applies our encoding and the HWE-dependent encoding [-p/(1-p), 1, -(1-p)/p], and compared the results. The resultant P-values are highly correlated as shown below, confirming that our method converges mathematically to the standard approach when HWE assumptions are met.

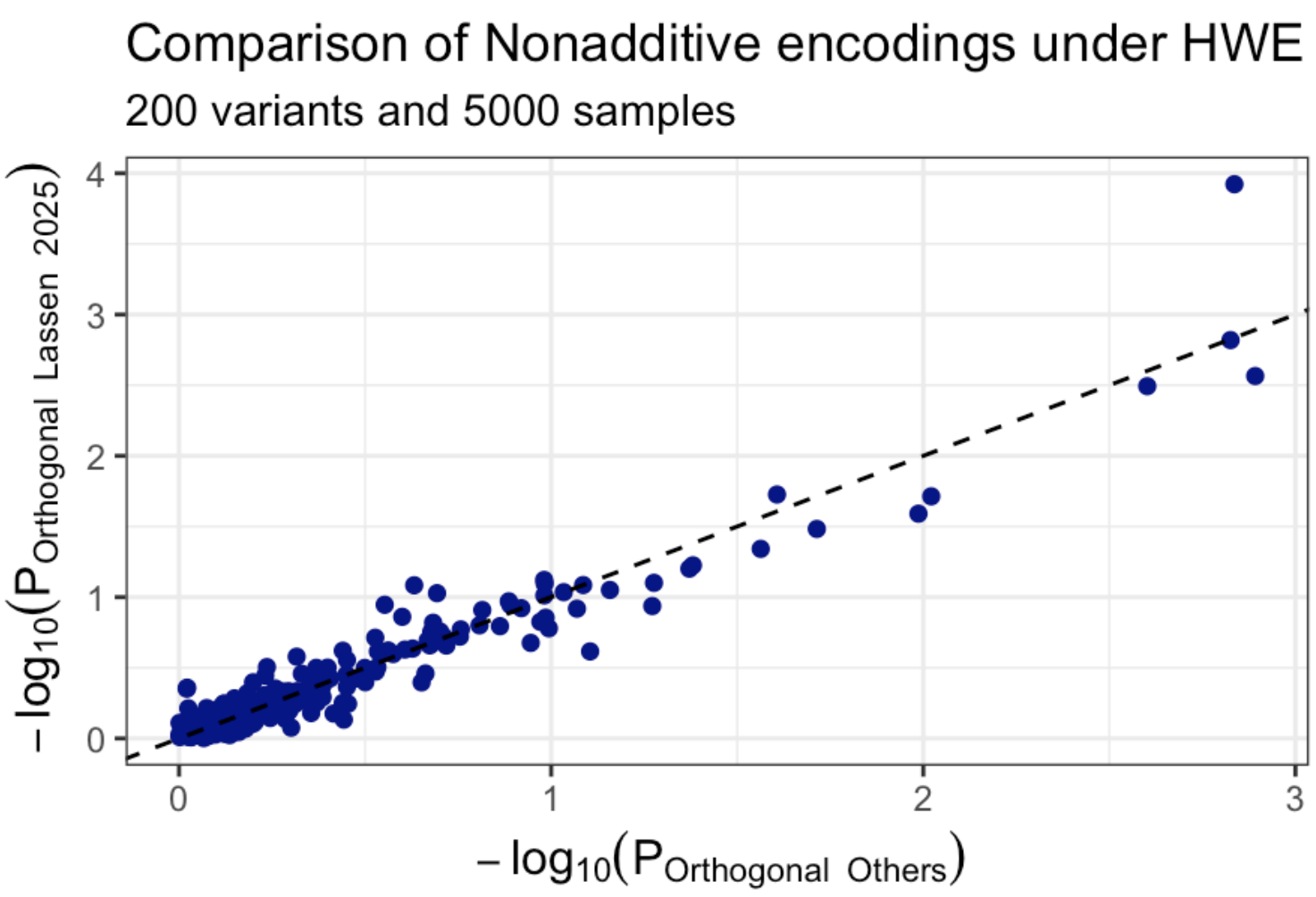

Figure R2C3A: Comparison of P-values derived from our proposed orthogonal encoding versus the standard HWE-dependent encoding under Hardy-Weinberg equilibrium. Genotypes were simulated for 5,000 individuals across 200 independent variants with minor allele frequencies sampled from U(0.2, 0.5). Phenotypes were simulated under a purely additive architecture (i.e., no true nonadditive effect), representing the null hypothesis for both nonadditive tests. Each point represents a single variant, with the x-axis showing $-\log_{10}$ (P) from the standard HWE-based encoding and the y-axis showing $-\log_{10}$ (P) from our proposed encoding.

However, the standard encoding fails when the population deviates from genotype expectations assumed by HWE. This is a scenario frequently observed with rare variants due to sampling variance, or in populations that are admixed, have experienced severe bottlenecks or inbreeding.

To show this, we simulated a purely additive trait under increasing levels of inbreeding (F) with code provided in the GitHub repository. As the deviation from HWE increases, the HWE-dependent method begins to capture the additive signal, causing the correlation between the two methods to collapse and the HWE-dependent method to produce inflated statistics. The

practical consequence of this is a catastrophic inflation of false positives. As the plot shows, this can occur even for moderate departures from HWE.

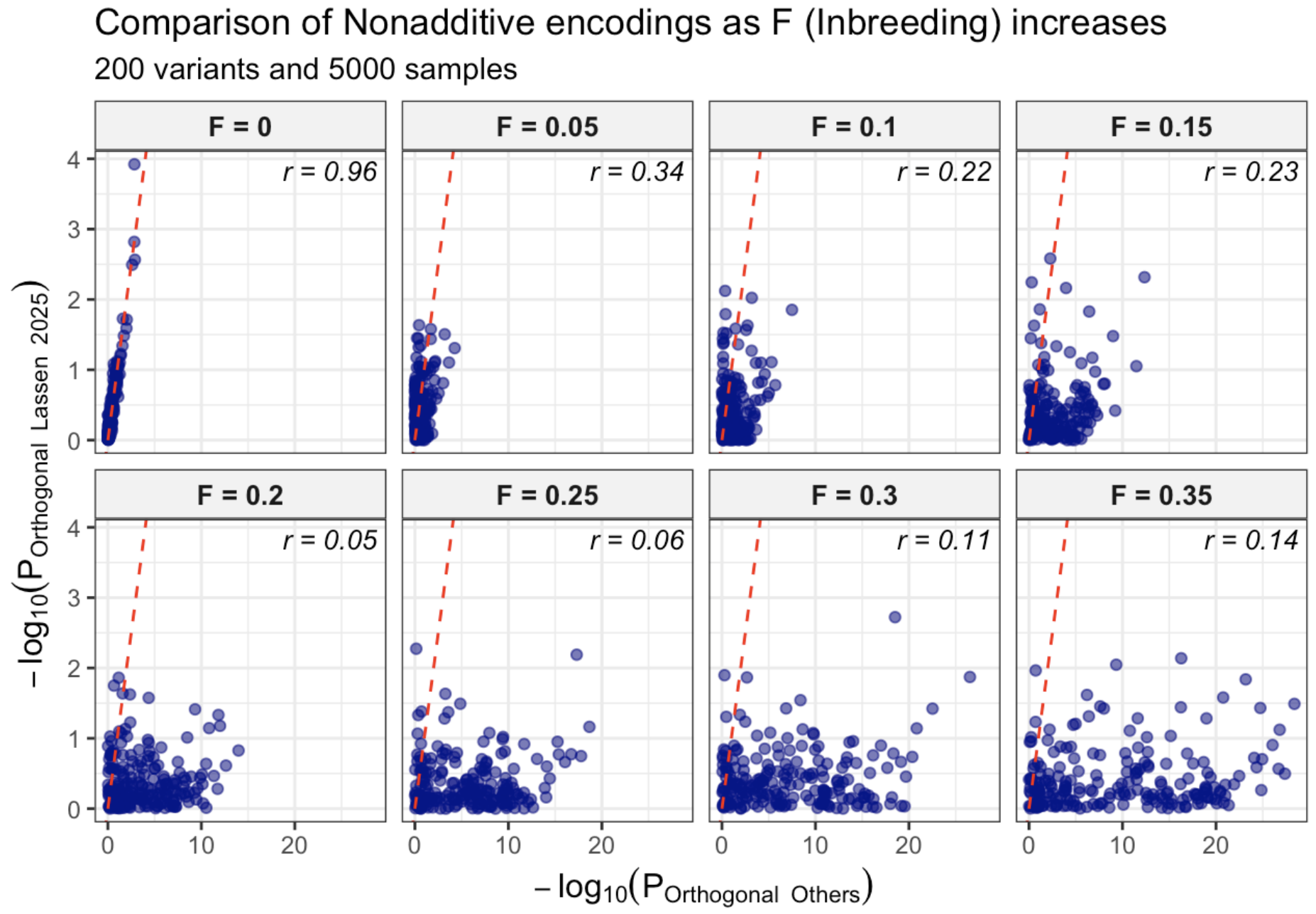

Figure R2C3B: As the inbreeding coefficient (F) increases, departure from HWE occurs, and the standard encoding [-p/(1-p), 1, -(1-p)/p] loses orthogonality, leading to divergent P-values compared to the method we present. The top right number is the Pearson correlation.

We believe this is important to show. **We have therefore added the above figure (Figure S14), clarified these advantages in the introduction (Lines 132-136), and added an explicit statement in Supplementary Note 3 that substitution of HWE proportions recovers the classical encoding.** We again thank the reviewer for the comment and the opportunity to improve upon our manuscript.

4. It would be helpful if the discussion included some practical recommendations for applying the framework to large-scale data—for example, suggested thresholds for MAF and p-values. Moreover, as the authors note, phasing is unlikely to be feasible in very large biobank-scale datasets. Realistically, the framework will often be applied to unphased data. Could the authors comment on the expected loss of power and accuracy in this case?

We thank the reviewer for this important practical consideration. We have made several improvements to address these concerns regarding practical implementation and the feasibility of applying our framework to unphased data.

Regarding practical recommendations and code availability, **we have substantially updated our GitHub repository** (https://github.com/frhl/nonadditivity) to include worked examples with simulated data that users can run to understand the framework, as well as a comprehensive best practices guide for nonadditive effect analysis. This guide covers:

- Recommended MAF thresholds (MAF < 0.05 for rare variants)

- Minimum carrier count requirements (at least 5 biallelic carriers for gene-level and variant-based tests)
- Quality control recommendations specific to nonadditive analyses
- Guidance on when to use variant-level versus gene-level approaches

We have also **included computational workflow scripts** demonstrating integration with SAIGE and SAIGE-GENE+ alongside **worked examples with simulated data.**

Regarding power and accuracy without phasing, this is indeed a critical practical question. As we demonstrate in Supplementary Note 1 and Figure S11, our framework can be applied to unphased data using standard set-based burden tests such as SAIGE-GENE+, though with some power loss when compound heterozygous variants contribute substantially to the signal.

We have added a collapsing feature to the framework so that variants can be collapsed across homozygous carriers. While this approach has reduced power compared to including compound heterozygous carriers, it remains well-calibrated under the null, which we show in simulations, and can provide important information in the absence of phased data.

I haven't thoroughly checked the code.

1. https://github.com/frhl/nonadditivity/tree/main/scripts – this looks fine to me.
2. https://github.com/frhl/call_checks – more practical examples of how to run it on unphased data are needed.
3. https://frhl.github.io/sim_variance_explained/ — looks fine to me.

We thank the reviewer for the comment and for the opportunity to improve our code. **We have now reworked the call_chets repository and added many more practical examples** for how to run the code for:

1. Without phased data at the variant-level or gene-level collapsing
2. With phased data at the variant-level or gene-level collapsing

We have also created (64) extensive unit tests to increase the robustness of our software.

Reviewer #3 (Remarks to the Author):

Summary

The manuscript “Deviations from genetic additivity driven by rare variants at biobank scale” presents an innovative encoding that is by construction statistically orthogonal to the standard allele count encoding and avoids assumptions of Hardy-Weinberg equilibrium (HWE). Based on this framework, the authors report significant trait associations in large-scale human genetic data from the UK Biobank with validation experiments done with individuals from the All of Us (AoU) project. The manuscript is clearly written, and the motivation for the new encoding is compelling. Overall, we feel like, with a few additional analyses, this work will make a valuable contribution to the field. Below, we outline several suggestions that we think could further strengthen the work.

Major Comments

Null simulations that demonstrate the calibration of the method: With this being a methods style paper, perhaps the most important piece that is missing from the current draft is an in-depth simulation study which demonstrates both the behavior of the proposed framework under the null hypothesis and its power under the alternative. These aspects are key since they will give readers an idea of when to expect the method to produce false positives and under what conditions the method is best suited to be applied. To the author’s credit, the current version of the manuscript states that their new encoding is well calibrated based on simulations (lines 396-398); however, unless we are mistaken, we could not find supporting figures or tables anywhere. On line 145, the authors point to Figures S6 and S7 as evidence, but these QQ-plots were generated from real data. Since the true genetic architecture of real traits is unknown, the authors should demonstrate calibration taking real genotypes, simulating null phenotypes (i.e., traits with only additive contributions at realistic heritability), and then applying their encoding. It would be helpful to do this at (i) different sample sizes, (ii) trait heritability levels, and (iii) types of population structure so that readers get a true sense of the method’s ability to control for type I error rates. For example, they could track type I error rates at different thresholds 0.05, 0.01, and 0.001. Lastly, it would be good to know that calibration is maintained whether their coding is used in an LMM like SAIGE or in a set-based analysis like SAIGE-GENE.

We thank the reviewer for this important query, and we welcome the opportunity to clarify how the nonadditive recoding influences the false discovery rate.

We simulated null phenotypes using real genotypes across all autosomes, with a covariance structure defined by the sparse genetic relatedness matrix from common variants. **We have added the ‘Null simulations to assess type I error calibration’ methods section to the paper to explain this in more detail and provided the code in the GitHub repository.**

We simulated using the same 400k Europeans used in the paper and evaluated the calibration via QQ-plots and the genomic inflation factor (λ_{GC}). Across all simulations, we observed a median inflation factor of [1.011] (encoding), suggesting that the models are indeed well-calibrated under the null across the allele frequency spectrum and a variety of heritability levels ($h^2 \in \{0.01, 0.2, 0.5\}$).

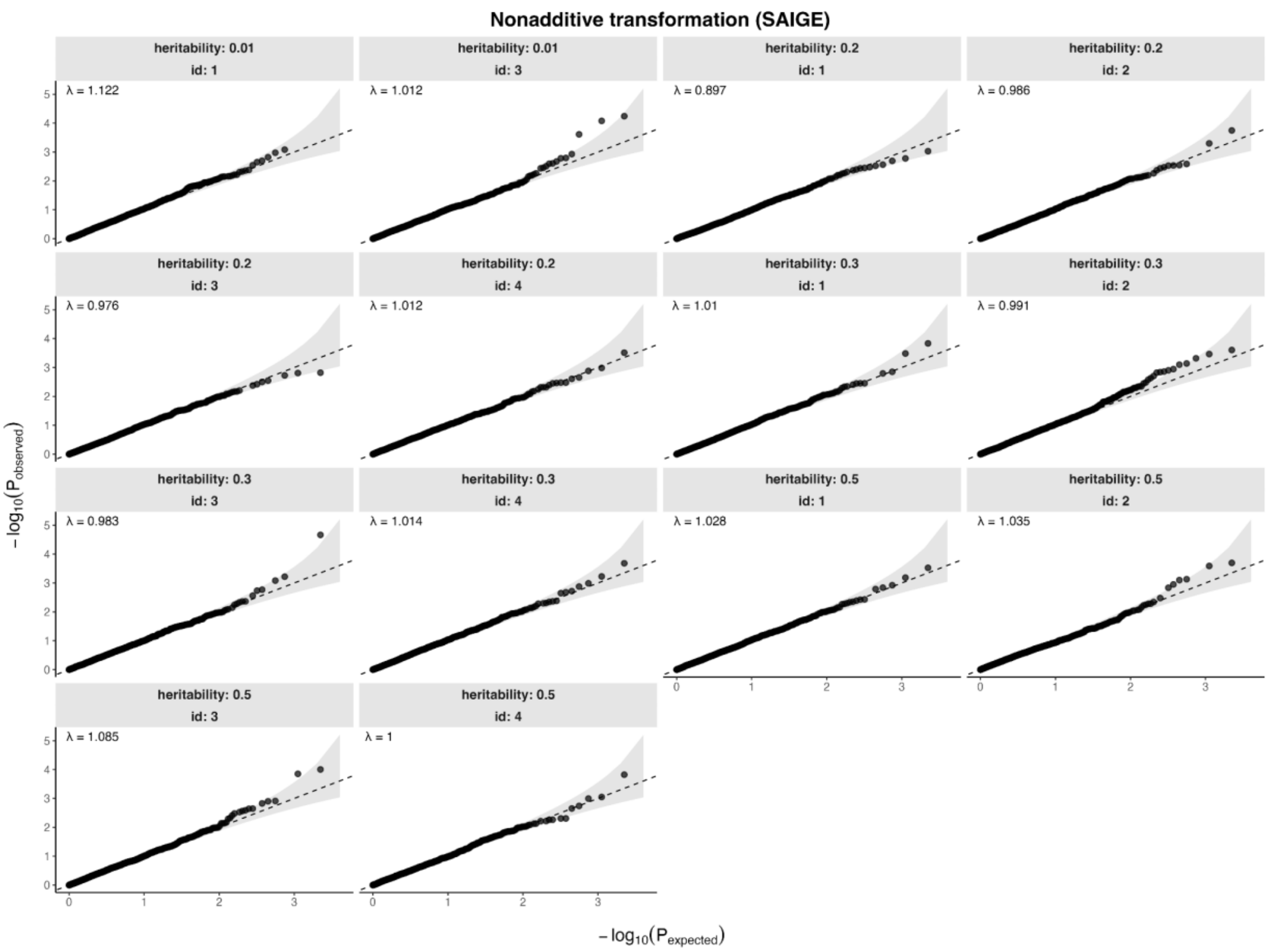

Figure R3C1A: QQ plots demonstrating calibration of the nonadditive encoding in SAIGE across various heritability levels (0.01 to 0.5) under the null hypothesis. The shaded area represents the 95% confidence interval.

To provide a quantitative assessment of calibration beyond visual inspection, we calculated the empirical Type I error rates at standard significance thresholds (α). Across our null simulations, the observed false positive rates closely matched theoretical expectations: for $\alpha = 0.05$, the median empirical rate was 0.051; for $\alpha = 0.01$, the rate was 0.010; and for $\alpha = 0.001$, the median rate was 0.0012, confirming that the method accurately controls error rates at both nominal and more stringent thresholds.

To address the reviewer's request regarding population structure and sample size, in addition to the full European (N=395,495) analysis, we performed null simulations to subsets of the UK Biobank with African (N=6,600) and South Asian (N=6,369) genetic ancestry. In all scenarios, including those with lower sample sizes and distinct linkage disequilibrium patterns, test statistics were well-calibrated with median inflation of 0.992 and 1.003 for South Asian and African genetic ancestry, respectively. Below, we show the QQ plot for South Asians with traits simulated under the null.

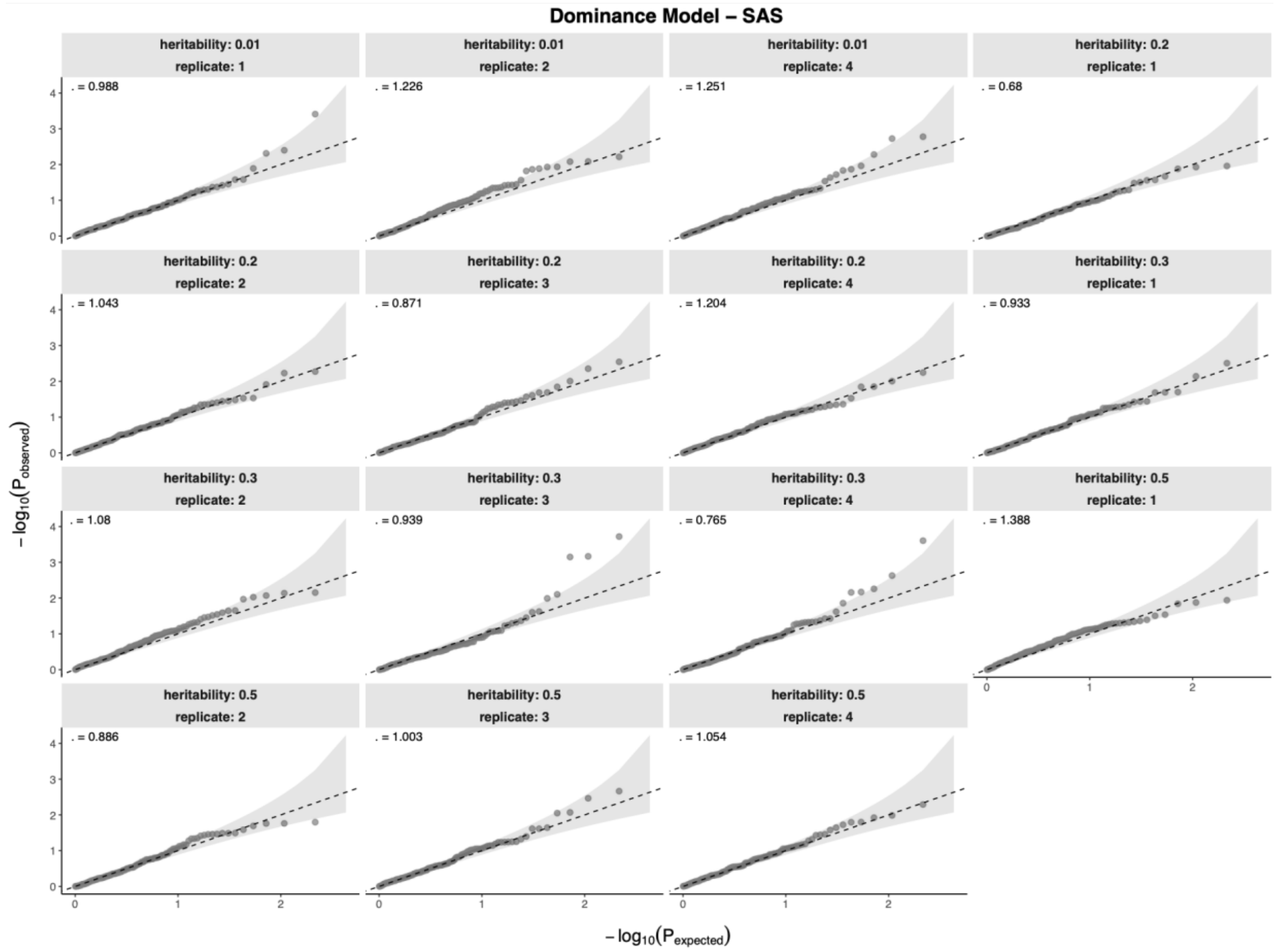

Figure R3C1B: QQ-plots demonstrating calibration of the encoding in SAIGE across various heritability levels under the null hypothesis when simulating traits for individuals of south east asian genetic ancestry (N=6,636).

To check that this holds true for other rare association test software that allows for the incorporation of related individuals, we repeated null simulations in Europeans (with the same simulated traits) using REGENIE. Resultant test statistics were well-calibrated under the null, with a median genomic inflation factor of 1.0025.

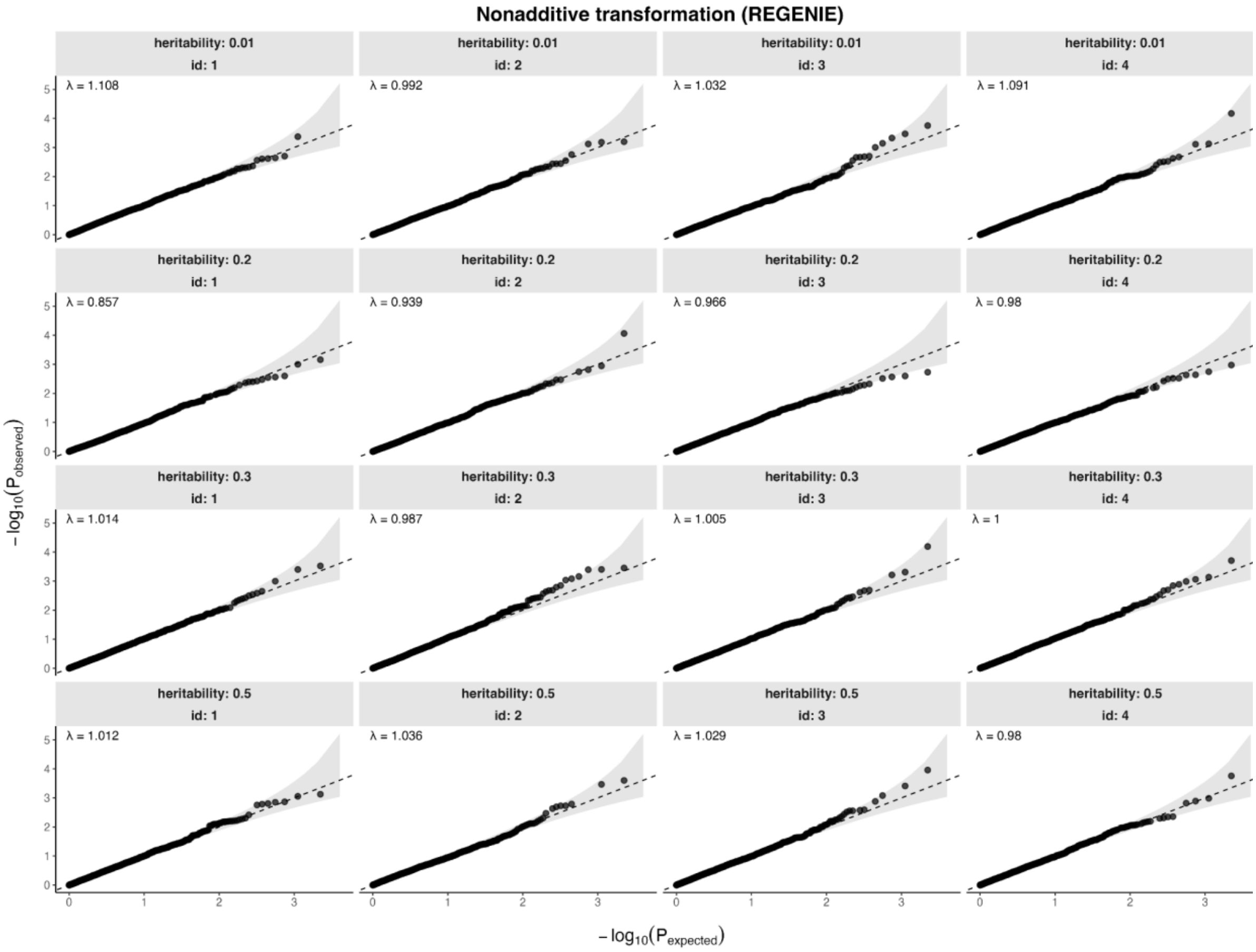

Figure R3C1C: QQ-plots demonstrating calibration of the encoding in REGENIE across various heritability levels under the null hypothesis. This analysis was run for individuals of European genetic ancestry.

When, comparing resultant $-\log_{10}$ -transformed P-values between REGENIE and SAIGE, we observe very high correlation for each simulated trait, as expected.

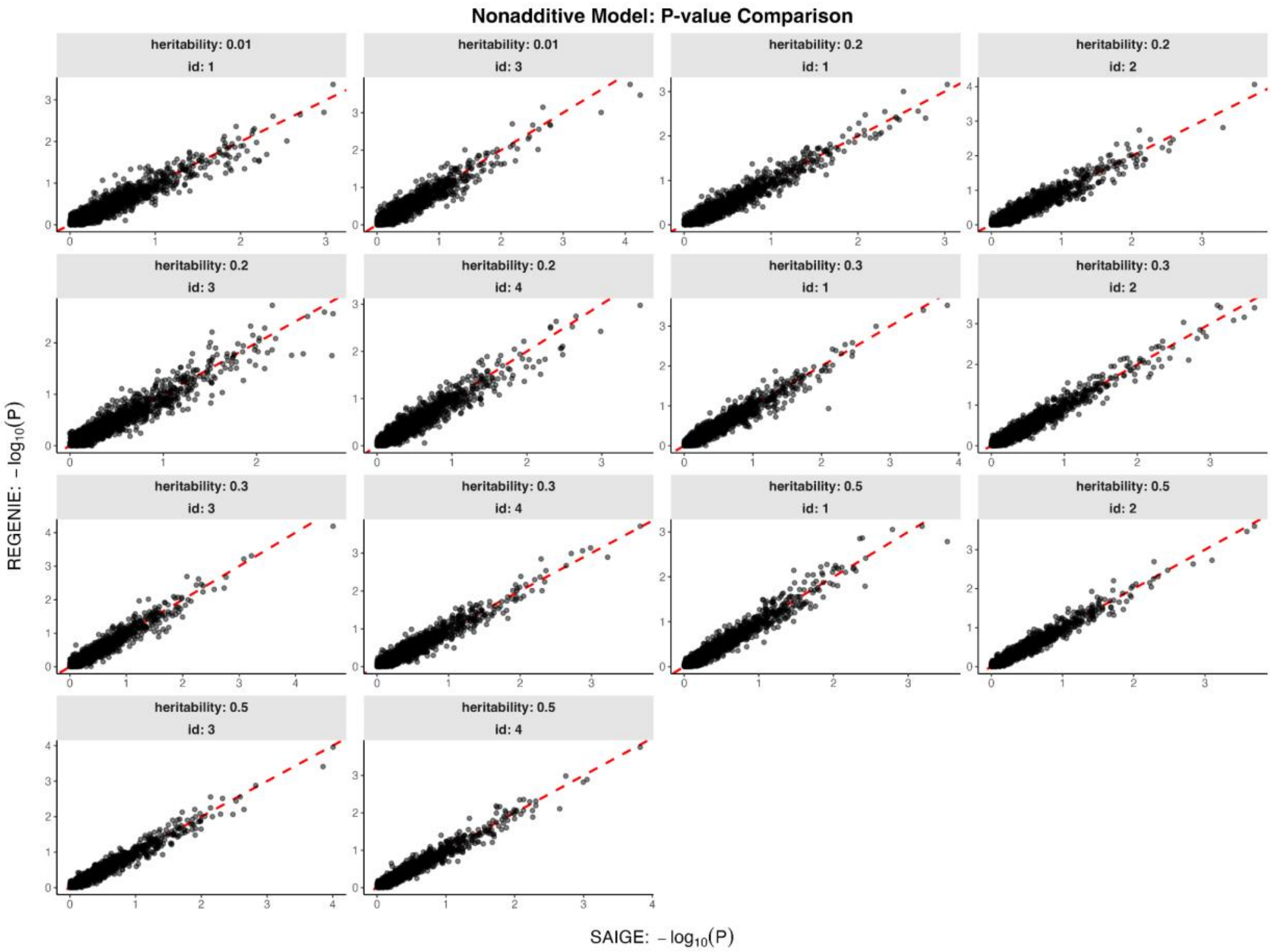

Figure R3C1D: Comparison of P-values (-$\log_{10}$ scale) generated by SAIGE and REGENIE using the nonadditive encoding after simulating phenotypes under the null hypothesis. The red dashed line represents the identity line (y=x).

Finally, we showcase that the set-based analysis of SAIGE-GENE+ also produces well-calibrated Type I errors when utilizing the nonadditive encoding. Using pLoF and missense variants from chromosomes 20–22, we demonstrate that the model is well-calibrated under the null for both single variants and set-based tests. In conclusion, we observed a median inflation factor of 0.98 and 1.0015 for the variant-based and set-based testing, respectively.

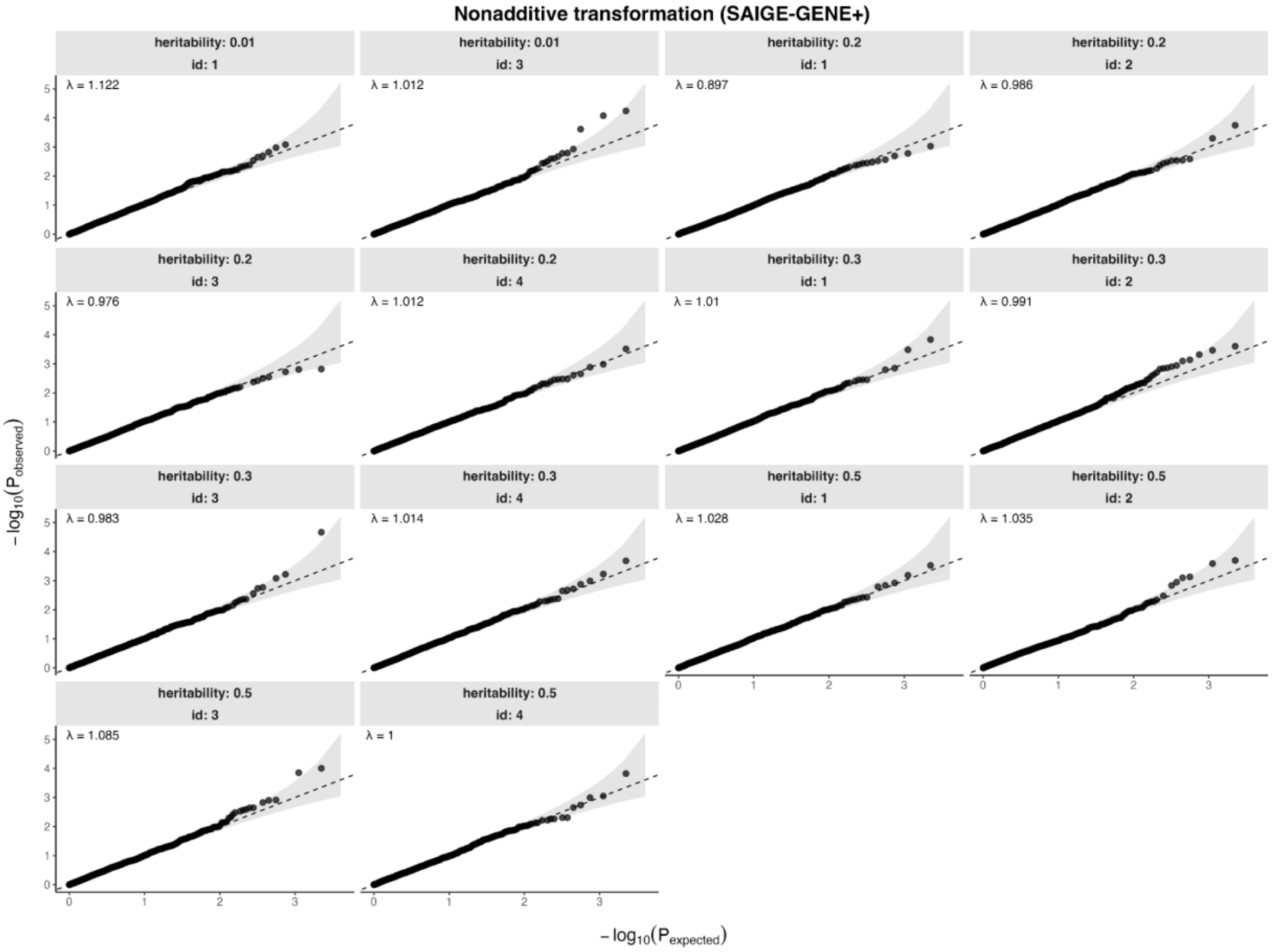

Figure R3C1E: QQ-plots demonstrating calibration of the encoding in SAIGE-GENE+ (set-based burden testing) after simulating phenotypes under the null hypothesis.

We believe that these results demonstrate that the orthogonal encoding preserves the statistical validity of the underlying linear-mixed effects model. By maintaining calibration across diverse sample sizes, ancestries, and algorithms, the framework ensures robust Type I error control without requiring modifications to standard quality control pipelines. As a result, **we have added the Figures S6-7 to the revised manuscript and comment on the calibration on lines 129-132.**

Regarding the reviewer's request to demonstrate the power under the alternative, we have addressed this extensively in the response to the subsequent comment (see 'Simulations that demonstrate the power of the method' below), where we detail the simulation of synthetic traits under additive, recessive, and overdominant architectures.

Simulations that demonstrate the power of the method: Similar to our previous point, the authors should also conduct a simulation study that demonstrates when one should expect their proposed encoding to have enough power to detect real signals. Here, we could imagine the authors again simulating synthetic traits from real genotypes and estimating their ability to identify true positives while varying factors like (i) different sample sizes, (ii) trait heritability levels, (iii) types of population structure, (iv) generative assumptions (e.g., is the underlying trait recessive), and (v) proportion of phenotypic variation that is generated by additive effects

versus effects. As pointed out in our last comment, it would be great to know the power of the proposed approach in both LMMs and set-based tests.

To address this question, we set up a comprehensive simulation framework that **we have added to the github and outlined the math in the methods section named 'Simulation of Quantitative Phenotypes Under Diverse Genetic Architectures of Causal Effects'**. Briefly, we simulated quantitative traits from real genotypes under four distinct genetic architectures (additive, partially recessive, strictly recessive, and overdominant) that naturally produce different ratios of additive to variance. We systematically varied total heritability (0.05%, 0.1%, 0.2%, 0.3%, 0.5%, 0.75%, 1%, 2%), simulating 100 causal pseudo-variants with at least five biallelic carriers, and a sample size of 395,495 individuals with European ancestry.

This framework carefully preserves the architecture-specific ratio of additive to effects while scaling to achieve target heritabilities, allowing us to compare various scenarios where effects contribute varying proportions of genetic variance (ranging from 0% in purely additive architectures to 100% in strictly recessive architectures). For each simulated phenotype, we tested association using two encodings (nonadditive and additive) with SAIGE's LMM framework, enabling direct comparisons.

Variants with effects simulated under the null remained well-calibrated throughout all simulation conditions (consistent with our null simulations above). Moreover, we were able to capture the signal of all variants with non-null genetic effects across all our generative assumptions. The resulting power plots across additive, recessive and our nonadditive encoding is shown in Figure R2C2A, and we provide QQ-plots below. We have a few key takeaways from these simulations.

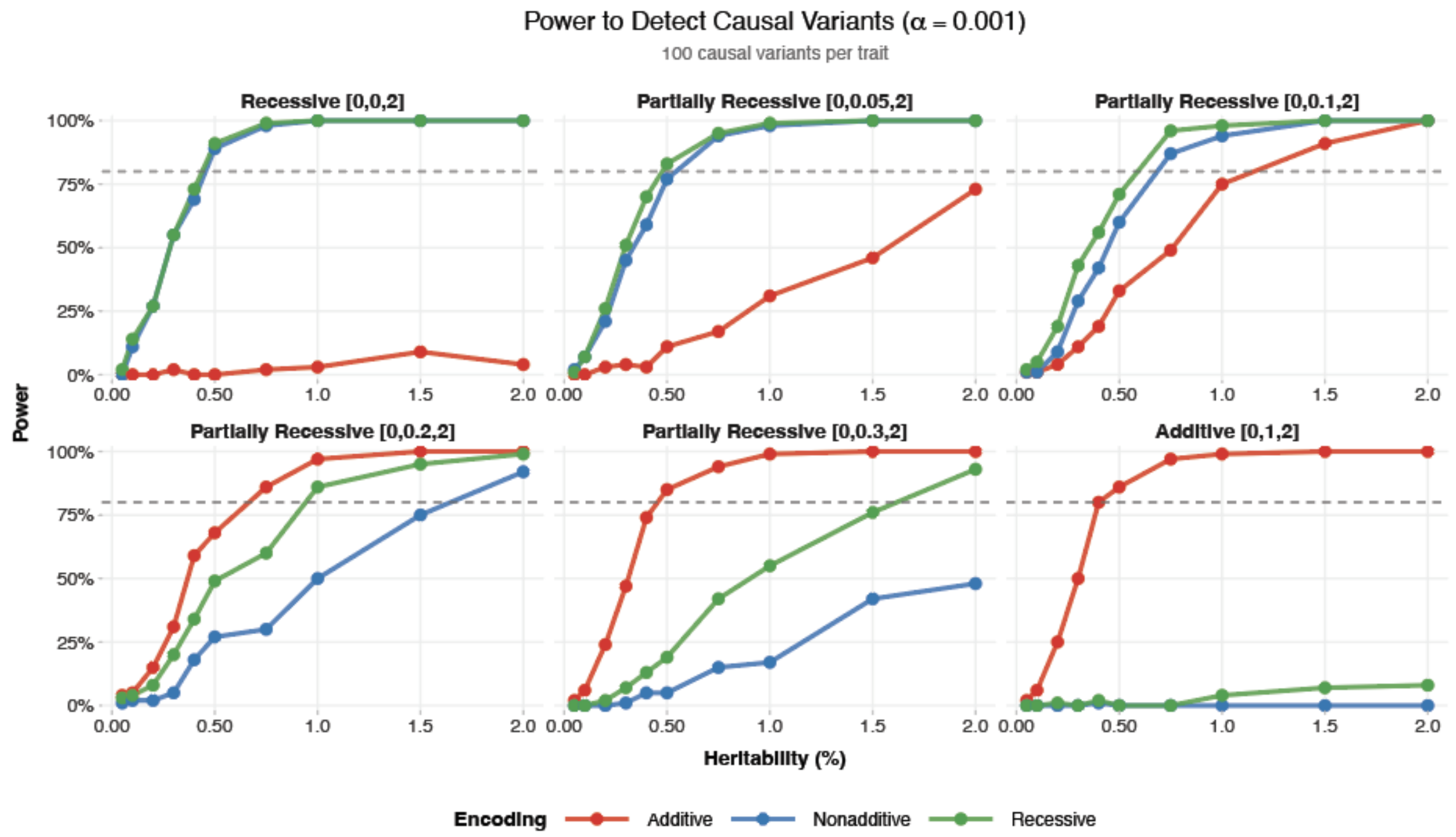

Figure R3C2A: Statistical power to detect gene-trait associations using SAIGE. Power curves for detecting associations using SAIGE linear mixed models across varying heritability levels (h^2) and genetic architectures. Phenotypes were simulated based on gene-level pseudo-variants (collapsed

rare variants), and association tests were performed using three distinct encodings: additive (red), nonadditive (blue), and recessive (green). Phenotypes were simulated by changing the heterozygote value *a* in [0, *a*, 2] ranging from *a* = 0 (recessive), *a* = 0.1 (partially recessive), to *a* = 1 (additive). The dashed horizontal line indicates 80% power.

We first simulated an additive genetic architecture to serve as a negative control. Here, the standard additive ([0, 1, 2]) allelic encoding is able to capture simulated genetic effects under the alternate hypothesis. Crucially, the nonadditive allelic recoding remained completely null with uniformly distributed P-values, confirming successful orthogonalization of the additive and nonadditive recoding (see Figures R3C2A and R3C2B).

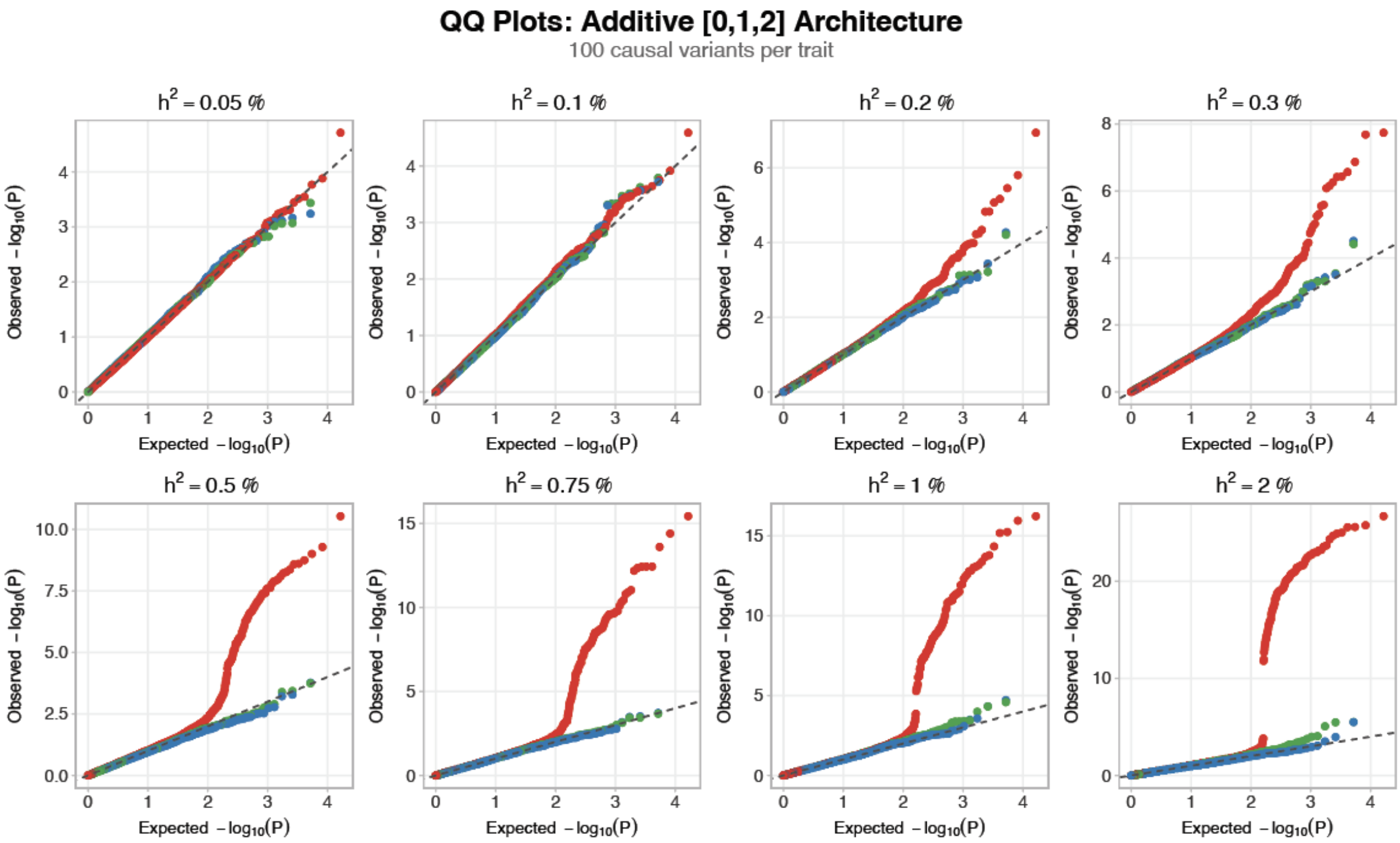

Figure R3C2B: QQ plots comparing SAIGE pseudo-variant test statistics under simulated additive and recessive architectures. QQ plots showing observed versus expected -$\log_{10}$ (P) values from SAIGE gene-level association tests. Phenotypes were simulated using causal pseudo-variants (100 variants per trait) under a true additive architecture [0, 1, 2] where heterozygotes contribute half the homozygote effect. Points are colored by the encoding used: additive (red), nonadditive (blue), and recessive (green).

When simulating a strict Mendelian recessive architecture (where the effect is only present in homozygotes), the nonadditive model demonstrated superior power compared to the additive model (Figures R3C2A and R3C2C). While the additive model can sometimes detect recessive effects (due to the correlation between additive and recessive encodings at lower MAFs), it often underestimates the effect size. Our nonadditive framework correctly identifies these as significant deviations from additivity.

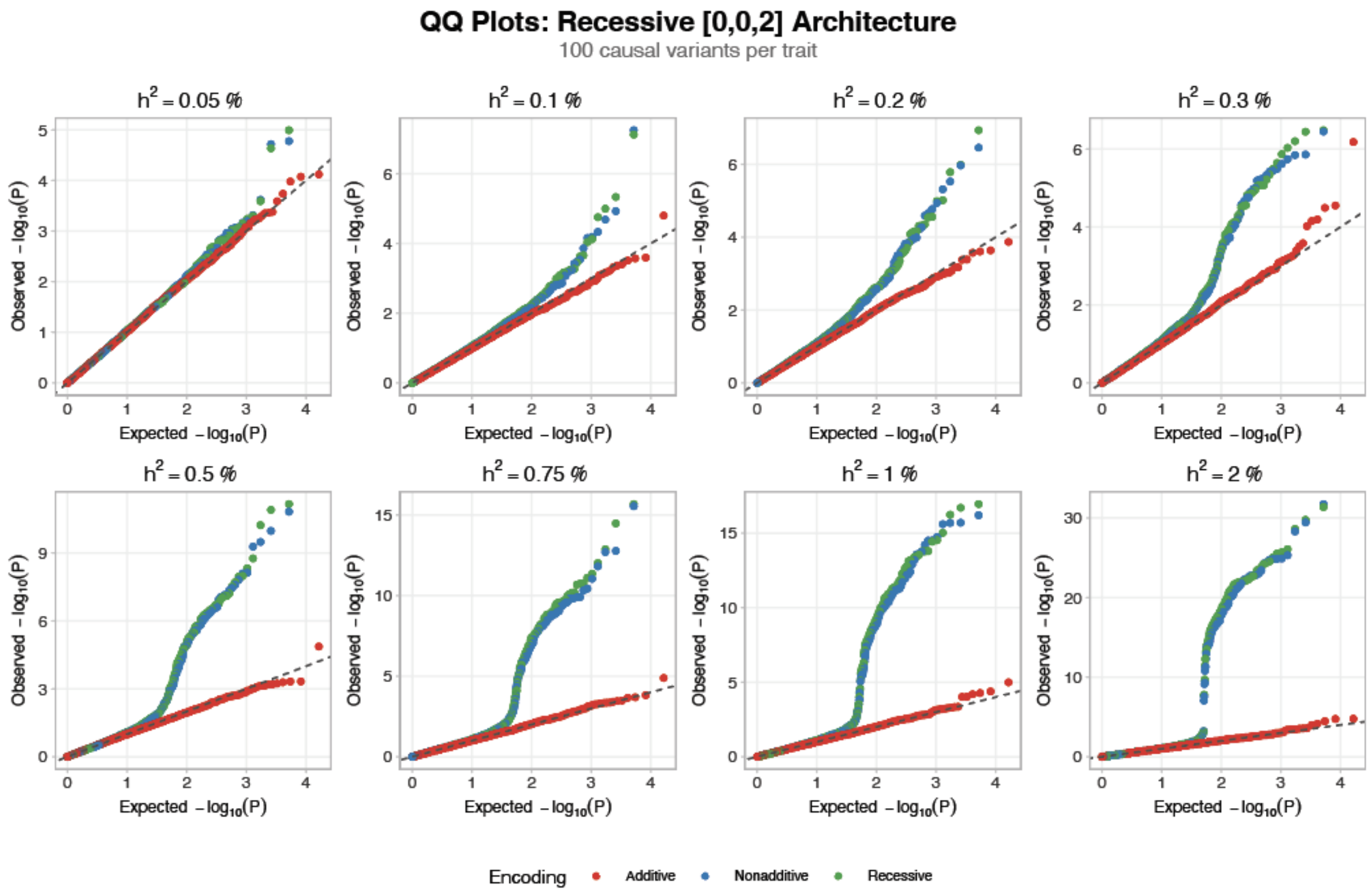

Figure R3C2C: QQ plots comparing SAIGE pseudo-variant test statistics under simulated recessive architectures. QQ plots showing observed versus expected $-\log_{10}$ (P) values from SAIGE gene-level association tests. Phenotypes were simulated using causal pseudo-variants (100 variants per trait) under a true recessive architecture [0, 0, 2] where heterozygotes have no effect. Points are colored by the encoding used: additive (red), nonadditive (blue), and recessive (green).

We then simulated partially recessive traits (also known as semi-dominance), where heterozygotes have a partial effect (e.g., 5-10% of the homozygote effect as shown here). In this scenario, the additive model often yields higher statistical power than the nonadditive model (Figures R3C2A, R3C2D, and R3C2E). Generally (and especially under HWE), heterozygous carriers vastly outnumber homozygous carriers. Consequently, the additive model aggregates the modest signal from the large number of heterozygotes, generating a strong test statistic.

We also argue that power is not the only metric of success here. Instead, characterization of the underlying mode of inheritance is equally important. By running both models, we can distinguish true linearity from partial recessivity. A significant hit in the additive model combined with a significant nonadditive result allows us to classify the locus as partially recessive, whereas using the additive model alone would risk misclassifying the biology as linear.

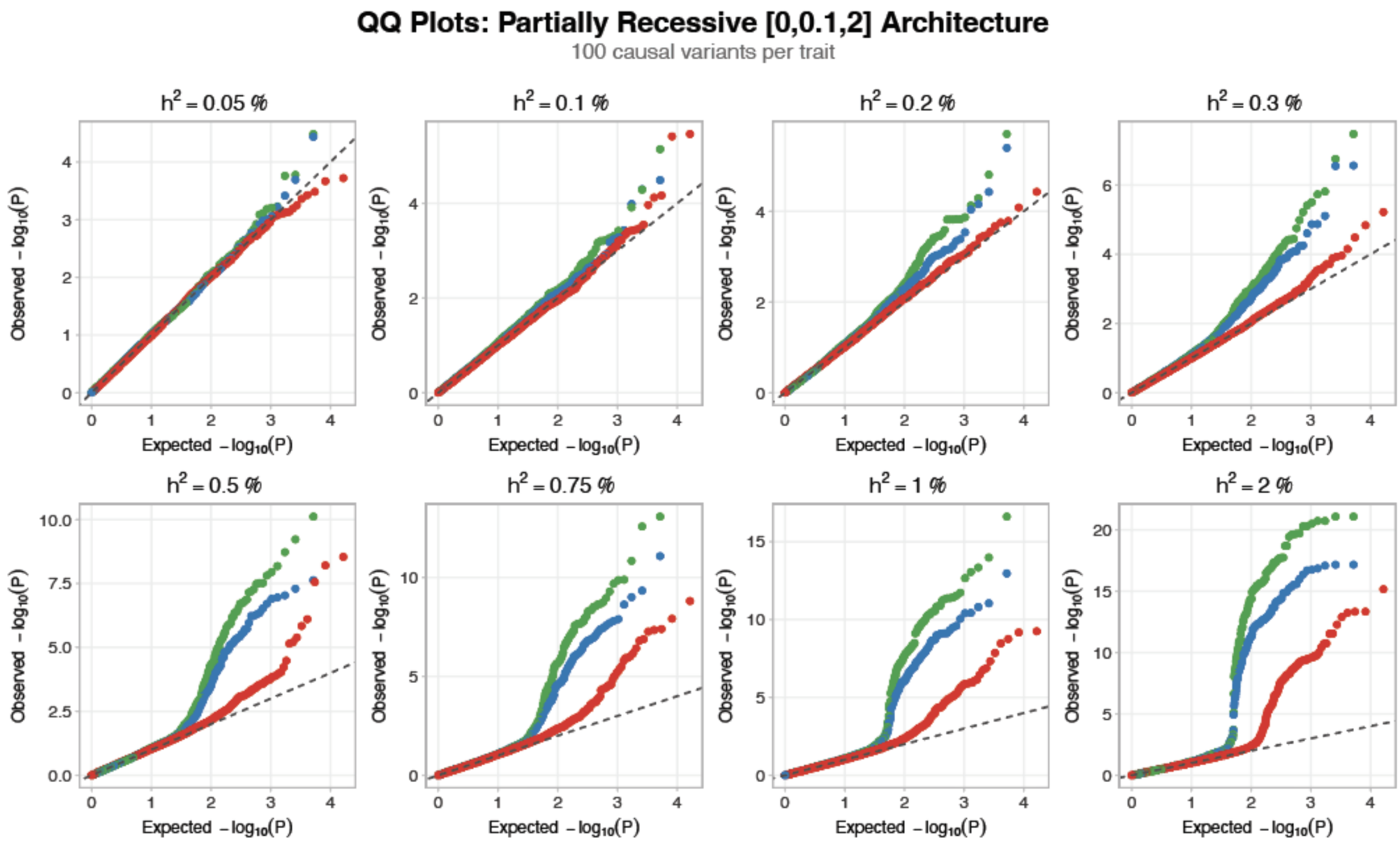

Figure R3C2D: QQ plots comparing SAIGE pseudo-variant test statistics under a strongly partially recessive architecture. The plot shows observed versus expected $-\log_{10}$ (P) values for phenotypes simulated with 100 causal variants under a [0, 0.1, 2] architecture, where heterozygotes contribute 5% of the homozygote effect. Points represent the genotype encoding used: additive (red), nonadditive (blue), and recessive (green).

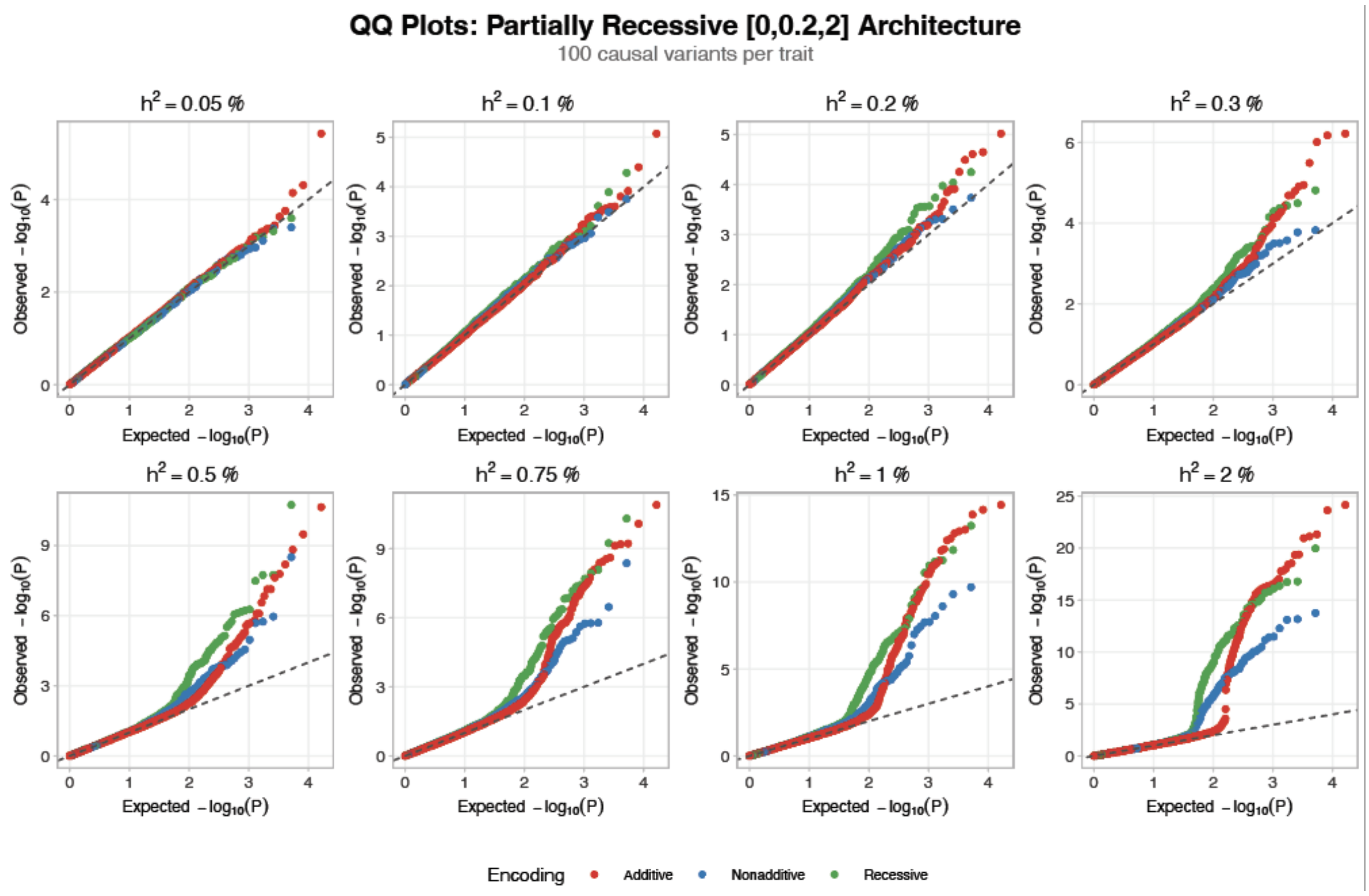

Figure R3C2E: QQ plots comparing SAIGE pseudo-variant test statistics under a moderate partially recessive architecture. The plot shows observed versus expected $-\log_{10}$ (P) values for phenotypes simulated with 100 causal variants under a [0, 0.2, 2] architecture, where heterozygotes contribute 10% of the homozygote effect. Points represent the genotype encoding used: additive (red), nonadditive (blue), and recessive (green). Despite the trait being partially recessive, the heterozygote effect is sufficiently large here that the additive model (red) performs comparably to the nonadditive and recessive models.

Together, these simulations confirm that our framework works as expected. We show that our model is able to correctly isolate the nonadditive contribution under a completely recessive mode of inheritance, and that it remains null when simulating additive genetic effects, as expected. We also confirm that we can untangle modes of partially recessive inheritance, which are otherwise contaminated under the recessive encoding due to correlation with the additive encoding, giving us the ability to find traits with more subtle deviations from additivity. **We have added Figures S8-11 to the manuscript and commented on them in Lines 129-132:**

"Using simulations, we demonstrate that this approach is well-calibrated under the null hypothesis with both SAIGE and REGENIE (Methods; Supplementary Figures S6–S7) and reliably detects true signals across diverse genetic architectures (Methods; Supplementary Figures S8–S10)"

Finally, the reviewer asked about the power of our approach in set-based tests. While our simulations use a pseudo-variant framework rather than formal set-based testing, we expect the power characteristics to generalize. The core question in both approaches is how well a given encoding captures the underlying genetic architecture: this relationship between encoding and true effect should remain consistent regardless of the specific aggregation or weighting scheme used. In both cases, the nonadditive encoding will only yield signal when there is genuine deviation from additivity, and will remain null under purely additive architectures. The key advantage of our encoding (its orthogonality to the additive term) is a property of the encoding itself, not the test framework, and thus should confer the same benefits in set-based contexts.

Importantly, we have already demonstrated that SAIGE-GENE+ maintains proper calibration with our encoding under the null (Figure R3C1E; median λ_{GC} = 1.0015 for set-based tests), providing empirical support for this expectation. Unless the reviewer feels additional power simulations in a set-based framework are essential, we believe the current analysis is sufficient to demonstrate the validity of our approach.

Other simulation scenarios to consider: While not completely necessary, other simulations that could be nice to explore involve investigating the statistical behavior of this encoding when a portion of causal variants with additive and/or effects are unobserved. Another interesting setting would be investigating the behavior of the encoding when additive and effects in the generative model are heavily correlated. Would either of these mess with the power, false discovery rate, and calibration of the approach?

We thank the reviewer for these interesting suggestions regarding the behavior of the encoding under complex generative scenarios.

Regarding the reviewer's query on correlated effects: It is important to distinguish between the correlation of the regressors (the genotype encodings) and the correlation of the true biological effects (β). While the true effects in nature may indeed be correlated (e.g., large additive effects co-occurring with large effects), the method relies on the Gram-Schmidt orthogonalization of the regressors.

A fundamental property of OLS and linear mixed models is that when regressors are orthogonal, the estimators for their coefficients are statistically independent. Consequently, even if the underlying generative effects are heavily correlated, the estimation of the nonadditive component remains unbiased and is not influenced by the magnitude of the additive component.

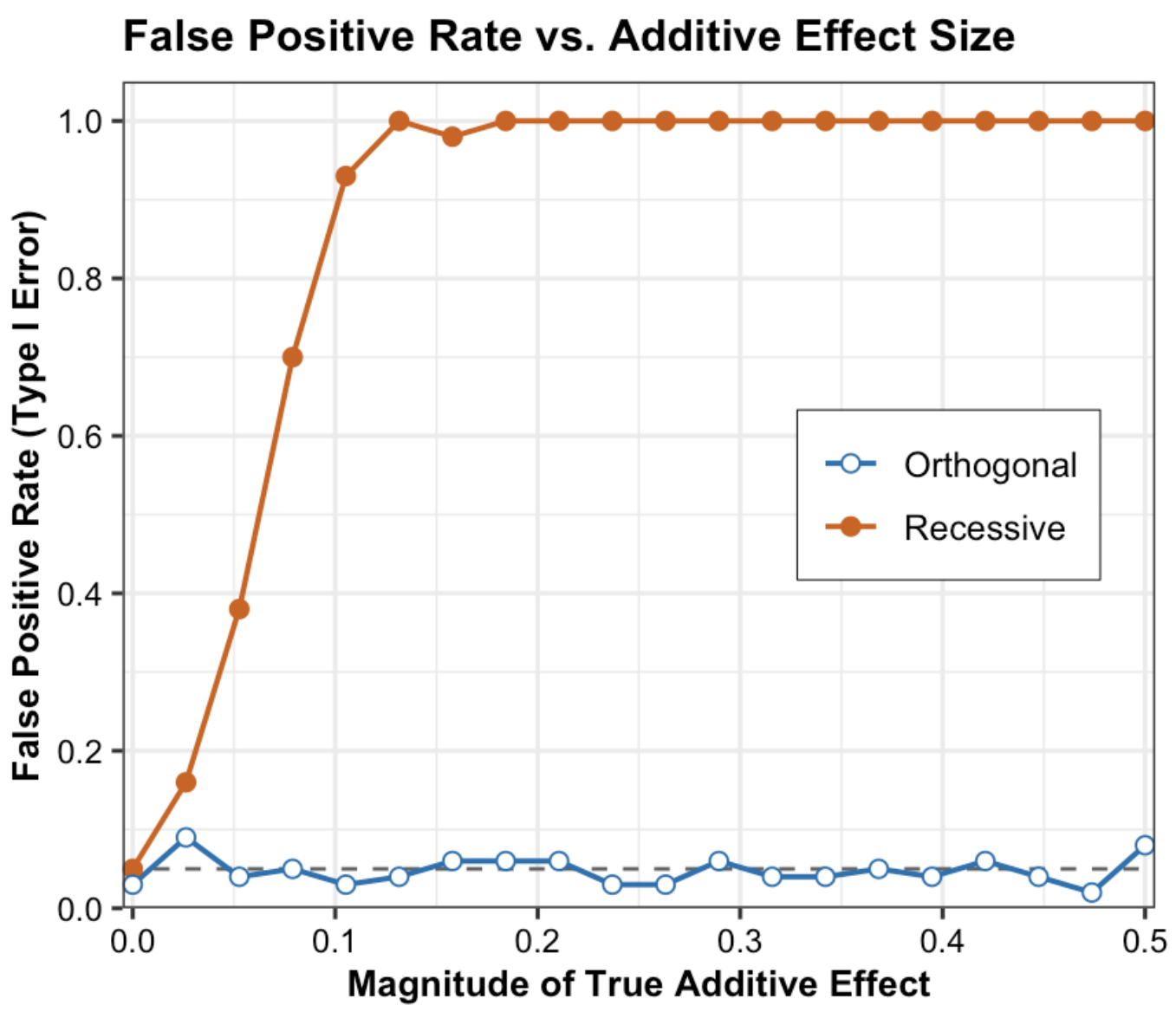

Figure R3C3A: False positive rate (Type I error) of nonadditive association tests under a generative model with strictly additive effects. The standard recessive encoding (orange) shows high error inflation ("spillover" or "bleed through") as the additive effect size increases. The orthogonal nonadditive encoding (blue) maintains the nominal Type I error rate (alpha=0.05).

To demonstrate this empirically, we performed the simulations suggested by the reviewer (the code for which are available in the github repository under "scripts/revisions"):

First, we investigated the robustness of the model to scenarios where a strong additive signal bleeds into the nonadditive test. We varied the strength of the additive effect in a scenario with zero nonadditive effect. In standard recessive models (Figure R3C3A, orange line), where encodings are correlated with the additive model, we confirmed that a strong additive effect is frequently misclassified as a recessive signal (inflated false positive rate). In contrast, our orthogonal model (blue line) maintained perfect calibration regardless of the additive effect size.

We then simulated a scenario where the generative additive and nonadditive biological effects were heavily correlated. One might intuitively worry that if genes with large additive effects also possess large nonadditive effects, the model might conflate the two. We investigated this with simulation.

Specifically, we simulated a scenario where the generative additive and nonadditive biological effects were heavily correlated (r = 0.991). As shown in Figure R3C3B, the estimated nonadditive effects align strictly with the true biological effects (y = x), even though the underlying true effects are nearly perfectly correlated. This confirms that the orthogonal decomposition successfully decouples the statistical estimation from the biological correlation, allowing us to recover unbiased estimates of nonadditivity even when they co-occur with strong additive drivers.

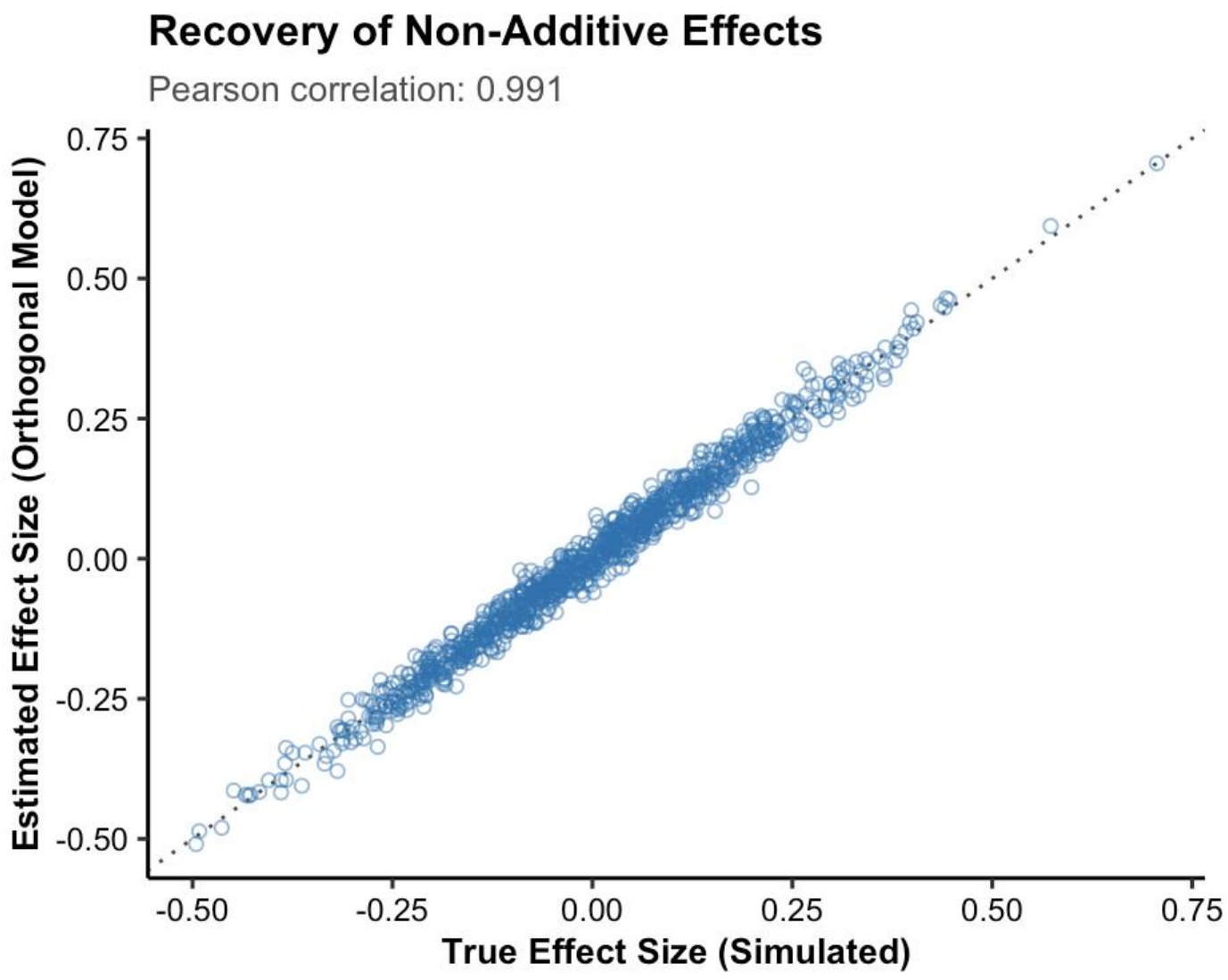

Figure R3C3B: Comparison of estimated vs. true nonadditive effects (β_D) in a simulation where the true additive and nonadditive effects are highly correlated (r = 0.991). The estimates remain unbiased (centered on the identity line), demonstrating that the orthogonal decomposition successfully isolates the nonadditive component regardless of the underlying biological correlation.

Regarding unobserved causal variants: we agree that this is a relevant scenario for all association studies. In this case, the power to detect nonadditive effects relies on the linkage disequilibrium between the observed marker and the causal variant. While power will inevitably be reduced compared to testing the causal variant directly (as is true for standard additive GWAS), the calibration (Type I error) of the orthogonal test is maintained under the null hypothesis.

Sensitivity of encoding to choice of modeling architectures: In both the Introduction (lines 88-90) and Discussion (lines 425-427), the authors make a claim that their Gram-Schmidt orthogonalization-derived recoding integrates seamlessly with existing LMM tools such as SAIGE, REGENIE, and FastGWA-GLMM. However, they only demonstrate this in SAIGE. This raises an interesting question: are these results model dependent? Since the authors

mention these approaches by name, they could show that their simulated and real data results hold with at least one of these other methods.

We thank the reviewer for this constructive comment. While our method operates at the genotype encoding level, generating standard inputs theoretically compatible with any linear modeling software that accepts non-integer dosages as input, we agree that empirical validation across different tools is essential.

To address this, we validated our findings using REGENIE. Comparing SAIGE and REGENIE head-to-head, we observed high consistency, with Z-score correlations of r = 0.906 and r = 0.932 across traits with at least one significant association in our primary analysis (see figure below). Crucially, 100% of the significant associations identified in our primary analysis were replicated: all 10 significant Olink gene-trait pairs and all 9 significant medically relevant trait associations remained significant using REGENIE.

These findings demonstrate that our results are not artifacts of a specific software implementation but are robust properties of the orthogonal encoding. **We have updated the Introduction and Discussion to reflect this validation. Additionally, we have removed the specific claim regarding FastGWA-GLMM to strictly reflect the tools explicitly tested (SAIGE and REGENIE),** though the method remains conceptually applicable to any linear modeling framework accepting standard dosage format as input.

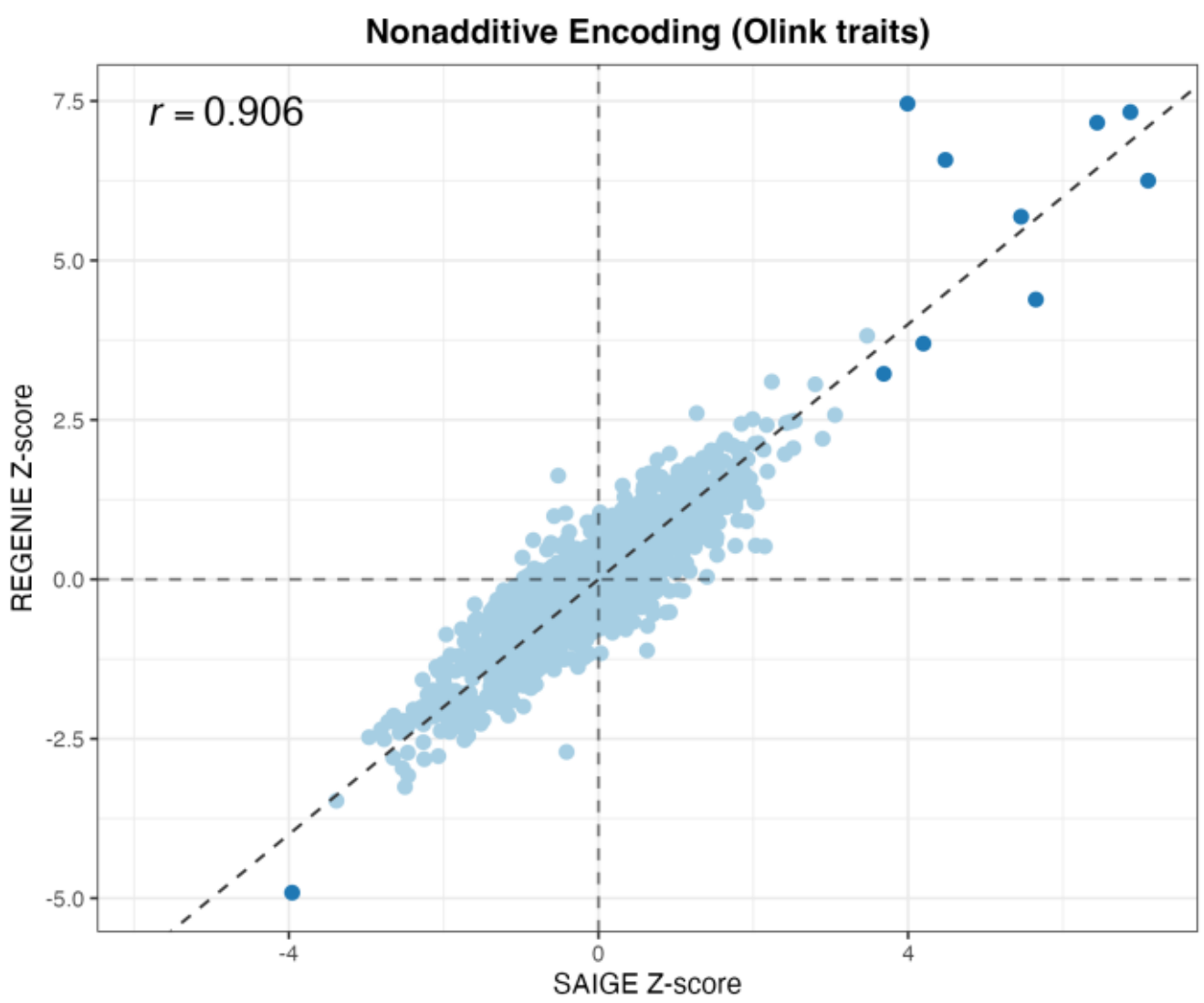

Figure R3C4: Robustness of nonadditive results across software implementations. Comparison of Z-scores derived from SAIGE (x-axis) and REGENIE (y-axis) for significant gene-trait pairs identified in the primary analysis. The strong correlation (r > 0.9) and 100% replication rate of all significant hits confirm that the nonadditive signals are consistent across different linear mixed model frameworks.

Partitioning of additive and nonadditive genetic effects: In different areas throughout the manuscript (e.g., lines 19-22, 97-99, 329-333), the authors claim that this newly proposed encoding enables the accurate additive and nonadditive heritability components at biobank scale. We might have missed this, but I don't think there are any results that back up this claim.

In addition to checking the model calibration and power, does this newly proposed encoding of nonadditive effects lead to more accurate variance component estimates? This can be checked in simulations and it would be nice if the authors could report these estimates in their real data analyses.

We thank the reviewer for this important comment. We clarify that our proposed encoding partitions genetic effects into uncorrelated additive and nonadditive components at the gene level. As such, the primary goal of this framework is to determine whether any additional signal that is not captured by the standard additive gene model (defined here as a single β multiplied by the number of affected copies), can be captured by a model that allows for deviations from additivity.

To address the reviewer's question regarding the accuracy of these estimates, we note that because our encoding is orthogonal and standardized, the effect size estimates at the gene level can indeed be used to estimate the variance explained by rare variation in that specific gene (where $h^2_{gene} = \beta^2 \times$ *sample size*). As demonstrated earlier in our simulation results (Figure R3C3B), our framework recovers these effect sizes without bias ($r > 0.99$), confirming that we can accurately quantify this local variance component.

However, we have chosen not to report a table of these variance estimates to avoid misinterpretation regarding exome-wide heritability. We are not attempting to estimate total heritability by summing these components across all genes. Doing so would be methodologically flawed because summing gene-level estimates ignores linkage disequilibrium. While the extent of LD is smaller for rare variants, tagging between genes would still result in double-counting of phenotypic variance. Furthermore, our estimates capture the variance explained by the gene-level burden of rare variation, which may not always correspond perfectly to causal variation. Therefore, while our method is accurate for detecting deviations from the simplified additive model at a specific locus, we believe it is more rigorous to restrict our reporting to these specific associations rather than providing a table of variance components that readers might incorrectly sum to infer global heritability. However, we believe that future work with larger samples and formal LD-aware methods could extend this framework to genome-wide variance partitioning.

Sensitivity to the scaling done to the phenotype: We found the choice to apply an inverse-normal transformation to all traits to be an important one. What was the reason for this choice? How does this transformation affect the findings presented in the paper? It may also be informative to include results based on normalized (but not rank-transformed) traits for comparison.

We thank the reviewer for this insightful comment regarding phenotypic transformation. Our decision to apply an inverse-normal transformation (INT) to all quantitative traits was driven by the necessity to strictly control Type I error rates. In rare variant association studies, non-normal distributions and phenotypic outliers can disproportionately influence test statistics, leading to false positives. Consequently, INT is a standard best practice for linear mixed models and is enabled by default in SAIGE to ensure residuals adhere to normality assumptions.

To confirm that our specific findings are robust to this choice and not artifacts of the rank-based transformation, we performed the requested sensitivity analysis on the significant Olink traits using standard normalization (mean 0, variance 1). Notably, 100% (10 of 10) of the associations identified in the primary Olink (pLoF) analysis remained statistically significant using standard normalization. In fact, multiple associations displayed more significant P-values when using standard normalization. This observed increase in significance arises because standard normalization preserves the magnitude of phenotypic outliers, allowing them to exert disproportionate leverage on the test statistic, whereas INT down-weights these extremes to enforce normality. This suggests that the INT approach is likely more conservative than using standardized scaling.

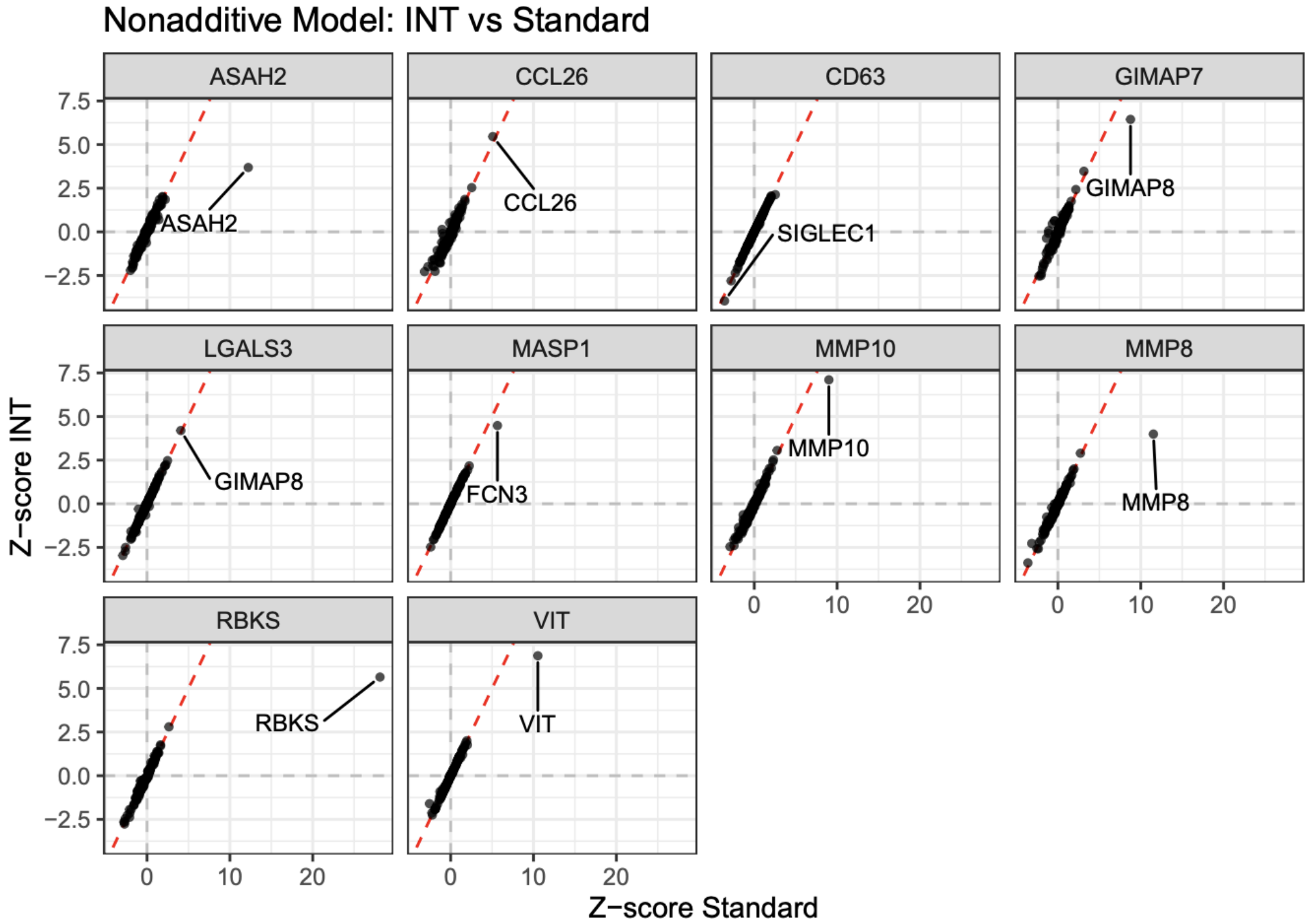

Figure R3C6A: Sensitivity analysis comparing Z-scores using inverse-normal transformation (INT) versus standard normalization. Scatter plots display the Z-scores for the significant gene-protein associations identified in the primary analysis. The x-axis represents Z-scores derived using standard phenotypic normalization (mean 0, variance 1), while the y-axis represents Z-scores from the primary rank-based INT analysis. The red dashed line indicates the identity line (y = x).

These results confirm that the nonadditive effects reported in our manuscript are driven by genuine biological signals rather than the choice of phenotypic scaling. We appreciate the reviewer's suggestion, which has helped us verify the robustness of our results. **We have added this analysis to the main text (Lines 140-143) and as Figure S11.**

Minor Comments

It would be helpful to include a clear description of how population structure was accounted for in all these analyses. For example, was this handled directly by SAIGE, by fitting the null model with a particular encoding, or by including some number of PCs? Making this explicit will help readers and reviewers evaluate the robustness of the results.

We thank the reviewer for highlighting the need for clarity regarding population structure control. We have updated the Methods section to explicitly describe our approach, which relies on a multi-layered strategy standard to mixed-model analyses. First, to minimize stratification at the cohort level, we restricted our primary analysis to individuals of European genetic ancestry (N ≈ 400,000), defined using a random forest classifier trained on 1000 Genomes data.

Second, within this subset, we accounted for fine-scale population structure and cryptic relatedness using the linear mixed model frameworks of SAIGE (and REGENIE in revised analyses). We fitted a null model using a sparse genetic relatedness matrix constructed from high-quality genotyped variants using standard additive encoding to model random effects, while including the first 10 PCs alongside age, sex, and interaction terms as fixed effects.

Importantly, our proposed framework does not alter the null modeling step (Step 1); the orthogonal recoding is applied only to the candidate variants during the association scan (Step 2). Consequently, the rigorous control for population structure provided by the null model remains fully intact.

The conclusion that one in three cognate gene-protein pairs exhibit nonlinear dose-response effects is very interesting. However, since this is based on only 24 data points, we would encourage the authors to provide an estimate of statistical uncertainty to help readers gauge confidence in this result. In connection with our earlier comments, this makes providing simulation studies even more important. It would be great to understand at what sample size one would expect to see power.

We agree that the limited number of data points (24) warrants a careful assessment of uncertainty. To quantify this, we calculated the 95% binomial confidence interval for our observed proportion (6/21 significant pairs). The resulting interval (13.2%–50.6%) confirms that nonadditive effects are likely a more common feature of the proteome (likely at least >13%) than previously appreciated.

Regarding statistical power, we point to the fundamental constraints of discovery. As detailed in Palmer et al. (2023), detecting nonadditive effects typically requires a 20- to 30-fold increase in sample size compared to additive effects for common genetic variation (MAF > 5%). For rare variants, this challenge is compounded by the "homozygote bottleneck," where the number of informative homozygous carriers scales with the square of the minor allele frequency ($N \times MAF^2$).

Crucially, even if rare variants carry larger biological effects due to negative selection, this does not overcome the geometric decline in homozygote frequency. For example, to detect a recessive effect at MAF = 0.1% with 80% power, one would need approximately 2,000 times the sample size required for an additive model, even if the effect size is large ($\beta = 2.0$). Thus, while current biobank sample sizes (N ≈ 400k) limit our discovery power to genes with higher aggregate deleterious allele frequencies, our framework is rigorously calibrated to control Type I error in this regime. We anticipate that as sample sizes increase (for instance, with the next UK Biobank Olink release), the number of identifiable carriers will grow, narrowing confidence intervals and enabling broader replication.

The threshold for distinguishing common vs. rare variants is not used consistently (sometimes MAF ≥ 0.01, sometimes ≥ 0.05). Standardizing this throughout will improve clarity.

We thank the reviewer for noting this distinction. We clarify that the use of two distinct MAF thresholds is intentional and serves specific methodological purposes.

First, regarding the aggregation threshold, we aggregate rare variants into gene-level masks using a MAF < 5% cutoff. This threshold is critical for statistical power because detecting nonadditive effects requires a sufficient number of biallelic carriers (homozygotes or compound heterozygotes); a stricter cutoff (e.g., <1%) would effectively eliminate the vast majority of testable homozygous carriers.

Second, regarding the conditioning threshold, we control for nearby independent variants down to MAF > 1%. This is a conservative measure to prevent LD "spillage" or "bleed-through" ensuring that our aggregated rare-variant signal is not falsely driven by nearby variants with intermediate frequencies (1–5%) that would be missed if we only controlled for common variants >5%.

We have updated the Methods section (Lines 890-893) to explicitly distinguish between the aggregation threshold used for mask construction and the conditioning threshold used for ensuring independence.

Figure S6: The QQ-plot axis labels and captions seem to contain contradictions. Aligning these descriptions will avoid confusion.

We thank the reviewer for finding this. We have now fixed the axis labels and captions and revised the manuscript accordingly.

Reviewer #3 (Remarks on code availability):

It appears that the code appears in different places. The C++ software to perform the recoding is available here: https://github.com/frhl/call_chets. And then the code used in the manuscript can be found on here https://github.com/frhl/nonadditivity.

It would appear that all the scripts necessary are provided in both of these resources; however, we would encourage the authors to create more detailed READMEs in both repos to better facilitate the implementation of their approach and the reproducibility of their work.

Thank you. We have updated each codebase to address the reviewer's comments:

First, we have added a README that allows other users to reliably reproduce our work, covering variant annotation, phasing, conditional analysis, set-based and variant-based analysis, phenotype construction, 2df testing, and more. We hope that this allows others to apply our analysis procedures to their data.

Second, we have made extensive updates to the C++ software so that it can be used easily. We performed a major software release (v0.4.0 → v1.0.4) with significant improvements:

1. Refactored from procedural to object-oriented design.

2. Expanded test coverage from 8 to 62 test files and added continuous integration (https://github.com/frhl/call_chets).
3. Added detailed documentation with end-to-end examples covering pseudo-variant, variant-based, phased, and unphased workflows (see /examples folder).
4. Implemented a cross-platform build system and pushed to Docker Hub (https://hub.docker.com/r/fhlassen/call_chets).
5. Restructured the codebase following C++ best practices.

These updates ensure both the analytical workflows and software tools are accessible and reproducible for the research community.

Reviewer #4 (Remarks to the Author):

I co-reviewed this manuscript with one of the reviewers who provided the listed reports. This is part of the Nature Communications initiative to facilitate training in peer review and to provide appropriate recognition for Early Career Researchers who co-review manuscripts.

We thank the reviewer for taking the time to review our manuscript.
