# Supplementary material for: Deviations from genetic additivity driven by rare variants at biobank scale
Source: Nat Commun. 2026 Aug 29;17:10302. doi: 10.1038/s41467-026-76151-w (PMC13620152; doi:10.1038/s41467-026-76151-w)
Supplement: Supplementary file 3 [file 41467_2026_76151_MOESM1_ESM.pdf]

Supplementary information for Deviations from genetic additivity driven by rare variants at biobank scale

Frederik H. Lassen[1,2,*], Samvida S. Venkatesh[3], Nikolas A. Baya[1,2,3],
Cecilia M. Lindgren[1,2,4] and Duncan S. Palmer[2,3,5,6,*]

*Correspondence to: duncan.palmer@stats.ox.ac.uk and frederik.lassen@oncology.ox.ac.uk

This file includes:

- Supplementary Notes 1–4
- Supplementary Figures 1–19 and 20

Supplementary Notes

Supplementary Note 1: Full derivation of nonadditive genotype transformation

We consider the general case in which the only restriction is that the sum of proportions is 1:

$$\begin{bmatrix} r \\ h \\ a \end{bmatrix}, \text{ such that } r + h + a = 1. \tag{1}$$

We define:

$$X^{0\prime} = \begin{bmatrix} 1 \\ 1 \\ 1 \end{bmatrix}, \; X^{A\prime} = \begin{bmatrix} 0 \\ 1 \\ 2 \end{bmatrix}, \; X^{D\prime} = \begin{bmatrix} 0 \\ 1 \\ 0 \end{bmatrix}. \tag{2}$$

We define an inner product according to the general genotype proportions:

$$\langle X, Y \rangle = rX_0Y_0 + hX_1Y_1 + aX_2Y_2. \tag{3}$$

Under this encoding, we then determine the additive component (equivalent to centering and scaling the additive encoding assuming the genotype proportions) using Gram-Schmidt:

$$X^0 = \frac{1}{\|X^{0\prime}\|} \begin{bmatrix} 1 \\ 1 \\ 1 \end{bmatrix} = \begin{bmatrix} 1 \\ 1 \\ 1 \end{bmatrix}, \tag{4}$$

$$X^A = \frac{X^{A\prime} - \langle X^{A\prime}, X^0 \rangle X^0}{\|X^{A\prime} - \langle X^{A\prime}, X^0 \rangle X^0\|} = \frac{1}{\sqrt{-h^2 - 4ha + h - 4a^2 + 4a}} \left(\begin{bmatrix} 0 \\ 1 \\ 2 \end{bmatrix} - (h + 2a) \begin{bmatrix} 1 \\ 1 \\ 1 \end{bmatrix} \right) \tag{5}$$

$$= \frac{1}{\sqrt{4ar + h(a+r)}} \begin{bmatrix} -(h+2a) \\ 1 - (h+2a) \\ 2 - (h+2a) \end{bmatrix} \tag{6}$$

$$= \frac{1}{\sqrt{a + r - (a-r)^2}} \begin{bmatrix} -(h+2a) \\ 1 - (h+2a) \\ 2 - (h+2a) \end{bmatrix}. \tag{7}$$

Finally, determine the dominance encoding without assuming Hardy-Weinberg equilibrium

(HWE) by completing the basis:

$$X^D = \frac{X^{D'} - \langle X^{D'}, X^0 \rangle X^0 - \langle X^{D'}, X^A \rangle X^A}{\left\| X^{D'} - \langle X^{D'}, X^0 \rangle X^0 - \langle X^{D'}, X^A \rangle X^A \right\|} \tag{8}$$

$$= \frac{\frac{1}{(h^2+4ha-h+4a^2-4a)} \begin{bmatrix} 2ha \\ 4a(h+a-1) \\ -2h(h+a-1) \end{bmatrix}}{\left\| X^{D'} - \langle X^{D'}, X^0 \rangle X^0 - \langle X^{D'}, X^A \rangle X^A \right\|} \tag{9}$$

$$= \frac{\frac{1}{(h^2+4ha-h+4a^2-4a)} \begin{bmatrix} 2ha \\ -4ar \\ 2hr \end{bmatrix}}{2\sqrt{\frac{har}{-(h^2+4ha-h+4a^2-4a)}}} \tag{10}$$

$$= \frac{1}{\sqrt{-har(h^2+4ha-h+4a^2-4a)}} \begin{bmatrix} -ha \\ 2ar \\ -hr \end{bmatrix} \tag{11}$$

$$= \frac{1}{\sqrt{har(4ar+h(a+r))}} \begin{bmatrix} -ha \\ 2ar \\ -hr \end{bmatrix} \tag{12}$$

$$= \frac{1}{\sqrt{har(a+r-(a-r)^2)}} \begin{bmatrix} -ha \\ 2ar \\ -hr \end{bmatrix}. \tag{13}$$

Supplementary Note 2: Confirmation of HWE encoding recovery

To confirm that substitution recovers the HWE encoding, we substitute the HWE proportions into the general case equations. Under HWE:

$$
\begin{aligned}
r &= p^2 \text{ (homozygous reference)} \\
h &= 2pq = 2p(1-p) \text{ (heterozygous)} \\
a &= q^2 = (1-p)^2 \text{ (homozygous alternate)},
\end{aligned}
$$

where p is the frequency of the reference allele, and $q = 1 - p$ is the frequency of the alternate allele. We have X^D as defined previously:

$$X^D = \frac{1}{\sqrt{har(a + r - (a - r)^2)}} \begin{bmatrix} -ha \\ 2ar \\ -hr \end{bmatrix}. \tag{14}$$

We substitute the HWE proportions:

$$
\begin{aligned}
X^D &= \frac{1}{\sqrt{(2pq)(q^2)(p^2)(2pq)}} \begin{bmatrix} -2pq \cdot q^2 \\ 2p^2 \cdot q^2 \\ -2pq \cdot p^2 \end{bmatrix} \\
&= \frac{1}{2p^2q^2} \begin{bmatrix} -2pq^3 \\ 2p^2q^2 \\ -2p^3q \end{bmatrix} \\
&= \begin{bmatrix} -q^2/p \\ 1 \\ -p^2/q \end{bmatrix}
\end{aligned}
$$

We simplify this further by substituting $q = 1 - p$:

$$
\begin{aligned}
X^D &= \begin{bmatrix} -(1-p)^2/p \\ 1 \\ -p^2/(1-p) \end{bmatrix} \\
&= \begin{bmatrix} -p/(1-p) \\ 1 \\ -(1-p)/p \end{bmatrix}.
\end{aligned}
$$

This matches the expected HWE encoding for the dominance component: $[-p/(1-p), 1, -(1-p)/p]$ reported elsewhere, and we are done. Thus, we confirm that substituting the HWE proportions into the general case equations does indeed recover the expected HWE encoding for the dominance component.

Supplementary Note 3: Set-based analysis of rare variant nonadditive effects

In most large-scale rare variant analyses, phasing haplotypes and modeling gene-traits as individual pseudo-variants are not feasible. Instead, additive gene-based rare variant burden models are employed to enhance statistical power, using tools such as `SAIGE-GENE+`[1]. To demonstrate the application of our approach in such a framework, we applied the nonadditive recoding to individual variants, rather than gene-based pseudo-variants, and modelled them using `SAIGE-GENE+` burden testing. We focused on the traits that demonstrated at least one significant nonadditive association in the pseudo-variant analysis. We assessed the combined burden of putative loss of function (pLoF) and damaging missense/protein-altering variant categories, mirroring our previous analysis. We also restricted the analysis to the same putatively well-powered gene-trait pairs examined in the pseudo-variant approach with at least five biallelic carriers.

Our analysis exhibited well-calibrated type 1 errors ($\lambda_{GC} = 1.02$) across gene-level variation associations (Figure 20a). Only 5 of the 11 (45%) gene-trait associations that were significant in the pseudo-variant analysis reached significance in the nonadditive burden analysis (Bonferroni $P < 3.99 \times 10^{-7}$). To elucidate the factors driving this discrepancy, we conducted a direct comparison of P-values between the two analyses (Figure 20b). We found that 7 of 11 (63%) associations yielded more significant P-values in the pseudo-variant analysis. This observation suggested two potential explanations: 1) the phased information contributed significantly to detecting these associations; and 2) ultra-rare variants (minor allele count (MAC)$\leq$ 10), which were included in our pseudo-variant analysis but excluded from the variant-based analysis, could be driving some of these gene-trait associations. The validity of these hypotheses was supported by the relationship between deviation from the $x = y$ line and the proportion of compound heterozygote (compound heterozygous (CH)) identifications illustrated in the figure. For instance, the *FLG* associations, significant only in the pseudo-variant analysis, showed a high proportion of CH carriers (66%, with 1128 CH and 476 homozygous carriers). This also explains why the *FLG*-eosinophil count association did not replicate in the All of Us (AoU) cohort: the set-based analysis excludes compound heterozygotes and considers only homozygous carriers, yielding a non-significant signal in both UK Biobank (UKBB) ($P = 0.002$) and AoU. In contrast, the *BTNL9*-HDL association, which was significant in both analyses, had a much lower proportion of CH carriers (11%, with 9 CHs and 82 homozygotes). This demonstrates that the nonadditive encoding can be successfully applied within a standard gene-based burden framework to systematically search for nonadditive effects. This nonadditive burden approach complements existing analyses that lack phased data. The observed differences in significance and the relationship with CH proportions underscore the potential importance of phased information and rare variants in detecting nonadditive gene-trait associations.

Supplementary Note 4: Additive filtering enriches for biologically plausible nonadditive effects

We employed a two-stage approach where we first identified gene-protein pairs with significant

additive effects, then tested for nonadditive effects within this subset. We undertook this strategy because loci that exhibit nonadditive effects tend to also exhibit additive signals at sufficient minor allele frequency (MAF). This is because biologically plausible effect patterns typically follow $\beta \times [0, a, 2]$ where $0 < a < 2$, encompassing additive ($a = 1$), partially recessive ($0 < a < 1$), and partially dominant ($1 < a < 2$) inheritance modes, all of which contain additive components enabling initial detection. In contrast, pure nonadditive effects without any additive contribution (e.g., $\beta \times [0, 1, -N]$ where N is large) are less plausible, as they would require heterozygotes to exhibit dramatically different phenotypes than both homozygous states without any monotonic relationship to allele dosage. Even classical recessive disorders may display some gene dosage sensitivity or haploinsufficiency effects in heterozygotes[2–4], creating detectable additive signals alongside the primary nonadditive effect. By filtering to loci with significant additive effects, we substantially reduce the multiple testing burden while enriching for biologically relevant associations, then apply appropriate correction for nonadditive testing within this subset. This approach maximizes statistical power while leveraging the biological expectation that nonadditive effects predominantly occur within the context of detectable additive effects. However, this approach may fail when the MAF is low and when the mode of inheritance is strictly recessive.

Supplementary Figures

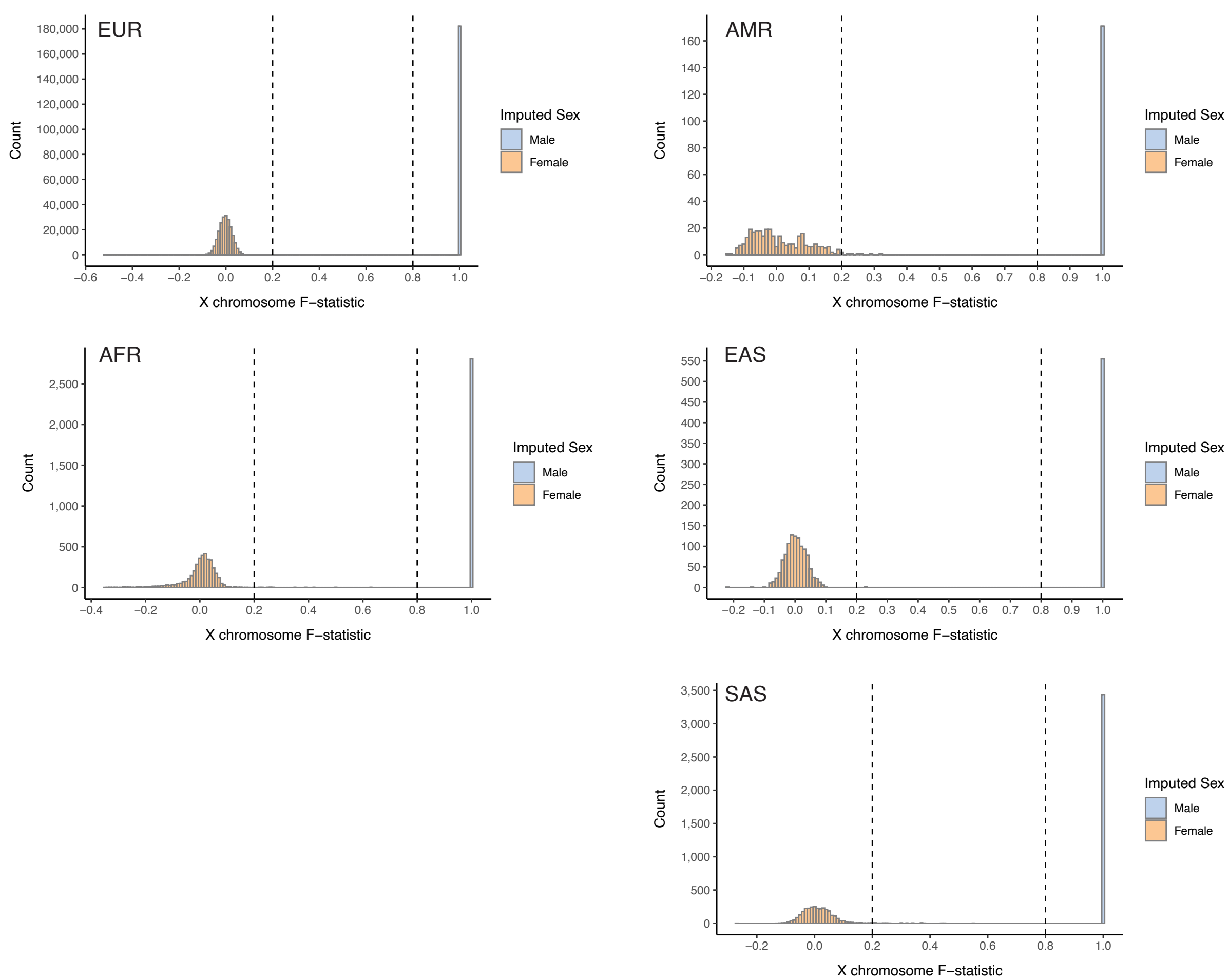

Supplementary Figure 1: Overview of sex imputation for 450k exome sequencing data. Sex imputation in UKBB participants using X chromosome variants. The F-statistic, derived from a pruned set of X chromosome variants, is used to impute sex across different ancestries. The dashed line (F-statistic = 0.6) represents the threshold for sex classification: values above indicate males, below indicate females.

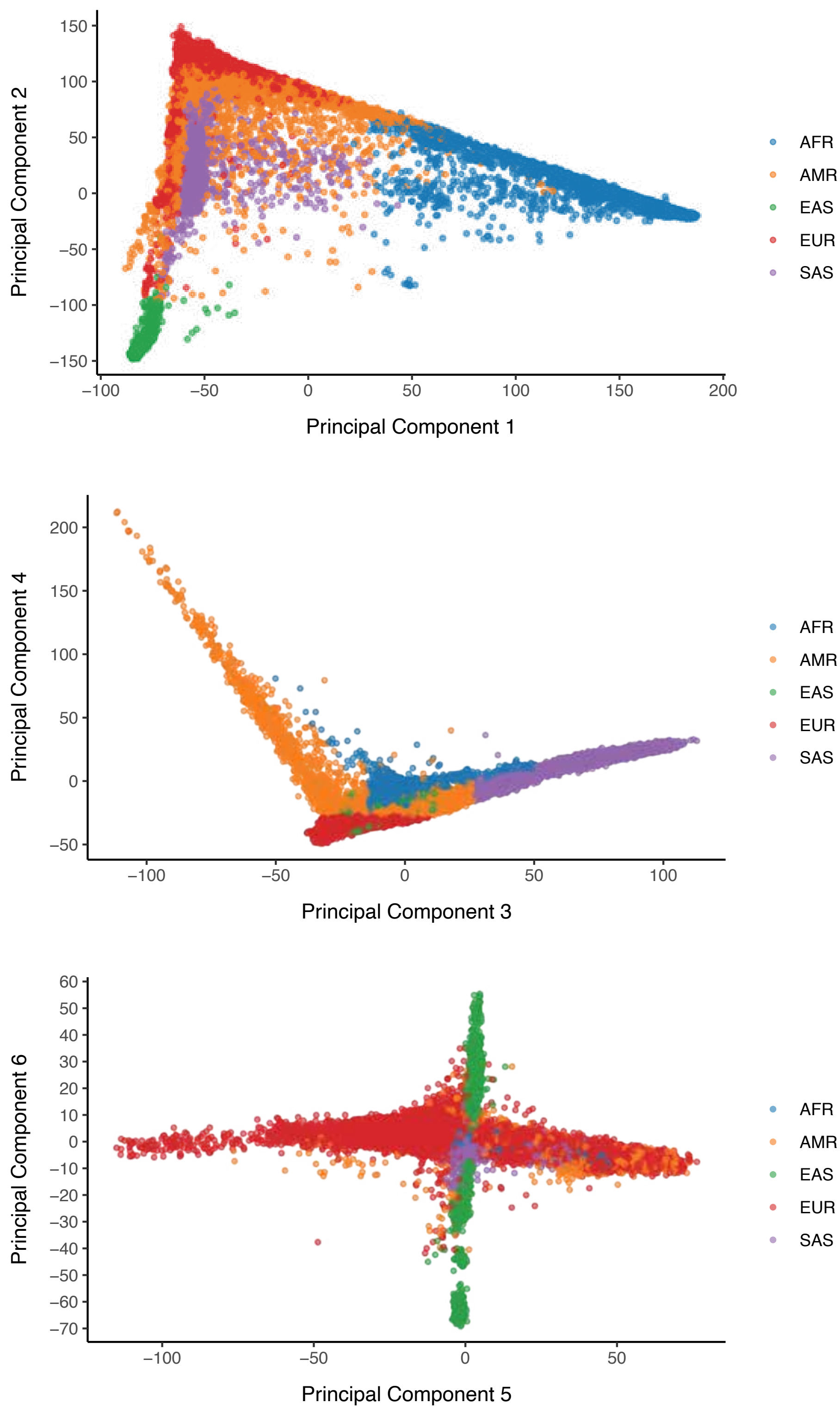

Supplementary Figure 2: Principal component analysis with 1000 genomes project for 450k exome sequencing data. Principal component analysis of genetic variation using 1000 Genomes data[5]. The first six principal components are shown, derived from a pruned set of variants. Colors represent predicted ancestries assigned by a random forest (RF) classifier.

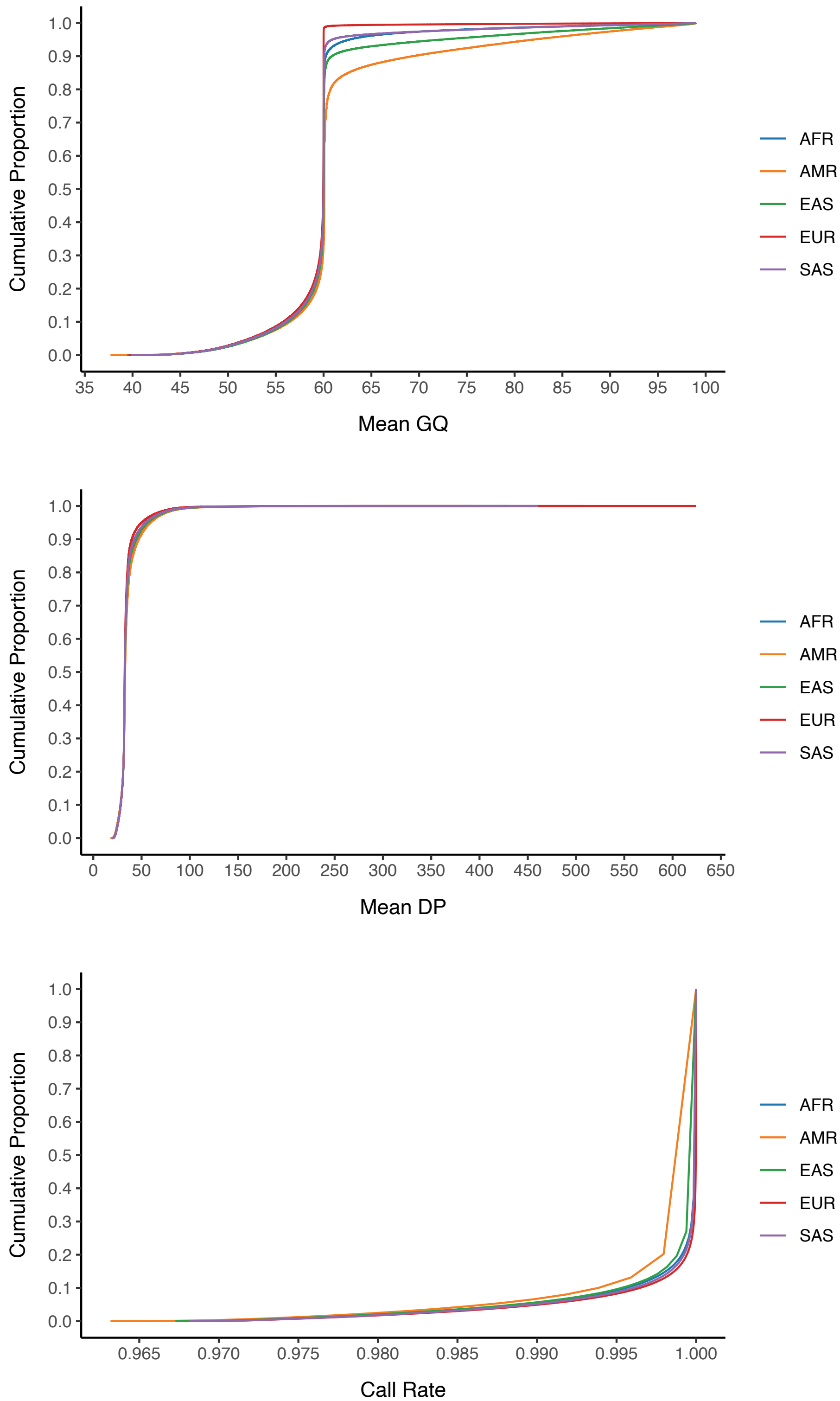

Supplementary Figure 3: Mean genotype quality, allelic depth and call rate for 450k exome sequencing data. Cumulative distributions of key sequencing quality metrics across major ancestry groups. From top to bottom, the panels show: mean genotype quality (GQ), mean sequencing depth (DP), and call rate. Each colored line represents a distinct major ancestry group.

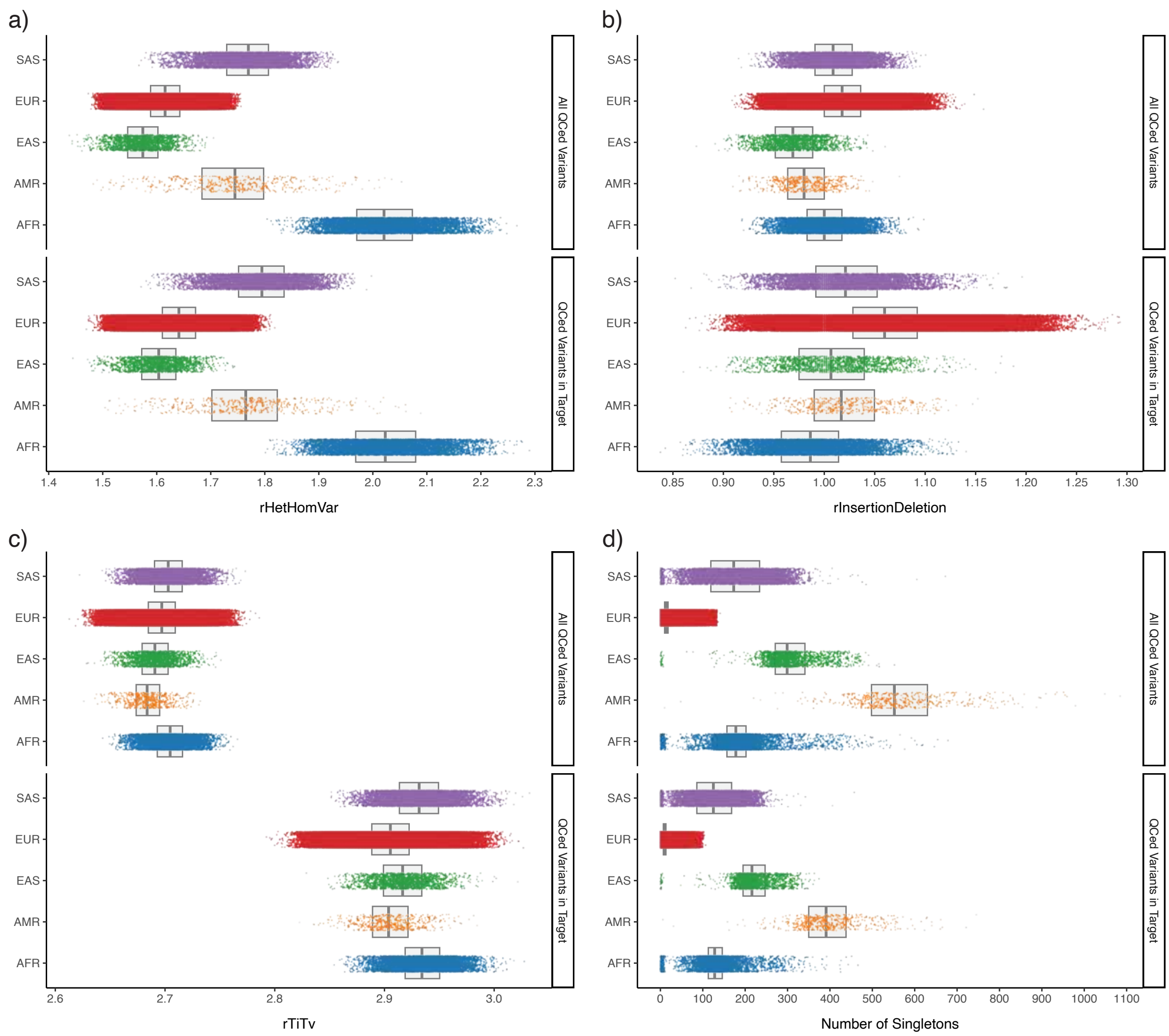

Supplementary Figure 4: Variant metrics before and after sample removals for 450k exome sequencing data. Distribution of key genomic metrics before and after sample removal. Each panel illustrates: a) ratio of heterozygous to homozygous variants ($r_{\text{HetHomVar}}$); b) ratio of insertions to deletions ($r_{\text{InsertionDeletion}}$); c) ratio of transitions to transversions (r_{TiTv}); and d) number of singletons. Upper and lower parts of each panel represent distributions before and after sample removal, respectively. Center line, median; box limits, upper and lower quartiles; whiskers, 1.5× interquartile range.

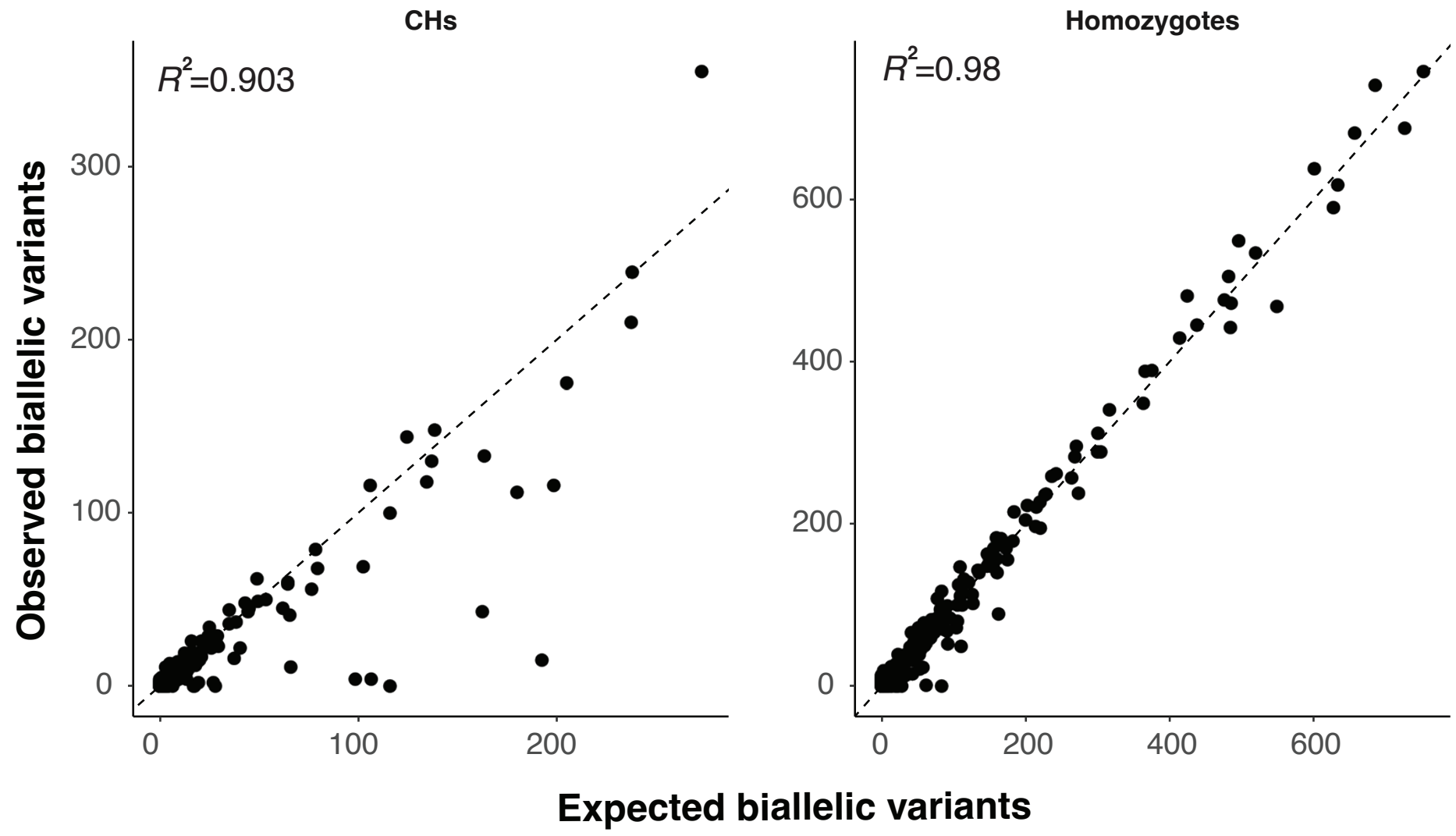

Supplementary Figure 5: Comparison between simulated and empirically observed counts of pLoF carriers. Observed versus expected CH (left) and homozygous (right) pLoF events in 339,041 unrelated Europeans from UKBB. Each dot represents a gene. X-axis shows expected events; Y-axis shows observed events. Coefficient of determination (R^2) is displayed in the top left corner of each plot.

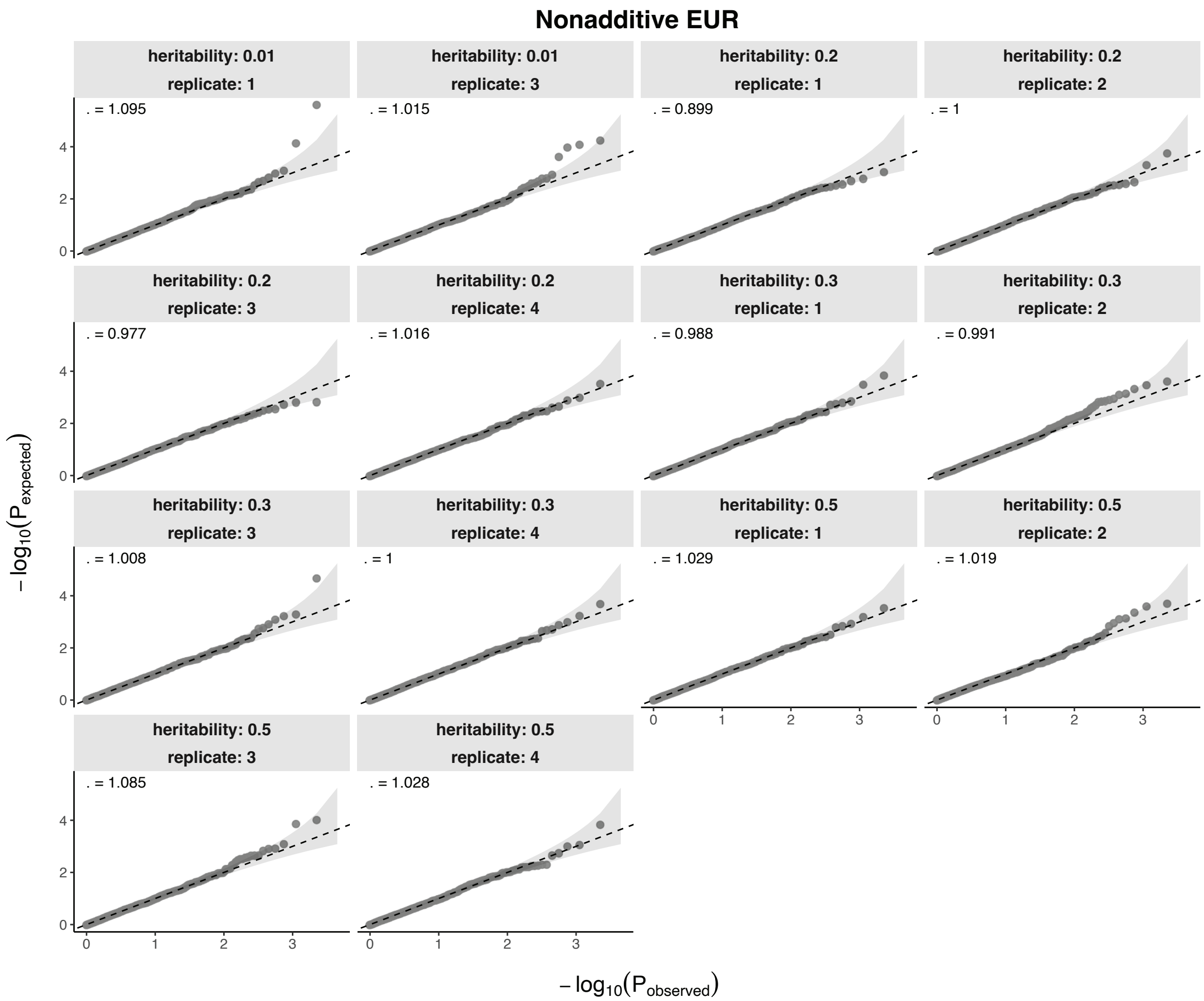

Supplementary Figure 6: QQ-plot of P-values simulated under the null hypothesis using `SAIGE`. The plot compares the observed distribution of P-values (x-axis) from nonadditive association tests against the expected uniform distribution under the null (y-axis). The data points closely follow the diagonal line with no significant deviation (median $\lambda_{GC} \approx 1$), demonstrating that the test statistics are well-calibrated and the method effectively controls type I error rates for nonadditive terms.

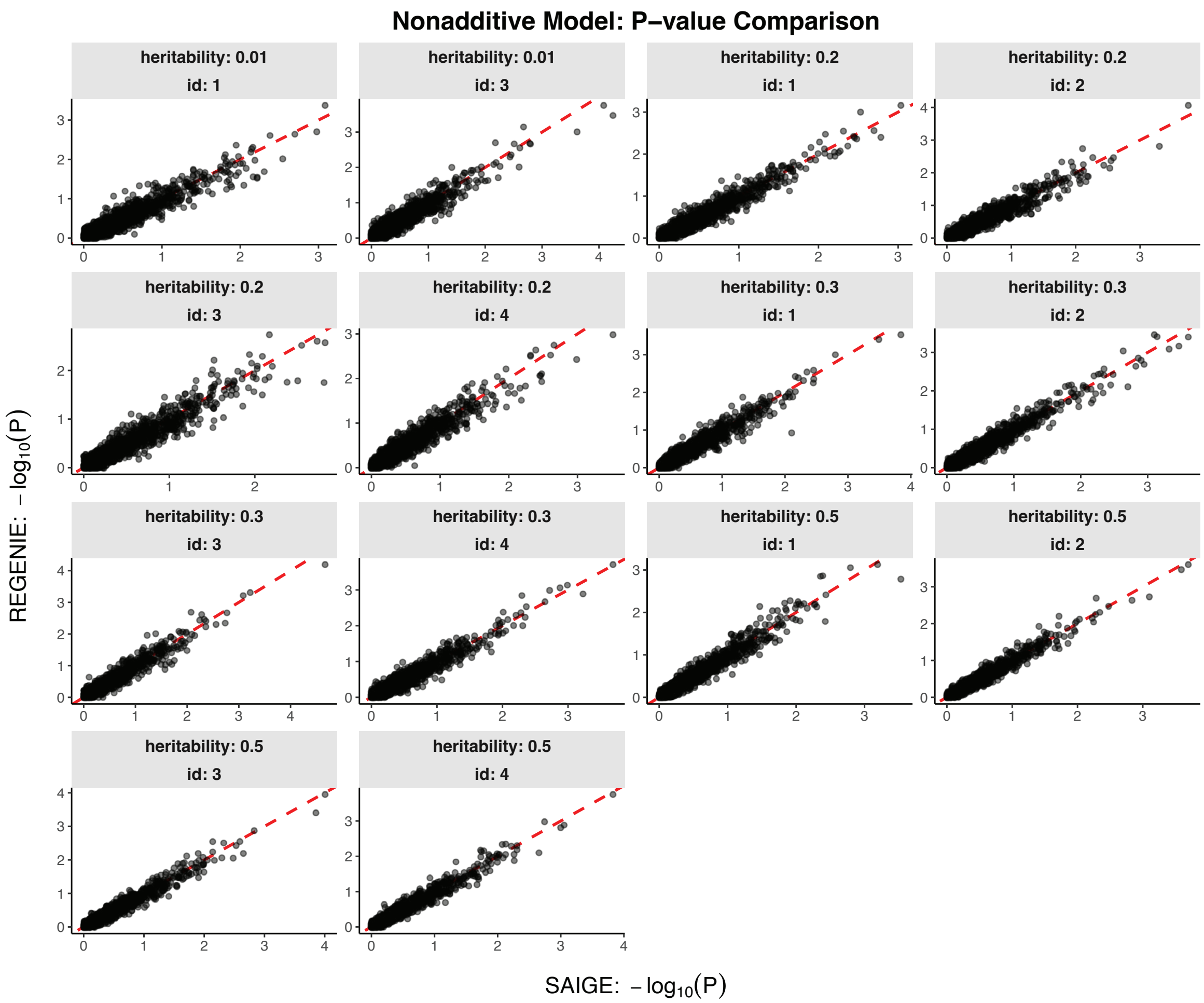

Supplementary Figure 7: Comparison of REGENIE and SAIGE association statistics for traits simulated under the null. Scatter plot comparing nonadditive P-values ($-\log_{10}$ scale) generated by `REGENIE` (y-axis) and `SAIGE` (x-axis). The strong linear correlation demonstrates that both mixed-model frameworks handle the orthogonalized nonadditive encoding consistently, yielding nearly identical test statistics in a null simulation setting.

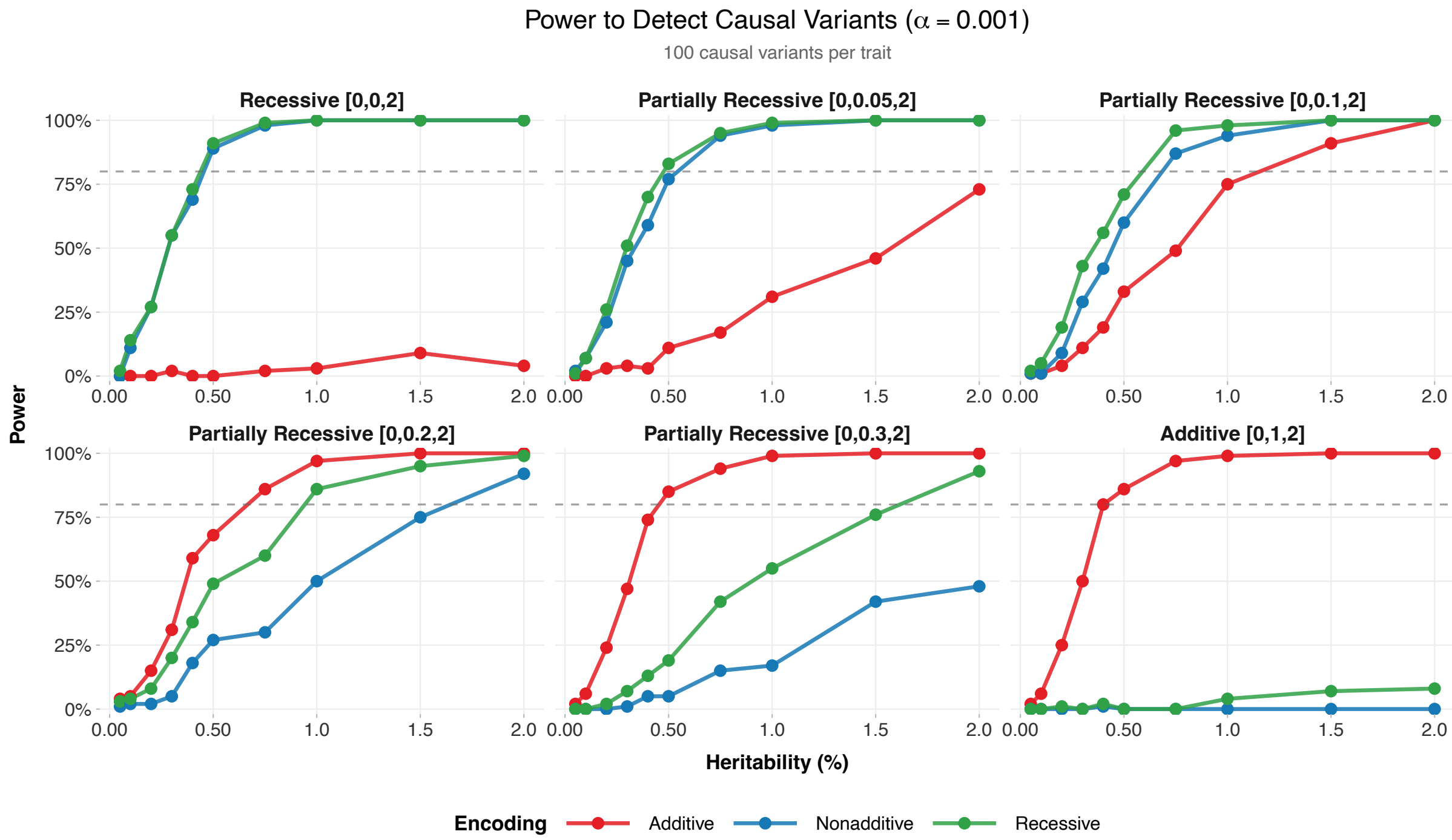

Supplementary Figure 8: Statistical power to detect gene-trait associations using SAIGE. Power curves for detecting associations using SAIGE linear mixed models across varying heritability levels (h^2) and genetic architectures. Phenotypes were simulated based on gene-level pseudo-variants (collapsed rare variants), and association tests were performed using three distinct encodings: additive (red), nonadditive (blue), and recessive (green). Phenotypes were simulated by changing the heterozygote value a in $[0, a, 2]$ ranging from $a = 0$ (recessive), $a = 0.1$ (partially recessive), to $a = 1$ (additive). The dashed horizontal line indicates 80% power. Analyses were performed in $\sim 400,000$ UK Biobank European ancestry individuals treating genes as pseudo-variants.

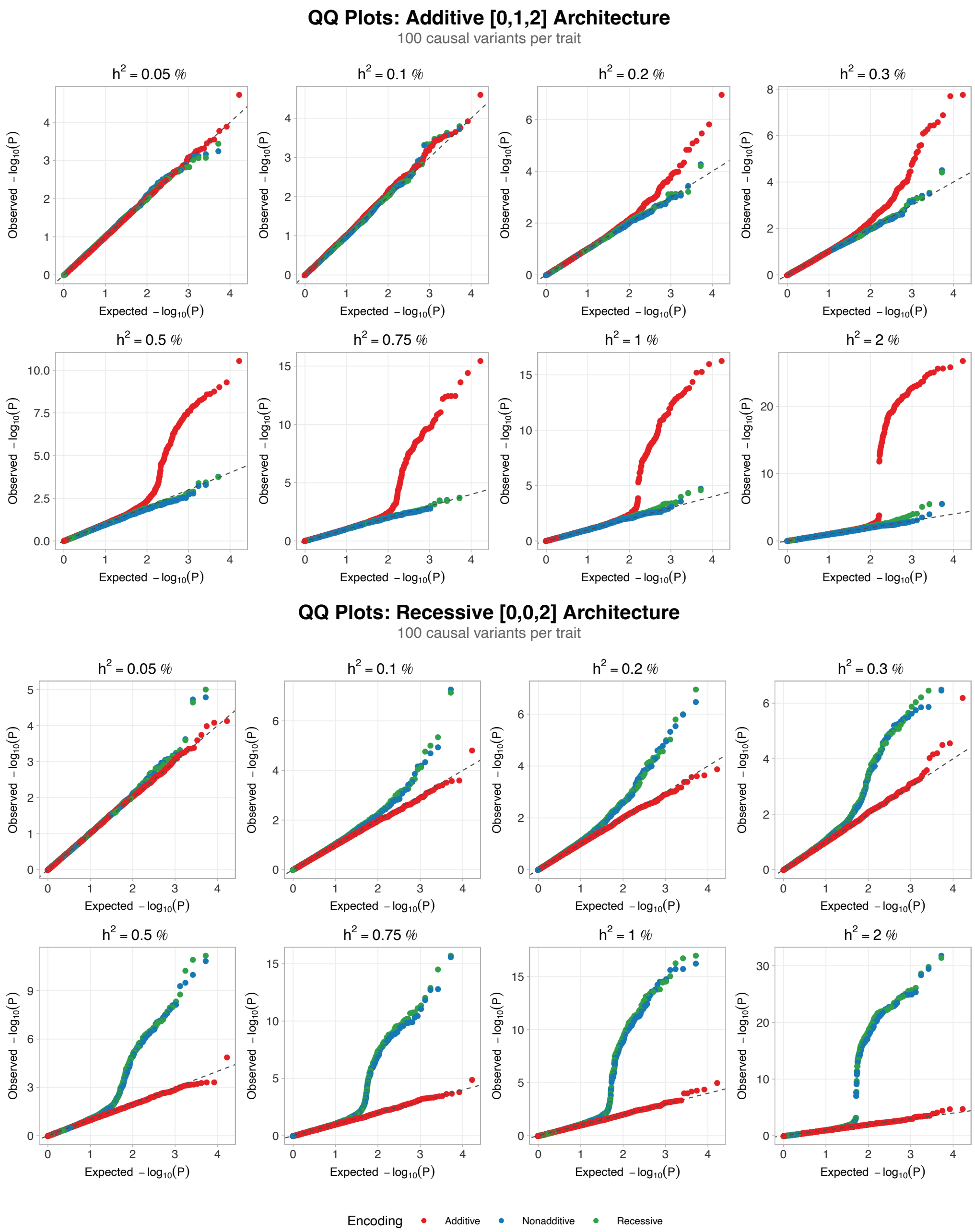

Supplementary Figure 9: QQ-plots comparing SAIGE pseudo-variant test statistics under simulated additive and recessive architectures. QQ-plots showing observed versus expected $-\log_{10}(P)$ values from SAIGE gene-level association tests. Phenotypes were simulated using causal pseudo-variants (100 variants per trait) under two architectures: (Top) true additive architecture $[0, 1, 2]$ where heterozygotes contribute half the homozygote effect; and (Bottom) true recessive architecture $[0, 0, 2]$ where heterozygotes have no effect. Points are colored by the specific encoding used: additive (red), nonadditive (blue), and recessive (green).

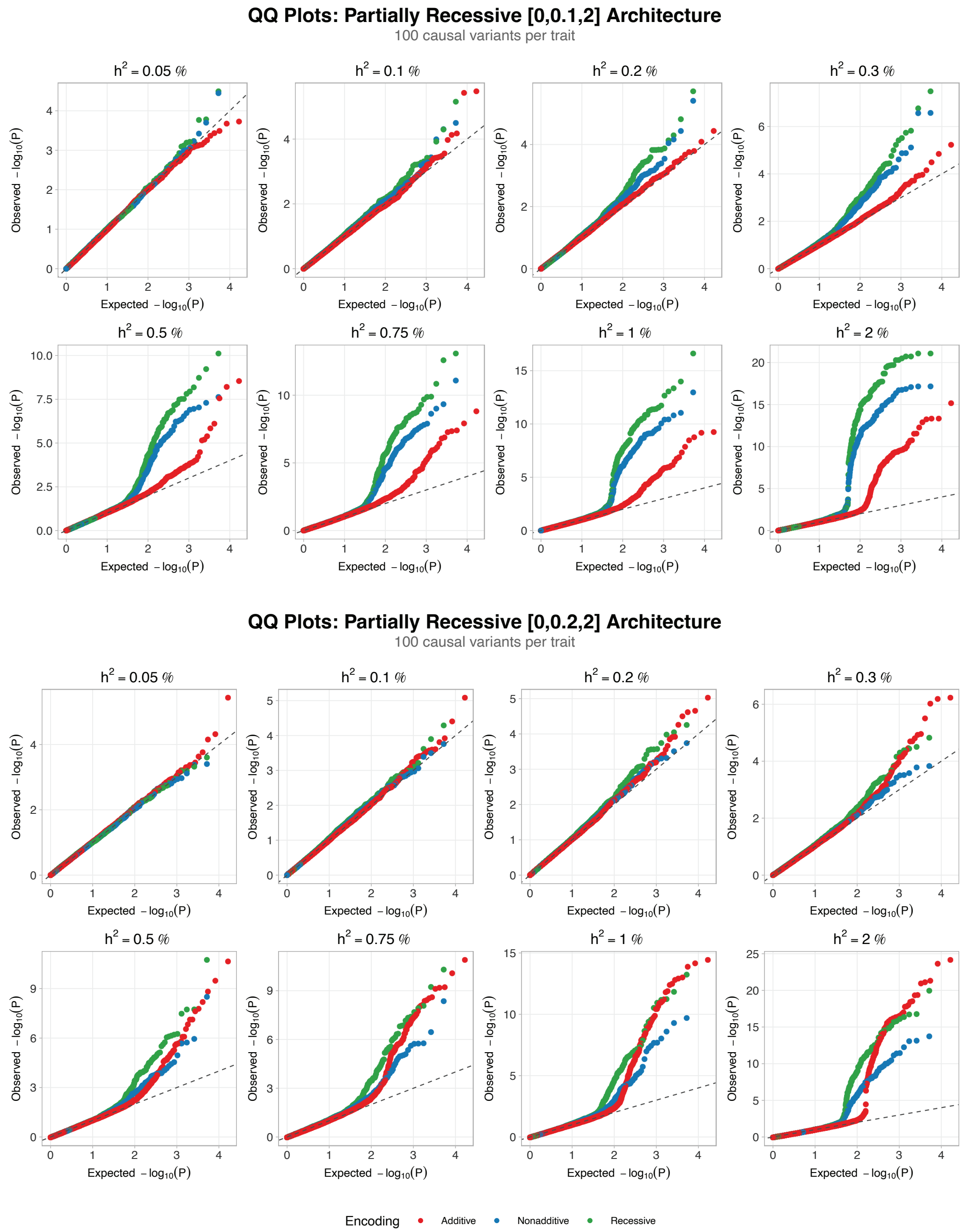

Supplementary Figure 10: QQ-plots comparing SAIGE pseudo-variant test statistics under partially recessive architectures. QQ-plots showing observed versus expected $-\log_{10}(P)$ values from SAIGE gene-level association tests. Phenotypes were simulated using causal pseudo-variants (100 variants per trait) under partially recessive architectures: (Top) $[0, 0.1, 2]$ where heterozygotes contribute 5% of the homozygote effect; and (Bottom) $[0, 0.2, 2]$ where heterozygotes contribute 10%. Points represent the genotype encoding used: additive (red), nonadditive (blue), and recessive (green).

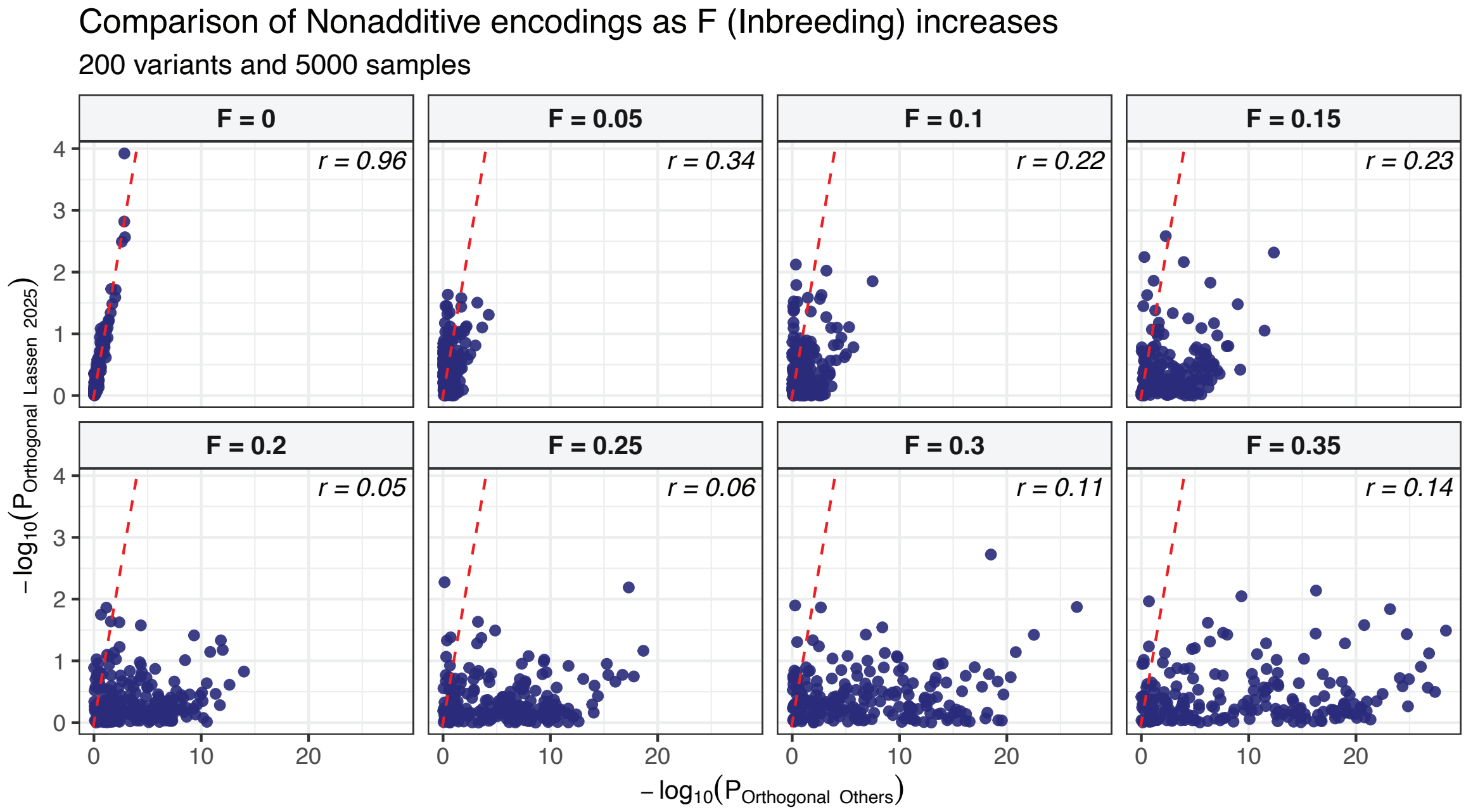

Supplementary Figure 11: Impact of inbreeding on nonadditive test statistic orthogonality. Scatter plots comparing nonadditive association significance ($-\log_{10} P$) between the standard HWE-based encoding ($[-p/(1-p), 1, -(1-p)/p]$) on the x-axis and our proposed orthogonal encoding on the y-axis across increasing levels of inbreeding (F). Phenotypes were simulated with a strong additive effect but no nonadditive effect (i.e. simulated under the null hypothesis). At $F = 0$ (HWE), both methods perform identically (points lie on the diagonal). As inbreeding increases ($F > 0$), the standard encoding fails to isolate the nonadditive component, resulting in false positives due to 'spillover' of the additive signal. In contrast, the proposed HWE-independent method correctly maintains orthogonality, keeping P-values consistent with the null hypothesis. The Pearson correlation (r) is shown in the top right of each panel.

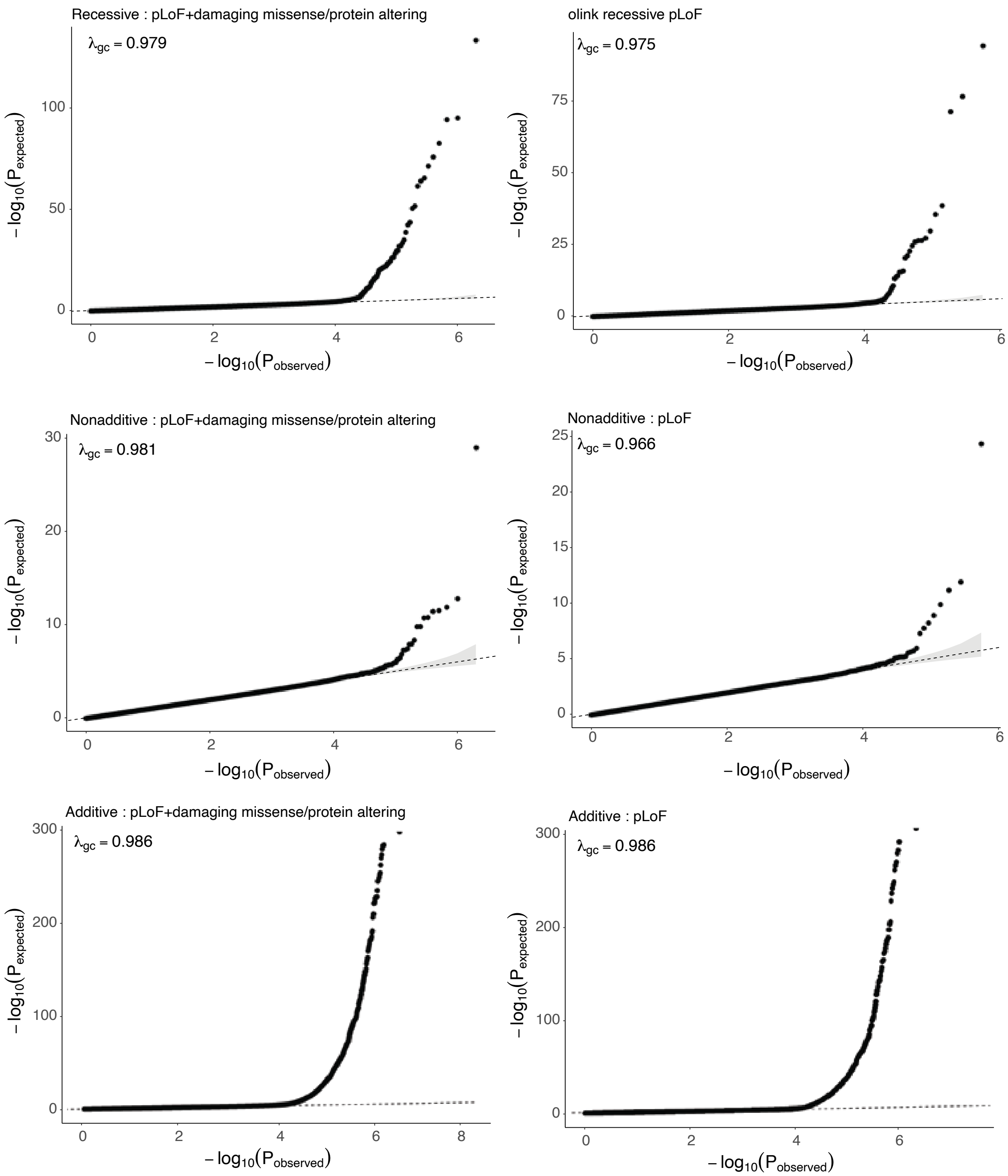

Supplementary Figure 12: QQ-plot for additive, recessive, and nonadditive analysis of Olink data. QQ-plots for Olink data using recessive, additive, and nonadditive genotype encodings (rows, top to bottom). Analysis covers pLoF+damaging missense/protein altering (first column) and pLoF (second column) annotation masks. The corresponding λ_{GC} is shown in the top left corner for each panel. X-axis shows expected P-values (assuming uniform distribution under null hypothesis); Y-axis shows observed P-values. The gray shading represents the 95% confidence interval under the null hypothesis.

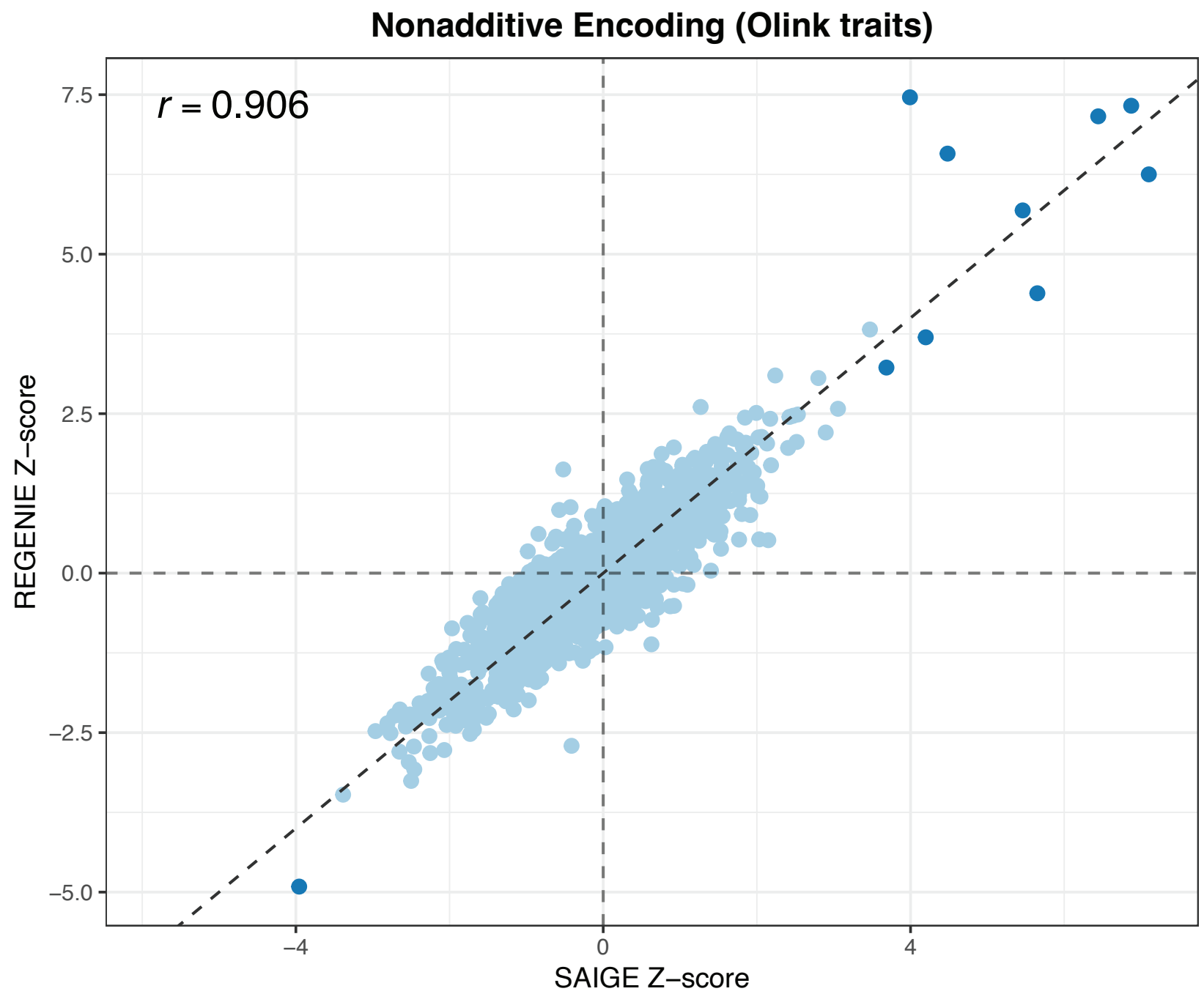

Supplementary Figure 13: Comparison of `SAIGE` and `REGENIE` results for Olink pLoF analysis. Scatter plot comparing $-\log_{10}$ P-values generated by `SAIGE` (x-axis) and `REGENIE` (y-axis). Light blue points represent all tested gene-protein pairs, while dark blue points highlight pairs that achieved statistical significance. The Pearson correlation coefficient (r), displayed in the top left, demonstrates high concordance between the two implementations, confirming that the choice of software does not introduce bias for significant associations.

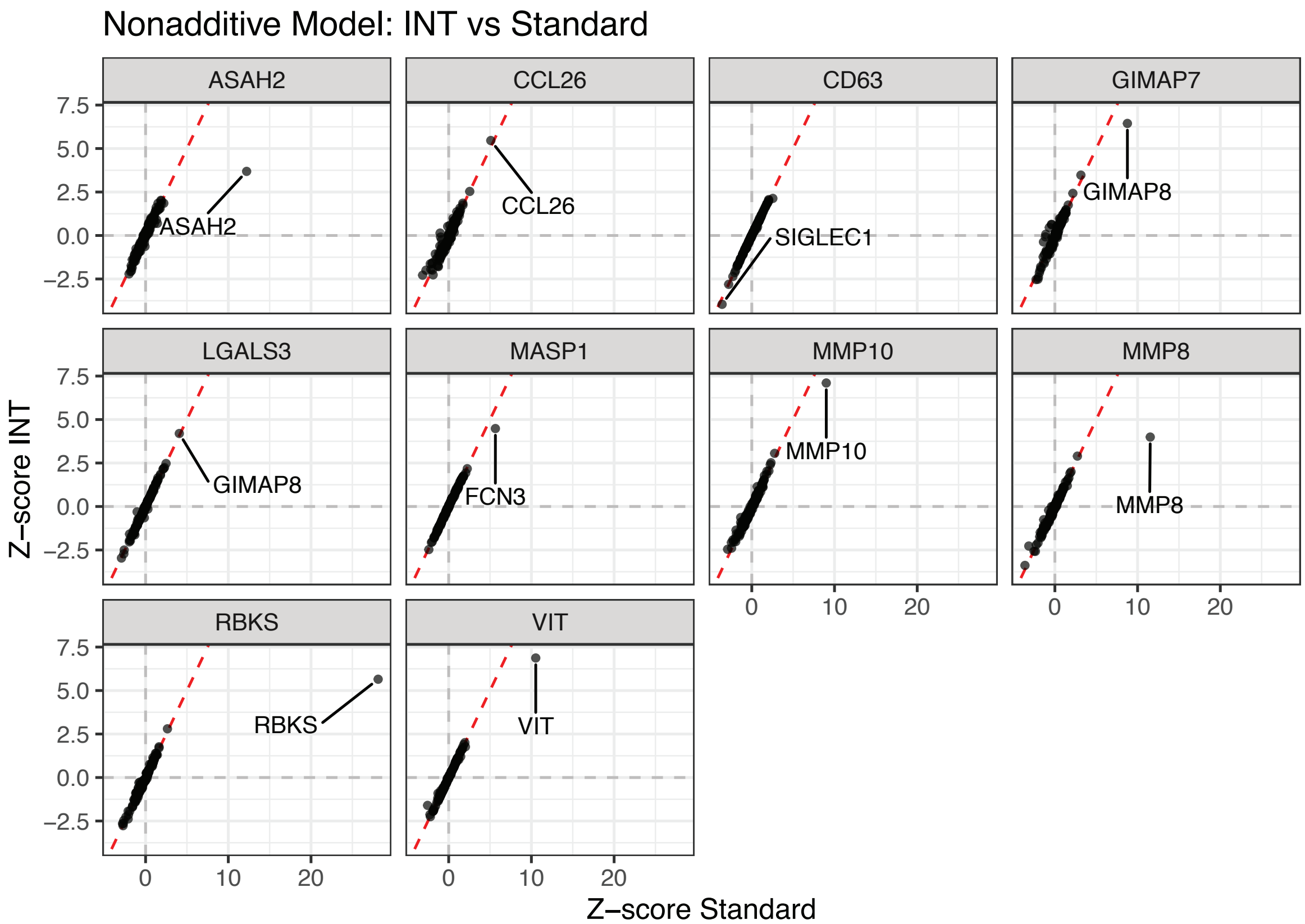

Supplementary Figure 14: Comparison of Inverse Normal Transformation (INT) and standard standardization. Scatter plot comparing protein abundance values processed via INT (y-axis) versus standard standardization (x-axis). The standard transformation scales the raw protein abundance to have a mean of zero and a variance of one, preserving the original distribution shape, whereas INT maps values to a standard normal distribution based on rank.

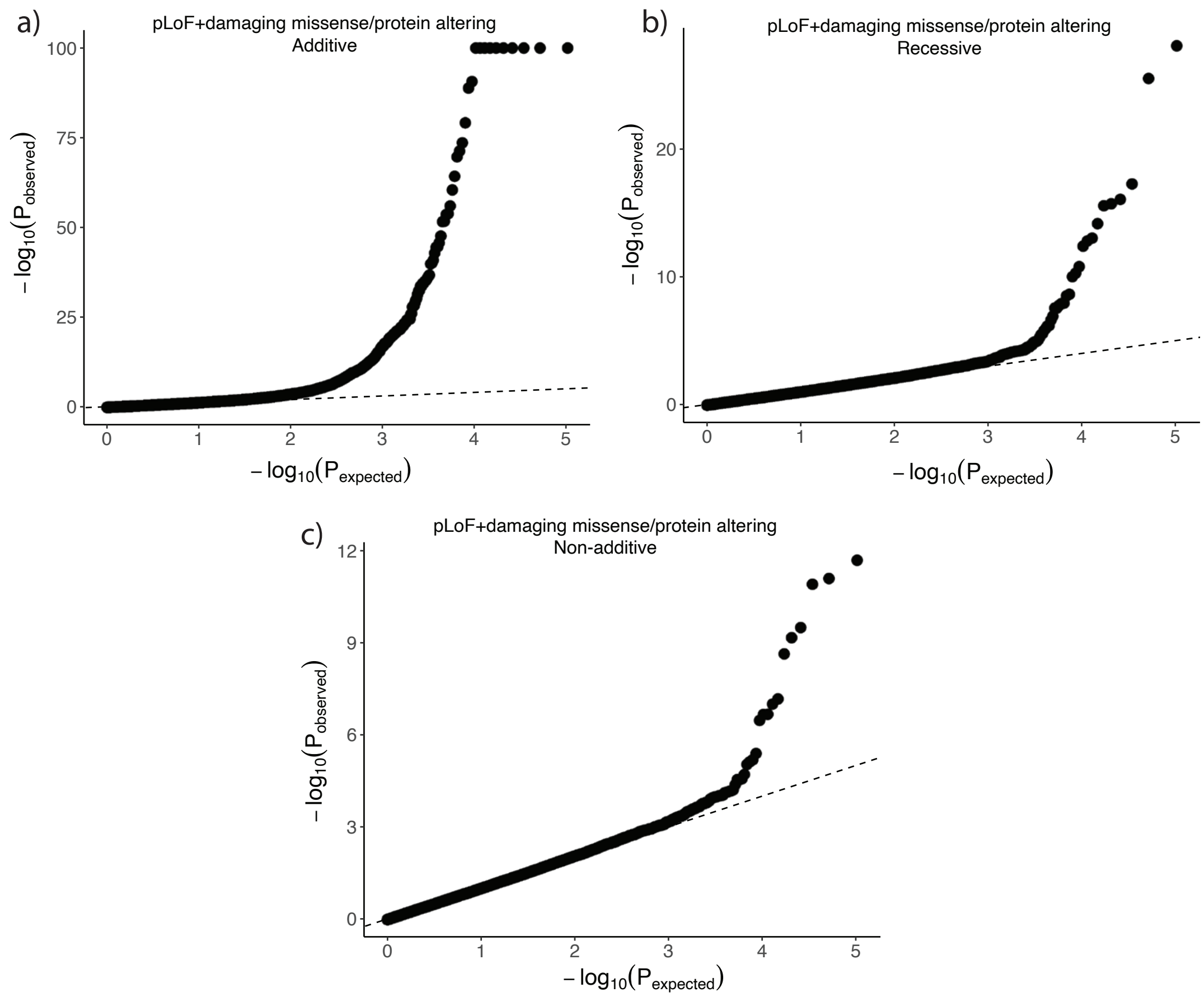

Supplementary Figure 15: QQ-plot for additive, recessive and nonadditive analysis of medically relevant traits. QQ-plots for medically relevant traits using (a) additive, (b) recessive, (c) and nonadditive genotype encodings using the pLoF+damaging missense/protein altering annotation mask. X-axis shows expected P-values (assuming uniform distribution under null hypothesis); Y-axis shows observed P-values. Note that any associations with $P < 10^{-100}$ have been truncated.

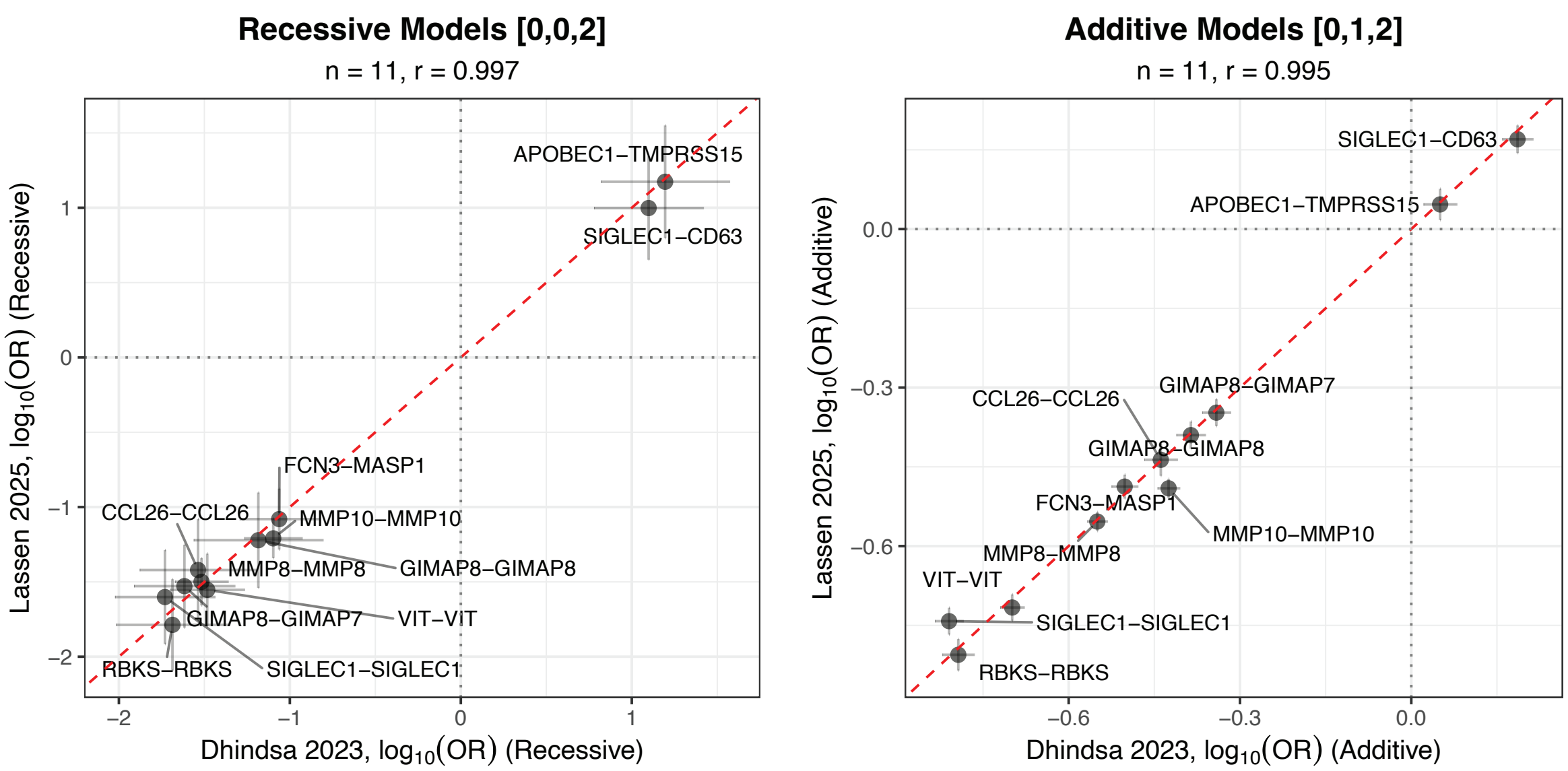

Supplementary Figure 16: Concordance of effect sizes with external replication. Scatter plots comparing the additive (left) and recessive (right) effect sizes estimated in this study (y-axis) against those reported by others[6] (x-axis) for overlapping gene-trait pairs. The strong correlation across both modes of inheritance serves as an external validation, confirming that the proposed 2-DF model accurately recovers established biological effect sizes. Error bars represent 95% confidence intervals.

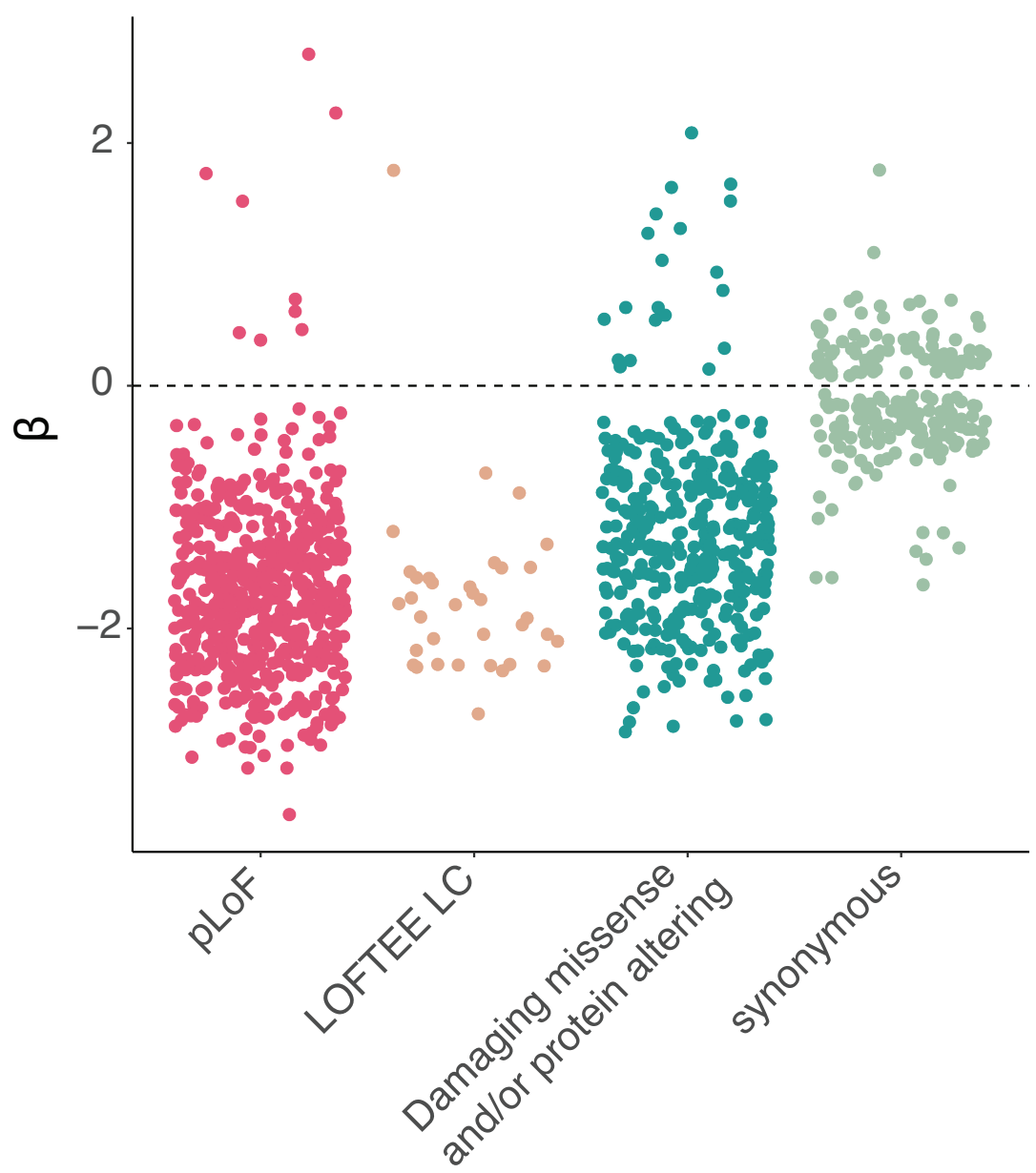

Supplementary Figure 17: Assessment of effect size and direction across additive haplotype-level association analysis. Effect size (β) and direction for significant (Bonferroni $P < 8.33 \times 10^{-10}$) *cis* pQTLs between protein-coding genes and cognate proteins under an additive model. Each point represents a gene-protein pair, colored by variant annotation category as labeled on the x-axis. The dashed horizontal line indicates $\beta = 0$ (no effect).

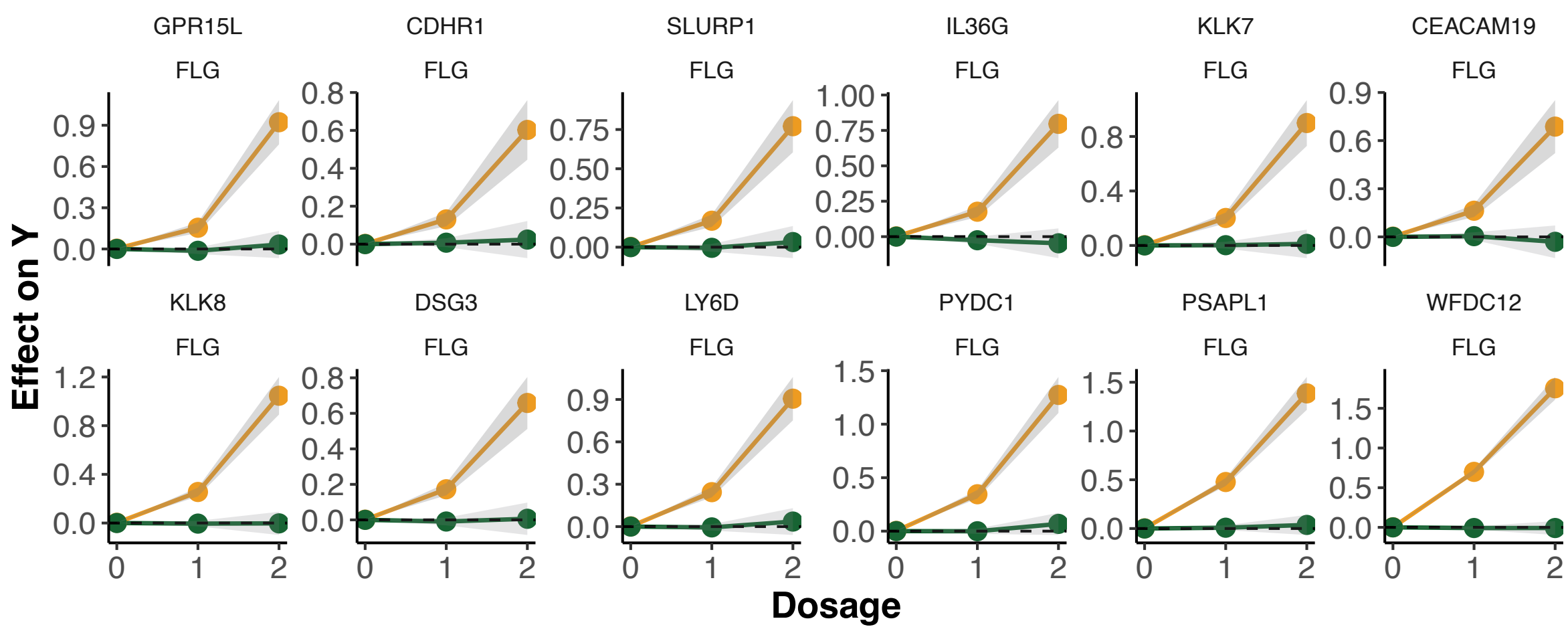

Supplementary Figure 18: Dose-response relationships between *FLG* variants and plasma protein levels. Genetic dosage effects of *FLG* variants on 12 plasma proteins. Each panel shows the relationship between variant dosage (x-axis: 0=wildtype, 1=monoallelic, 2=biallelic carriers) and standardized protein abundance (y-axis). Yellow lines represent functionally deleterious variants (pLoF+damaging missense), while green lines represent synonymous variants. Deleterious *FLG* variants demonstrate consistent dose-dependent increases in protein abundance across all targets, while synonymous variants show no significant effect. Gray shading indicates 95% confidence intervals. This pattern suggests specific trans-regulatory effects of *FLG* loss-of-function on multiple downstream protein pathways.

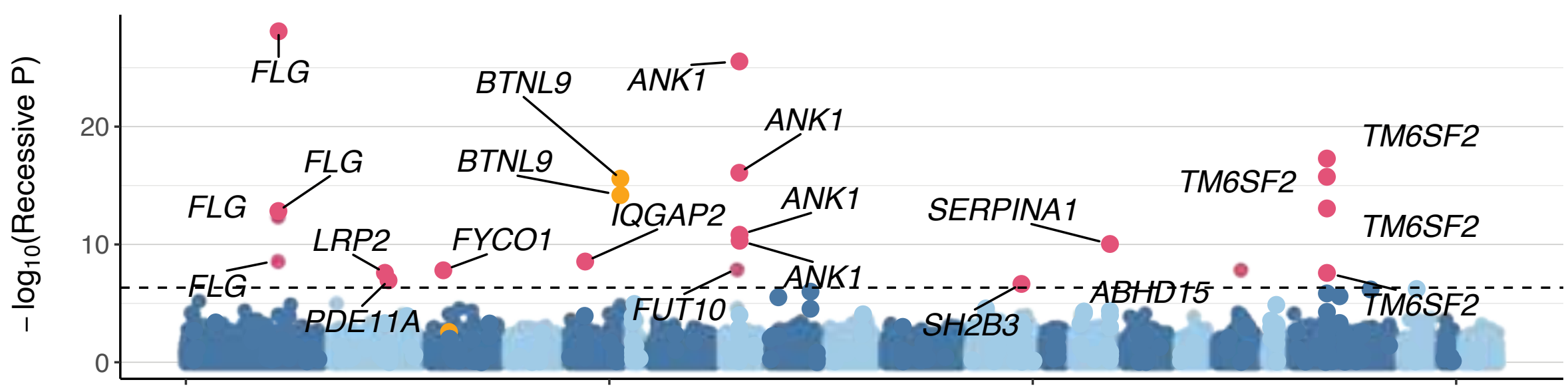

Supplementary Figure 19: Manhattan plot of gene-trait associations using a recessive encoding. Genome-wide associations identified using the recessive encoding $[0, 0, 1]$ across medically relevant traits. Each point represents a gene-trait association with chromosomal position on the x-axis and statistical significance ($-\log_{10}(P)$) on the y-axis. Alternating light and dark blue colors distinguish adjacent chromosomes. The dashed horizontal line represents the Bonferroni-corrected significance threshold. Significant gene-trait associations are labeled with their respective gene names.

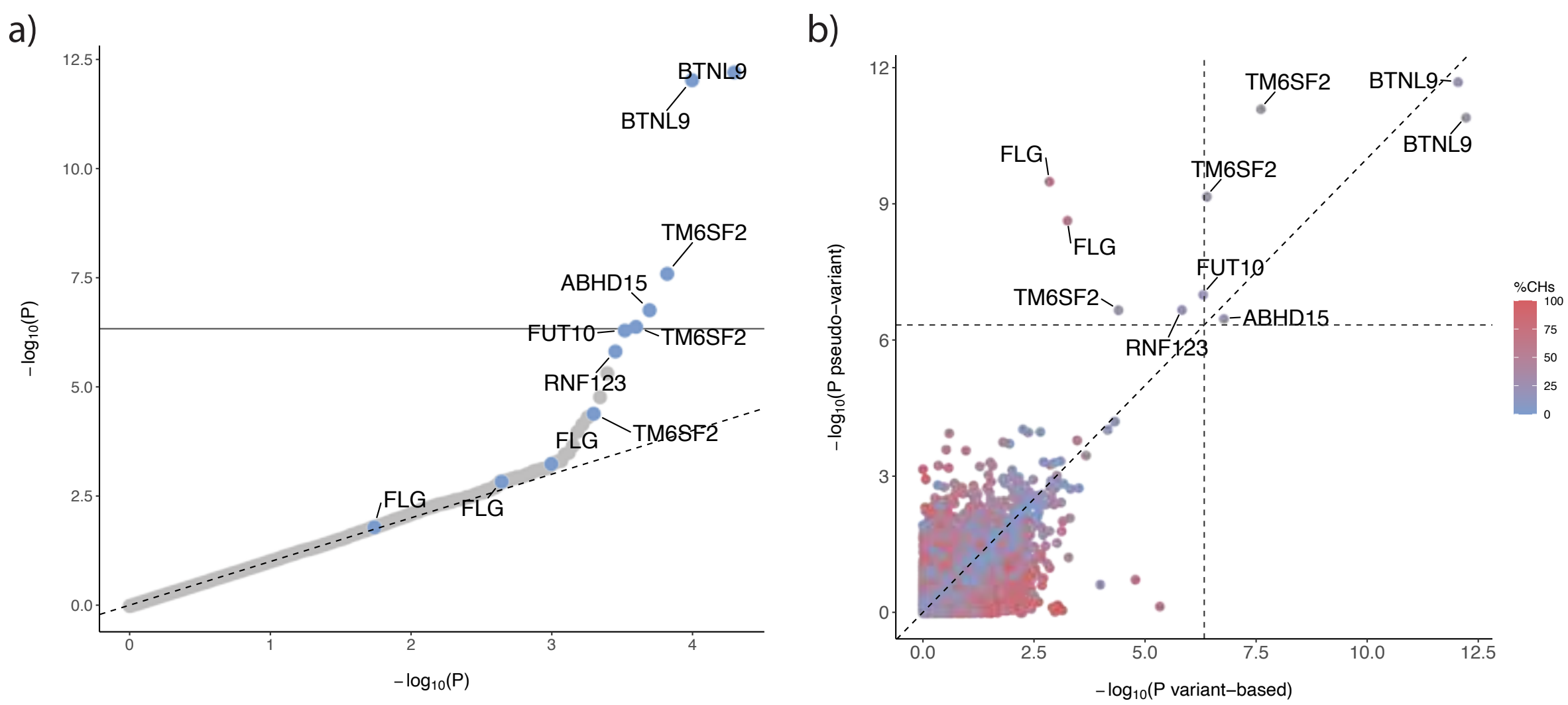

Supplementary Figure 20: Comparison of variant-based burden analysis and pseudo-variant analysis. a) QQ-plot of observed versus expected P-values in the variant-based burden analysis of nonadditive effects. The horizontal line indicates the significance threshold (Bonferroni $P < 3.99 \times 10^{-7}$). Gene-trait associations significant in the original pseudo-variant analysis are highlighted in blue and labeled; non-significant associations are shown in gray. The gray shading represents the 95% confidence interval under the null hypothesis. b) Scatter plot comparing P-values from the pseudo-variant analysis (y-axis) and the variant-based burden analysis (x-axis). Dashed lines represent the significance threshold (Bonferroni $P < 3.99 \times 10^{-7}$). Points are colored by the proportion of biallelic variants identified as CH, ranging from blue (low) to red (high). Significant gene-trait associations from the pseudo-variant analysis are labeled. The line $x = y$ is shown in both plots for reference.
