# Supplementary material for: Deviations from genetic additivity driven by rare variants at biobank scale
Source: Nat Commun. 2026 Aug 29;17:10302. doi: 10.1038/s41467-026-76151-w (PMC13620152; doi:10.1038/s41467-026-76151-w)
Supplement: Supplementary file 4 [file 41467_2026_76151_MOESM2_ESM.pdf]

Description of Additional Supplementary Files

File Name: Supplementary Data 1
Description: Trio switch error rates for 450k exome sequencing following phasing. Trio switch error rates for final reconstituted chromosomes across variants included in the 450k exome sequencing release by minor allele count bins. 'Switches' is the number of errors observed, and 'Tested' is the total number of sites assessed. We include the switch error rates before (phasing posterior probability ≥ 0.50) and after (phasing posterior probability ≥ 0.90) restricting to variants that are phased with high-confidence. All confidence intervals are 95% binomial confidence intervals.

File Name: Supplementary Data 2
Description: Number of mono- and biallelic variants identified across annotation masks in 450k exome sequencing dataset in Europeans. Overview of the number of variants that are present in the 450k exome sequencing release for individuals of European genetic ancestry before and after filtering by phasing posterior probability. Note that CHs indicate compound heterozygous occurrences. Additionally, if two heterozygous variants have been counted as a compound heterozygous or *cis*, they will not be double counted in the 'heterozygous column'.

File Name: Supplementary Data 3
Description: Conditional analysis of significant associations using additive encoding. Results of conditional analysis for significant gene-trait associations, conditioning on nearby common variants (within 500 kb, minor allele frequency ≥ 0.01) using the standard additive encoding. *P*-values before and after conditioning are shown to assess whether rare variant signals are independent of common variant effects. All *P*-values are two-sided, derived from score tests in a linear mixed model (SAIGE) with Bonferroni correction for multiple comparisons.

File Name: Supplementary Data 4
Description: Conditional analysis of significant associations using nonadditive encoding. Results of conditional analysis for significant gene-trait associations, conditioning on nearby common variants (within 500 kb, minor allele frequency ≥ 0.01) using the nonadditive encoding. *P*-values before and after conditioning are shown to assess whether rare variant signals are independent of common variant effects. All *P*-values are two-sided, derived from score tests in a linear mixed model (SAIGE) with Bonferroni correction for multiple comparisons.

File Name: Supplementary Data 5
Description: Head-to-head comparison of additive, recessive and nonadditive *P*-values for Olink putative loss-of-function association analysis. A head-to-head comparison for each gene-trait with a significant additive $[0, 1, 2]$, recessive $[0, 0, 1]$ or nonadditive contribution. All *P*-values are two-sided, derived from score tests in a linear mixed model (SAIGE) with Bonferroni correction for multiple comparisons.

File Name: Supplementary Data 6
Description: Head-to-head comparison of additive, recessive, and nonadditive *P*-values for Olink putative loss-of-function + damaging missense/protein altering variant association analysis. A head-to-head comparison for each gene-trait with a significant additive $[0, 1, 2]$, recessive

$[0, 0, 1]$ or nonadditive contribution. All P-values are two-sided, derived from score tests in a linear mixed model (SAIGE) with Bonferroni correction for multiple comparisons.

File Name: Supplementary Data 7
Description: Summary of the effects of variant categories on cognate protein abundance, based on additive haplotype-specific association analyses. Analyses between genes with at least 10 biallelic carriers and their cognate proteins. The analysis was restricted to 42,217 Europeans with Olink proteomic data. The number of significant associations between a gene and the abundance of the cognate protein (N) is displayed alongside the percentage of total assays that are significant. We stratify by direction of effect (β) and functional category. Note that the 'Total Assays' is different across variant categories as the number of biallelic carriers differ for each gene-assay pair, and as a result, does not always pass the haplotype count criterion (i.e. at least 10 affected haplotypes required).

File Name: Supplementary Data 8
Description: Overview of traits included in the study. Overview of the 55 quantitative traits that have been included in the study.

File Name: Supplementary Data 9
Description: Replication of significant gene-trait associations in All of Us. Replication results for gene-trait associations that reached significance in UK Biobank. Analyses were performed in individuals of European and Admixed-American genetic ancestry using homozygous carriers only, as phased genotypes were unavailable to identify compound heterozygous carriers. Additive, recessive, and nonadditive P-values are shown alongside carrier counts. All P-values are two-sided, derived from score tests in a linear mixed model (SAIGE).

File Name: Supplementary Data 10
Description: Head-to-head comparison of additive, recessive, and nonadditive P-values for medically relevant traits. A head-to-head comparison for each gene-trait with a significant additive $[0, 1, 2]$, recessive $[0, 0, 1]$ or nonadditive contribution. All P-values are two-sided, derived from score tests in a linear mixed model (SAIGE) with Bonferroni correction for multiple comparisons.

File Name: Supplementary Data 11
Description: Joint 2-degree-of-freedom and Cauchy-combined P-values for significant gene-trait associations. Gene-trait associations reaching significance ($P < 4.45 \times 10^{-7}$) in either the joint 2-degree-of-freedom model or the Cauchy-combined test. Columns include gene symbol, trait, allele count, heterozygous and homozygous carrier counts, component P-values (additive, recessive, nonadditive), joint 2-degree-of-freedom P-value, and Cauchy-combined P-value. All P-values are two-sided, derived from score tests in a linear mixed model (SAIGE) with Bonferroni correction for multiple comparisons.
